# Evaluating the Aqueous Stability of Alkyl‐/Aryl‐Hydrosilanes by NMR Spectroscopy and GC‐MS

**DOI:** 10.1002/cplu.202500310

**Published:** 2025-07-21

**Authors:** Fawwaz Azam, Marc J. Adler

**Affiliations:** ^1^ Department of Chemistry & Biology Toronto Metropolitan University 350 Victoria St. Toronto ON M5B2K3 Canada

**Keywords:** aqueous stability studies, bioisostere, hydrosilane stability, medicinal chemistry, organosilanes

## Abstract

Hydrosilanes are commonly used as reducing agents or as synthetic precursors for silanols. However, the incorporation of hydrosilanes as carbon bioisosteres is underexplored. In this study, the hydrolytic stability of ten variably substituted hydrosilanes—including monoaryl, monoalkyl, diaryl, dialkyl, alkyl aryl, triaryl, trialkyl, dialkyl aryl, and alkyl diaryl silanes—is investigated using five complementary methods, including ^1^H–NMR time‐lapse and GC‐MS experiments, at neutral pH. The ^1^H–NMR time‐lapse experiments suggest that monoaryl and monoalkyl silanes are susceptible to hydrolysis, as evidenced by 31% and 22% reduction in starting material, respectively, over 24 h. Other investigated silanes are resistant to hydrolysis in these solvent systems for at least 24 h. The GC‐MS experiments quantitatively support the respective reactivity of these hydrosilanes at pH 7. Lastly, the reactivity of selected hydrosilanes is evaluated at pH 7.4 phosphate‐buffered saline buffer; only monoalkyl silanes degraded in the presence of the added salt content. Overall, the study demonstrates that hydrosilanes exhibit hydrolytic stability at neutral pH, except for monoaryl‐ and monoalkyl‐substituted silanes, which are susceptible to degradation. The results provide insight into the likelihood of the Si—H bond surviving in aqueous environments, opening the door for a wider variety of silicon‐containing molecules in drug discovery.

## Graphical abstract

The incorporation of hydrosilanes as a carbon bioisostere in bioapplied molecules is under‐explored. In this study, the aqueous stability at neutral pH of representative alkyl‐/aryl‐hydrosilanes is investigated using NMR spectroscopy and GC‐MS.The aggregate data analysis reveals which Si‐H bonds are susceptible to hydrolysis, which will inform future organosilane design in bioapplied molecules.

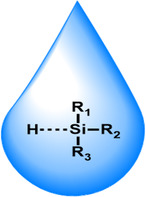

## 1 Introduction

Silicon is the second most abundant element in Earth's crust and is found in molecules used for a broad range of applications, from electronics to organic synthesis. Silicon is located beneath carbon in the periodic table and shares similar properties with it: for example, they each form tetracoordinate species with stable bonds to carbon, oxygen, and hydrogen. However, there are also differences between silicon and carbon. Bonds to silicon are longer (compared to those with carbon) due to silicon's larger covalent radius; for example, the C(sp_3_)—Si (1.86 Å) bond is about 20% longer than the C(sp_3_)—C(sp_3_) (1.53 Å) bond. Further, unlike their carbon analogues, hydrosilanes—compounds possessing at least one Si—H bond—have hydridic properties due to silicon's lower electronegativity (Pauling scale) value relative to carbon (Si = 1.90, C = 2.55, H = 2.20); this property dictates their primary chemical reactivity. Both of these attributes make silicon generally more reactive than carbon.

This reactivity has been harnessed to develop a variety of hydrosilane reagents, which have been used to carry out diverse chemical transformations. They can be used to create new Si—C bonds in a hydrosilylation reaction, typically catalyzed by a metal catalyst such as Pt, Rh, Ir, or Ru.^[^ ^1^ ^]^ Hydrosilanes can also be used as reducing agents for carbonyl compounds and in direct reductive aminations.^[^ ^2^, ^3^, ^4^, ^5^, ^6^ ^]^ Hydrosilanes have been employed as stoichiometric amide coupling reagents. This reaction proceeds through a silyl ester intermediate to form amide, H_2_, and a siloxane byproduct.^[^ ^7^, ^8^ ^]^ Some recent examples of organosilane coupling reagents for amidation include PhSiH_3_,^[^ ^9^ ^]^ Ph_2_SiH_2_,^[^ ^10^, ^11^ ^]^ and HSi(OCH(CF_3_)_2_)_3_.^[^ ^12^ ^]^ Hydrosilanes can also undergo oxidation to form silanols or siloxanes.^[^ ^13^, ^14^, ^15^, ^16^, ^17^, ^18^, ^19^, ^20^, ^21^, ^22^ ^]^ For this reason, they are commonly used as precursors to synthesize polymeric materials. Silanols have also been used as coupling partners for C—C cross‐coupling reactions.^[^ ^23^ ^]^

**Figure 1:**
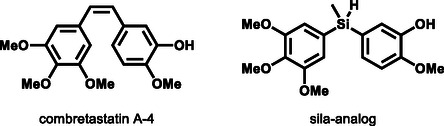
Combretastatin A‐4 and the hydrosilane analog.

Another aspect of silicon is its use as a carbon bioisostere in medicinal chemistry;^[^ ^24^ ^]^ this is because of the electronic similarities between carbon and silicon, the low inherent toxicity of silicon, and the differences between silicon and carbon that provide opportunities for subtle control of drug interaction.^[^ ^25^ ^]^ Currently, the incorporation of silicon as a carbon bioisostere is an underdeveloped area of research. The few examples of sila‐analogs of a drug in the literature are all silanols/silane‐diols or quaternary organosilanes (i.e., a silicon bonded to four substituents, each via an Si—C bond).^[^ ^25^ ^]^ To the best of our knowledge, there has only been one published example of a hydrosilane in medicinal chemistry: the sila‐analog of combretastatin A‐4, a bioactive natural product (**Figure** **1**).^[^ ^26^ ^]^ This is surprising given the number of methine, methylene, and methyl groups that could be subjected to a C/Si swap in any number of bioactive molecules to impact their efficacy, pharmacokinetic profile, and physicochemical properties.

Researchers have developed multiple reactions to convert the hydride(s) in hydrosilanes to other functional groups (e.g., OH to make silanols). However, there is a conspicuous scarcity of examples of hydrosilanes being used for biological applications, which involve exposure to moisture. Additionally, Material data safety sheet (MSDS) labels for hydrosilanes typically indicate their sensitivity to water. For example, the MSDS for triphenylsilane lists “exposure to moist air or water” under “conditions to avoid”. Notably, for all the silanes investigated in the paper, we found at least one source that seems to indicate instability to water.^[^ ^27^ ^]^

Given the unique properties of hydrosilanes (e.g., stability, tunable reactivity), their underuse in C/Si switch in bioapplied molecules, and the ambiguous inferences one might gain about the aqueous stability of such molecules, we set out to systematically investigate the reactivity of hydrosilanes in aqueous systems to determine the stability of Si—H bonds. This portends the potential for investigation of a wider variety of bioactive molecules containing silicon in place of carbon for application in drug discovery or otherwise. Specifically, we wanted to understand: 1) how the number of Si—H bonds and 2) how aryl or alkyl substitutions affect the aqueous stability of the Si—H bond.

## 2 Results and Discussion

**Figure 2:**
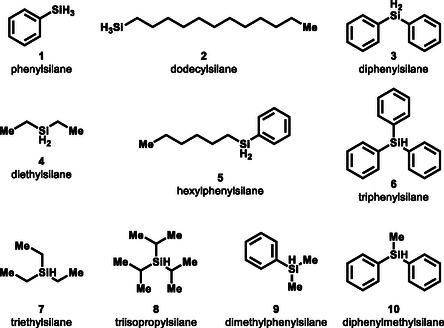
Hydrosilane substrates were examined for aqueous stability.

Ten hydrosilanes were selected as substrates for the experiments (**Figure** **2**): PhSiH_3_ **1**, dodecylSiH_3_ **2**, Ph_2_SiH_2_ **3**, Et_2_SiH_2_ **4**, *n*‐hexylphenylSiH_2_ (Hex(Ph)SiH_2_) **5**, Ph_3_SiH **6**, Et_3_SiH **7**, i‐Pr_3_SiH **8**, Me_2_PhSiH **9**, and Ph_2_MeSiH **10**.

The ten substrates selected cover a range of substitutions on the silicon: monoaryl, monoalkyl, diaryl, dialkyl, alkyl aryl, triaryl, trialkyl, dialkyl aryl, and alkyl diaryl. These specific ten hydrosilanes were selected to cover the general intended scope of study due to their commercial availability and/or ease of preparation. The overall stability (at neutral pH) of these hydrosilanes were investigated using a combination of five experiments: 1) hydrosilanes were dissolved in either in 10% D_2_O:MeOD‐d_4_ or in 10% D_2_O:DMSO‐d_6_, and then proton nuclear magnetic resonance (^1^H–NMR) spectroscopy was used to monitor the Si—H bond and any new signals present to determine the silane's stability; 2) hydrosilanes were reacted with H_2_O for 1 h then recovered via liquid/liquid extraction. The identity and amount of the silane recovered would reveal the reactivity of the hydrosilane in water; 3) hydrosilanes were reacted with H_2_O for 1 h and then recovered by direct removal of solvent in vacuo. This experiment would allow us to directly observe the species present at the reaction end point; 4) hydrosilanes were reacted with H_2_O for 1 h and then recovered via liquid/liquid extraction. The organic layer was directly analyzed using gas chromatography‐mass spectrometry (GC‐MS) to identify and quantify the amount of silane at the reaction endpoint. This approach allows for the quantification of unreacted hydrosilane without the need for an intermediate rotary evaporation step, which could potentially lead to loss of starting material; and 5) select hydrosilanes were added to a PBS buffer solution (pH 7.4) for 1 h and then recovered via liquid/liquid extraction. This experiment would test the effect of biorelevant salts on the aqueous stability of the hydrosilanes.

While in theory each of these experiments could provide the answer to our query independently, each experimental setup has its own technical benefits and drawbacks; aggregate analysis of the resulting data from all experiments allows us to feel confident in the conclusions. All five studies focus on assessing the reactivity of each hydrosilane by quantifying the amount of starting material remaining after exposure to water. We did not characterize the hydrolysis products, as our interest was solely in Si—H bond stability. It should be noted that aqueous solutions of hydrosilanes were only completely homogenous in Study 1, as it was required for accurate in situ assessment via NMR; in the other studies, the silanes were well‐dispersed heterogeneous mixtures of silane with water, with direct GC analysis showing evidence of dissolved silane in water (unquantified).

### 2.1 Reactivity in Aqueous Mixtures Monitored In Situ using ^1^H–NMR (Study 1)

The representative silanes selected did not all readily dissolve in pure D_2_O, and therefore, a mixed deuterated solvent system was required to ensure homogenous solutions and thus accurate assessment of these experiments using ^1^H–NMR. To decide on the solvent system, the hydrosilane was dissolved in a water‐miscible organic solvent, and then increasing amounts of H_2_O were added until cloudiness was observed; we found that a 10% D_2_O in MeOD‐d_4_ system worked best as a general system. In the singular case of dodecylsilane, a 10% D_2_O in DMSO‐d_6_ was used. A concentration of 10% D_2_O represents a large excess (over 90 eq.) of D_2_O relative to the hydrosilane, which provides ample opportunity for a reaction (regardless of organic cosolvent). The results from this ^1^H–NMR study are presented in **Table** **1**.

**Table 1:** Reactivity of hydrosilane in 10% D_2_O in MeOD‐d_4_ as monitored by ^1^H–NMR over 24 h.

Entry	Class of silane	Substitution	Hydrosilane	Remaining hydrosilane [%]a)
1	Trihydro	Aryl	PhSiH_3_ **1**	69
2		Alkyl	C_12_H_25_SiH_3_ **2**	78b)
3	Dihydro	Aryl	Ph_2_SiH_2_ **3**	96
4		Alkyl	Et_2_SiH_2_ **4**	98
5		Alkyl aryl	Hex(Ph)SiH_2_ **5**	98
6	Monohydro	Aryl	Ph_3_SiH **6**	100
7		Alkyl	Et_3_SiH **7**	100
8		Alkyl	i‐Pr_3_SiH **8**	100
9		Dialkyl aryl	Me_2_PhSiH **9**	100
10		Alkyl diaryl	Ph_2_MeSiH **10**	100

a) To an NMR tube was added the silane (≈5 mg) and MeOD‐d_4_ (0.9 mL) or DMSO‐d_6_ (0.9 mL). After sonication for 30 s, D_2_O (0.1 mL) was added, and the NMR tube was shaken, followed immediately by ^1^H–NMR acquisitions at set intervals: at 0, 10, 60 min, and 24 h.

b) The silane was added to DMSO‐d_6_ instead of MeOD‐d_4_ due to solubility issues.

**Figure 3:**
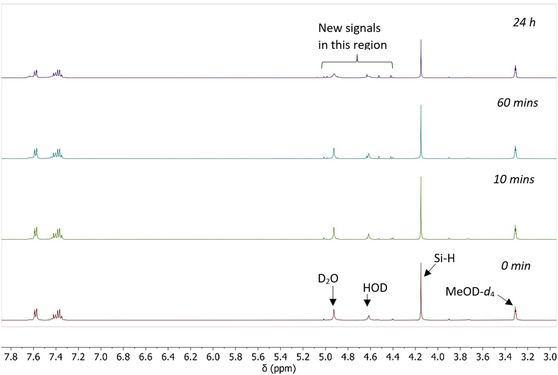
^1^H–NMR snapshots of PhSiH_3_ in 10% D_2_O in MeOD‐d_4_ over 24 h.

Silane **1** begins to degrade within 10 min in the aqueous solvent (Table 1, entry 1). The time‐lapse ^1^H–NMR shows a steady decrease in intensity for the Si—H signal at 4.15 ppm along with a concomitant increase in novel signals in the 4–5 ppm region (**Figure** **3**). This suggests that trihydrosilane **1** is being hydrolyzed into a mixture of mono‐, di‐, and/or tri‐hydroxyphenylsilanes, and polysiloxanes. Over the course of 24 h, the concentration of **1** (as monitored by the area under the integral of the Si—H signal at 4.15 ppm) decreased by 31% relative to the amount of starting material. Similarly, the concentration of **2** decreased by 22%.

On the other hand, no/minimal reaction was observed for the remaining hydrosilanes. Overall, the ^1^H–NMR time‐lapse experiments suggest that monoaryl and monoalkyl silanes are readily susceptible to hydrolysis under these mixed‐solvent conditions, whereas diaryl‐, dialkyl‐, alkylaryl‐, triaryl‐, trialkyl‐, dialkylaryl‐, and alkyldiaryl silanes are resistant to hydrolysis in these solvent systems for up to at least 24 h.

### 2.2 Reactivity in Water Evaluated using ^1^H–NMR (Studies 2 and 3)

We wanted to further probe the reactivity of the hydrosilanes in water alone (i.e., without organic cosolvent), and since the solubility of the representative silanes precluded this analysis via in situ ^1^H–NMR, we devised a range of alternative ^1^H–NMR‐based experiments. Each hydrosilane was stirred in water and then extracted with ethyl acetate (EtOAc) to recover the organic compounds. The organic solvent was separated, and then the solution was concentrated, and the resulting crude mixture was re‐dissolved in CDCl_3_. ^1^H–NMR provided evidence for whether a reaction between the hydrosilane and water had taken place. In order to draw conclusion on the hydrosilane's reactivity in water, we decided to also observe the leftover material by directly removing water in vacuo (without an intermediary liquid/liquid extraction step). Of note, five out of ten hydrosilanes used (**1**, **4**, **7**, **8**, and **9**) were volatile (**Table** **2**);^[^ ^28^ ^]^ their volatility means there is potential loss of material due to evaporation under ambient conditions or under vacuum during manipulation.

Silane **1** was recovered as pure starting material in 29% recovery (Table 2, entry 1). Since the time‐lapse ^1^H–NMR experiment for **1** shows that the Si—H bond degrades in 10% D_2_O in MeOD‐d_4_ over 24 h, it is not surprising that a low recovery of **1** is observed. The remaining mass balance is likely in the aqueous layer as hydrolysis by‐products and lost to evaporation (BP: 120 °C) during concentration. When **2** was reacted with H_2_O, the workup afforded a 93% recovery of the starting material (Table 2, entry 2). This high recovery suggests that dodecylsilane could be stable in water, and that the reactivity observed in the ^1^H–NMR experiment (Table 1, entry 2) was due to the mixed solvent conditions used (DMSO‐d_6_ was used instead of MeOD‐d_4_).

**Table 2:** Reactivity of hydrosilane in distilled water as determined by both extraction and direct removal of water in vacuo.

Entry	Class of silane	Substitution	Hydrosilane	Boiling point	Recovery after extraction [%]	Result from direct evaporation of watera), b)
1	Trihydro	Aryl	PhSiH_3_ **1**	120 °C	29c)	Starting material not observed, possible hydrolysis by‐products observed
2		Alkyl	C_12_H_25_SiH_3_ **2**	80 °C @ 7 mm Hg	93	Only starting material observed
3	Dihydro	Aryl	Ph_2_SiH_2_ **3**	95–97 °C @ 13 mm Hg	93	Only starting material observed
4		Alkyl	Et_2_SiH_2_ **4**	56 °C	3c)	Suspected evaporation
5		Alkyl aryl	Hex(Ph)SiH_2_ **5**	87–89 °C @ 2 mm Hg	76	Only starting material observed
6	Monohydro	Aryl	Ph_3_SiH **6**	152 °C @ 2 mm Hg	100	Only starting material observed
7		Alkyl	Et_3_SiH **7**	107–108 °C	32c)	Suspected evaporationd)
8		Alkyl	i‐Pr_3_SiH **8**	169–170 °C	36c)	Suspected evaporationd)
9		Dialkyl aryl	Me_2_PhSiH **9**	157 °C @ 744 mmg Hg	42c)	Suspected evaporationd)
10		Alkyl diaryl	Ph_2_MeSiH **10**	91 °C @ 1 mm Hg	82	Only starting material observed

a) To a flame‐dried flask under nitrogen was added the silane (0.25 mmol), followed by distilled H_2_O (2.5 mL, 0.1 M). The reaction was stirred under nitrogen for 1 h, and then extracted using 5 × 5 mL EtOAc. The combined organic fraction was dried over MgSO_4_, then concentrated using a rotary evaporator. The percent recovery was calculated based on the mass remaining after drying. The sample was dissolved in the appropriate deuterated solvent for ^1^H–, ^13^C–, and ^29^Si–NMR acquisition.

b) To a flame‐dried flask under nitrogen was added silane (0.25 mmol) followed by distilled H_2_O (1 mL, 0.25 M). The reaction was stirred under nitrogen for 1 h and then concentrated under vacuum to remove H_2_O. The sample was reconstituted in the appropriate deuterated solvent for NMR acquisition.

c) To minimize loss of material due to the rotatory evaporator, a modified extraction was used for the volatile hydrosilanes (**1**, **4**, **7**, **8**, and **9**): the reaction was cooled to 0 °C, MgSO_4_ powder was added, followed by 1 eq. of mesitylene internal standard and CDCl_3_. The entire mixture was sonicated for 3 min before transferring the liquid into an NMR tube for NMR analysis.

d) The ^1^H–NMR of the sample only showed the presence of deuterated solvent. It is likely that the silanes were lost during the removal of H_2_O under high vacuum.

For the dihydrosilanes, **3** and **5** were recovered in yields of 93% and 76%, respectively (Table 2, entries 3, 5). This is consistent with the time‐lapse ^1^H–NMR data supporting their stability in water over 24 h. Poor recovery was obtained for **4** (Table 2, entry 4) was likely due to its volatility (BP: 56 °C) and not due to reaction with water, given that no reaction took place in the ^1^H–NMR experiments. Silane **6** was quantitatively recovered, and **10** was recovered in 82%, suggesting that little to no reaction with water had taken place, consistent with their time‐lapse ^1^H–NMR data (Table 2, entry 6, 10). The remaining three mono‐hydrosilanes’ (**7**, **8**, **9**) percent recovery is 32%, 36%, and 42% respectively (Table 2, entries 7, 8, 9); it is likely that the mass balance was lost to evaporation during concentration (respective BPs: 107, 169, and 157 °C).

To confirm that the remaining mass balance of the hydrosilanes from the aqueous workup is unreacted starting material lost to the aqueous phase, each hydrosilane was stirred in H_2_O for 1 h, and then the solvent was removed in vacuo. The leftover material was redissolved in CDCl_3_ or DMSO‐d_6_ and analyzed using ^1^H–NMR to determine the identity of the leftover material. At the onset we suspected that it would be challenging to observe the five volatile hydrosilanes (**1**, **4**, **7**, **8**, **9**) following solvent removal in vacuo, and the results confirm this as none of the silanes were recovered (Table 2, entries 1, 4, 7, 8, 9). This also suggests that there is no reaction of the hydrosilanes in each of these cases, as hydrolysis of the silanes would lead to high‐boiling siloxanes that would be observed in the ^1^H–NMR spectrum of each. In the case of **1**, the parent Si—H signal is absent, new signals (singlets) are present in the 4–6 ppm region (representing novel Si—H‐containing species), and a broadening of the aromatic signals is also observed. The identity of the leftover material was not characterized, but a polysiloxane likely formed as a result of hydrolysis. Silane **2** was observed as the sole species after evaporation of water in its trial (Table 2, entry 2). Nonvolatile dihydrosilanes (aryl **3** and alkyl aryl **5**) were observed as the sole species after concentration (Table 2, entries 3, 5); nonvolatile monohydrosilanes **6** and **10** were also observed as the sole species following their respective exposures to water and subsequent concentration (Table 2, entries 6, 10).

### 2.3 Reactivity in Water Evaluated using GC‐MS (Study 4)

We wanted to evaluate the reactivity of hydrosilanes using GC‐MS to take advantage of the highly quantitative nature of this analytical tool. For these experiments, each hydrosilane was stirred in H_2_O for 1 h and then extracted with EtOAc. The resulting organic solution was directly analyzed by GC‐MS using a calibration curve to quantify the recovered hydrosilane (**Table** **3**). High recoveries were observed for the nonvolatile hydrosilanes dodecylsilane (**2**), Ph_2_SiH_2_ (**3**), Hex(Ph)SiH_2_ (**5**), and Ph_3_SiH (**6**) (Table 3, entries 2, 3, 5, 6). The results provide further evidence that these hydrosilanes do not degrade in water. For the volatile silanes (**1**, **4**, **7**, **8**, **9**), higher recoveries were observed compared to the results presented in Table 2, suggesting that the previously observed lower recoveries were likely due to loss during rotary evaporation rather than hydrolysis. In the case of PhSiH_3_ (**1**), the 61% recovery observed in the GC‐MS experiment suggests that up to 39% of the compound was lost to degradation (Table 3, entry 1), consistent with the ^1^H–NMR data. Diethylsilane (**4**) was recovered in 13 ± 8.6% yield, and Et_3_SiH (**7**) was recovered in 48 ± 16% yield. Both values are not significantly different from the data presented in Table 2. However, given that their ^1^H–NMR data indicate no reactivity, the low recoveries may be due to evaporation or degradation during the course of analysis rather than degradation due to exposure to water.

**Table 3:** Yield of hydrosilane after liquid/liquid extraction as determined by GC‐MS.

Entry	Class of silane	Substitution	Hydrosilane	Estimated recovery [mmol]	Estimated recovery [%]a)
1	Trihydro	Aryl	PhSiH_3_ **1**	0.31 ± 0.014	61 ± 5
2		Alkyl	C_12_H_25_SiH_3_ **2**	0.48 ± 0.016	96 ± 3
3	Dihydro	Aryl	Ph_2_SiH_2_ **3**	0.50 ± 0.016	100 ± 3
4		Alkyl	Et_2_SiH_2_ **4**	0.06 ± 0.008	13 ± 9
5		Alkyl aryl	Hex(Ph)SiH_2_ **5**	0.45 ± 0.015	91 ± 3
6	Monohydro	Aryl	Ph_3_SiH **6**	0.52 ± 0.008	104 ± 1
7		Alkyl	Et_3_SiH **7**	0.24 ± 0.040	48 ± 16
8		Alkyl	i‐Pr_3_SiH **8**	0.42 ± 0.016	84 ± 4
9		Dialkyl aryl	Me_2_PhSiH **9**	0.44 ± 0.0067	88 ± 2
10		Alkyl diaryl	Ph_2_MeSiH **10**	0.51 ± 0.028	101 ± 6

a) To a vial was added the silane (0.5 mmol), followed by distilled H_2_O (5 mL, 0.1 M). The reaction was stirred for 1 h and then extracted using 25 mL of EtOAc. An aliquot of the organic layer was directly injected into the GC‐MS and the yield of the hydrosilane was calculated using a calibration curve.

### 2.4 Reactivity in Buffered Aqueous Solutions Evaluated using ^1^H–NMR (Study 5)

We were also interested in evaluating the hydrolytic stability of the hydrosilanes with added salt content (PBS buffer, pH 7.4) (**Table** **4**), since this would be important if they are to be used for biological applications. Silanes **1**, **4,** **7**, **8**, and **9** were omitted from this experiment because their low boiling point means that the material would be lost during the workup, as observed previously.

**Table 4:** Percent recovery of hydrosilanes after liquid/.liquid extraction from PBS buffer solution (pH 7.4).

Entry	Class of silane	Substitution	Hydrosilane	Recovery [%]a)
1	Trihydro	Alkyl	C_12_H_25_SiH_3_ **2**	0
2	Dihydro	Aryl	Ph_2_SiH_2_ **3**	93
3		Alkyl Aryl	Hex(Ph)SiH_2_ **5**	88
4	Monohydro	Aryl	Ph_3_SiH **6**	92
5		Alkyl diaryl	Ph_2_MeSiH **10**	92

a) To a flame‐dried flask under nitrogen was added the silane (1 mmol) followed by the PBS solution (10 mL, 0.1 M, pH 7.4). The reaction was stirred under nitrogen for 1 h, and then extracted using 5 × 5 mL EtOAc. The combined organic fraction was dried over MgSO_4_, then concentrated using a rotary evaporator. The percent recovery was calculated based on the mass remaining after drying. The sample was dissolved in the appropriate deuterated solvent for ^1^H–, ^13^C–, and ^29^Si–NMR acquisition.

Dodecylsilane (**2**) fully reacted with water under PBS buffer solution (Table 4, entry 1). This, combined with the result using **2** in Table 2, suggests that the added ions play a role in accelerating hydrolysis. Nonvolatile dihydrosilanes (aryl and alkyl aryl), Ph_2_SiH_2_ (**3**), Hex(Ph)SiH_2_ (**5**), were recovered in 93% and 88% respectively (Table 4, entries 2, 3). The monohydrosilanes (Ph_3_SiH (**6**), Ph_2_MeSiH (**10**)) were also recovered in mostly intact form (Table 4, entries 4, 5). The results here indicate that the added ions and the physiological pH have no noticeable effect on the stability of the Si—H bonds of dihydro‐ and monohydrosilanes. However, the added ions do accelerate the reaction of the Si—H bonds in monoalkylsilanes.

## 3 Conclusion

**Figure 4:**
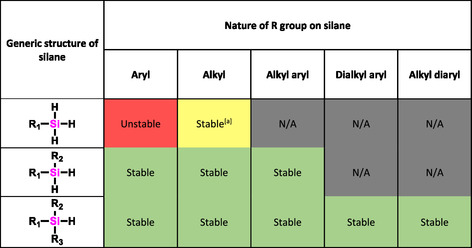
Hydrolytic stability of hydrosilanes categorized by number of hydrogens and nature of R groups (alkyl vs. aryl). The stability designations in this table are made based on the combined results of: reactivity in aqueous mixtures monitored in situ using ^1^H–NMR (Study 1), reactivity in water evaluated using ^1^H–NMR (Studies 2 and 3), reactivity in water evaluated using GC‐MS (Study 4), and reactivity in buffered aqueous solutions evaluated using ^1^H–NMR (Study 5). [a] Hydrolyzed in the presence of salts/ions.

We have investigated how the number of hydrogens on silicon (mono‐, di‐, trihydro) and the nature of the substituents on silicon (aryl, alkyl) affect the aqueous stability of hydrosilanes. We probed this question in five distinct experiments using both ^1^H–NMR and GC‐MS as analytical tools. The results from these experiments are summarized in **Figure** **4** and show that while trihydrosilanes—especially if the substituent on silicon is an aryl group—are susceptible to spontaneous hydrolysis, dihydro‐ and monohydrosilanes are generally stable in neutral aqueous conditions regardless of the substituents on silicon. General reactivity trends are consistent across all five studies, including triplicate reproduction in study 4. The results of this study portend the relevance of hydrosilanes as bioactive molecules. The studies were conducted at neutral pH, as the pH of biological systems is typically neutral. We note that it may be necessary to consider stability in either acidic or basic aqueous conditions, particularly when considering potential methods of drug administration (e.g., exposure to the highly acidic stomach when orally ingested) or for application in aberrant biological environments; such studies are outside the scope of this work. This compiled articulation of reactivity will help inform future organosilane design, in particular for their application as bioisosteres of carbon in bioapplied molecules.

## 4 Experimental Section

4.1

4.1.1

##### General Information

^1^H, ^13^C, and ^29^Si NMR were recorded using a 400 MHz Bruker multiprobe NMR instrument. The GC‐MS system was an Agilent 5977C GC/MSD with an electron impact (EI) ionization technique. The inlet was set to 250 °C, and the injection volume was (0.5 μL). The carrier gas was all GC‐MS runs was He.

^1^H–NMR time‐lapse experiments were run using deuterated solvents and referenced to MeOD‐d_4_ at 3.31 ppm. Residual water signal in MeOD‐d_4_ was observed at 4.8 and 4.6 (s) ppm. A control run using Ph_3_SiH in 100% MeOD‐d_4_ confirmed no reaction with the solvent. MeOD‐d_4_ was purchased from Sigma–Aldrich and used as received. D_2_O was purchased from Sigma–Aldrich and used as received. The silanes used were commercially available and used as received. Phenylsilane and dimethylphenylsilane were purchased from TCI and used as received. Dodecylsilane was purchased from Gelest and used as received. Triphenylsilane, triethylsilane, diethylsilane, and chloro(methyl)diphenylsilane were purchased from Sigma–Aldrich and used as received. Diphenylsilane and triisopropylsilane were purchased from Alfa Aesar and used as received. Diphenylmethylsilane was prepared by reduction of chloro(methyl)diphenylsilane using NaBH_4_ as reported in the literature.^[^ ^29^ ^]^ Aqueous extraction was performed using EtOAc. PBS tablets were used to prepare a pH 7.4 solution. All reactions were carried out without special precautions to exclude air/moisture unless otherwise specified.

##### Reactivity in Aqueous Mixtures Monitored In Situ using ^1^H–NMR (Study 1)

To an NMR tube was added the silane (≈5 mg), and then MeOD‐d_4_ (0.9 mL) or DMSO‐d_6_. After sonication for 30 s, D_2_O (0.1 mL) was added, and the NMR tube was shaken, followed immediately by ^1^H–NMR acquisitions at set intervals: 0, 10, 60 min, and 24 h.

##### Reactivity in Water Evaluated using ^1^H–NMR after Liquid/Liquid Extraction (Study 2 & 5)

To a flame‐dried flask under nitrogen was added the silane (0.25 mmol for study 2 or 1 mmol for study 5), followed by distilled H_2_O (2.5 mL, 0.1 M) or PBS buffer solution (10 mL, 0.1 M, pH 7.4). The reaction was stirred under nitrogen for 1 h, and then extracted using 5 × 5 mL EtOAc. The combined organic fraction was dried over MgSO_4_, then concentrated under vacuum. The percent recovery was calculated based on the mass remaining after drying. The sample was dissolved in the appropriate deuterated solvent for ^1^H–, ^13^C–, and ^29^Si–NMR acquisition. To minimize evaporative loss of material, a modified extraction was used for the volatile hydrosilanes (phenylsilane, diethylsilane, triisopropylsilane, triethylsilane, and dimethylphenylsilane): the reaction was cooled to 0 °C, MgSO_4_ powder was added, followed by 1 eq. of mesitylene internal standard and CDCl_3_. The entire mixture was sonicated for 3 min and filtered into an NMR tube for NMR analysis.

##### Reactivity in Water Evaluated using ^1^H–NMR after Direct Removal of Solvent in Vacuo (Study 3)

To a flame‐dried flask under nitrogen was added silane (0.25 mmol) followed by distilled H_2_O (1 mL, 0.25 M). The reaction was stirred under nitrogen for 1 h and then concentrated under vacuum to remove H_2_O. The sample was reconstituted in the appropriate deuterated solvent for NMR acquisition.

##### Reactivity in Water Evaluated using GC‐MS (Study 4)

Hydrosilane (0.5 mmol) was added to a vial, followed by 5 mL of distilled water, and then stirred for 1 h. The reaction was transferred to a separatory funnel and extracted with 25 mL of EtOAc. An aliquot (1.5 mL) of this organic layer was then transferred to a GC vial, and then 0.5 μL was injected (via autosampler) into the GC‐MS to determine the amount of leftover hydrosilane. This was repeated three times, and the average response was used to calculate the concentration of hydrosilane in the organic layer.

## Conflict of Interest

The authors declare no conflict of interest.

## Supporting information

Supplementary Material

## Data Availability

The data that support the findings of this study are available in the supplementary material of this article.
