# Supplementary material for: Evaluating the Aqueous Stability of Alkyl‐/Aryl‐Hydrosilanes by NMR Spectroscopy and GC‐MS
Source: Chempluschem. 2025 Jul 21;90(10):e202500310. doi: 10.1002/cplu.202500310 (PMC12509473; doi:10.1002/cplu.202500310)
Supplement: Supplementary file 1 — Supplementary Material [file CPLU-90-e202500310-s001.pdf]

Contents

General information 1
Reactivity in aqueous mixtures monitored *in situ* using ^{1}H-NMR (Study 1) 2
Reactivity in water evaluated using ^{1}H-NMR after liquid/liquid extraction (Study 2) 23
Reactivity in water evaluated using ^{1}H-NMR after direct removal of solvent *in vacuo* (Study 3) 33
Reactivity in water evaluated using GCMS (Study 4) 40
Reactivity in buffered aqueous solutions evaluated using ^{1}H-NMR (Study 5) 51
MSDS information on moisture sensitivity of hydrosilanes 55

General information

^{1}H, ^{13}C, and ^{29}Si NMR were recorded using a 400 MHz Bruker multi-probe NMR instrument. The GCMS system was Agilent 5977C GC/MSD with an electron impact (EI) ionization technique. The inlet was set to 250 °C, and the injection volume was (0.5 µL). The carrier gas was all GC-MS runs was He.

^{1}H-NMR time-lapse experiments were run using deuterated solvents and referenced to MeOD-d_4 at 3.31 ppm. Residual water signal in MeOD-d_4 was observed at 4.8 (s) ppm and 4.6 (s) ppm. A control run using Ph_3SiH in 100% MeOD-d_4 confirmed no reaction with the solvent. MeOD-d_4 was purchased from Sigma-Aldrich and used as received. D_2O was purchased from Sigma-Aldrich and used as received. The silanes used were commercially available and used as received. Phenylsilane and dimethylphenylsilane were purchased from TCI and used as received. Dodecylsilane was purchased from Gelest and used as received. Triphenylsilane, triethylsilane, diethylsilane, and chloro(methyl)diphenylsilane were purchased from Sigma-Aldrich and used as received. Diphenylsilane and triisopropylsilane were purchased from Alfa Aesar and used as received. Diphenylmethylsilane was prepared by reduction of chloro(methyl)diphenylsilane using $NaBH_4$ as reported in the literature. Aqueous extraction was performed using ethyl acetate (EtOAc). Phosphate-buffered saline (PBS) tablets were used to prepare a pH 7.4 solution. All reactions were carried out without special precaution to exclude air/moisture unless specified otherwise.

Reactivity in aqueous mixtures monitored *in situ* using ^{1}H-NMR (Study 1)

Experimental

To an NMR tube was added the silane (~5 mg) and then MeOD-d_4 (0.9 mL) or DMSO-d_6. After sonication for 30 seconds, D_2O (0.1 mL) was added and the NMR tube was shaken followed immediately by ^{1}H-NMR acquisitions at set intervals: 0 min, 10 mins, 60 mins, and 24 hours.

The following steps* were used to quantify the amount (%) of hydrosilane leftover after 24 hours in the NMR solvent system:

1- Integrate the Si-H signal and calibrate it to the appropriate number of proton(s), at 0 minutes
2- Integrate the residual MeOD-d_4 signal, at 0 minutes
3- Integrate the Si-H signal and the residual MeOD-d_4 signals at subsequent time points (10 minutes, 60 minutes, and 24 hours).
4- Calibrate the residual MeOD-d_4 integration to the value from step 2 for each time point.
5- Calculate the percent change in Si-H signal over 24 hours
    a. E.g. for phenylsilane **1** :
    $\frac{3-2.06}{3}$ x 100= 31% decrease in the Si-H signal relative to time zero.
    This is reported as 69% leftover hydrosilane.

*In the case of dodecylsilane **2**, the residual DMSO-d_6 signal was used.

*In the case of *i*-Pr_3SiH **8**, the Si-H signal overlaps with the residual MeOD-d_4 signal at 3.31 ppm. The combined Si-H and residual MeOD-d_4 signals were integrated along with the aliphatic protons at 1.07 ppm. No change was observed in their relative integration over a 24-hour period, therefore we reported that 100% of the hydrosilane was leftover.

*In the case of Ph_2MeSiH **10**, steps 1-5 were carried out as described. The calculations revealed that the leftover hydrosilane was 104%. The NMR spectra collected over the 24 hour was largely unchanged. Therefore, for Ph_2MeSiH **10**, we reported that 100% of the hydrosilane was leftover.

Phenylsilane **1**

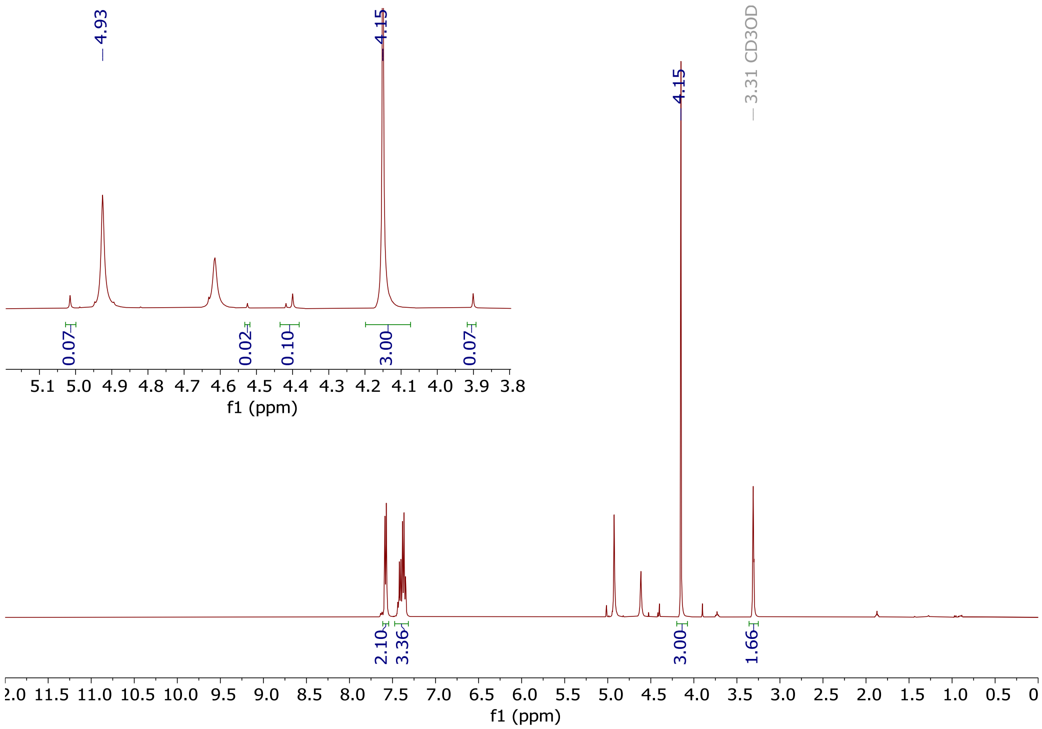

*Figure 1. ¹H-NMR of **1** in 10% D_2O:MeOD-d_4 at 0 minutes.*

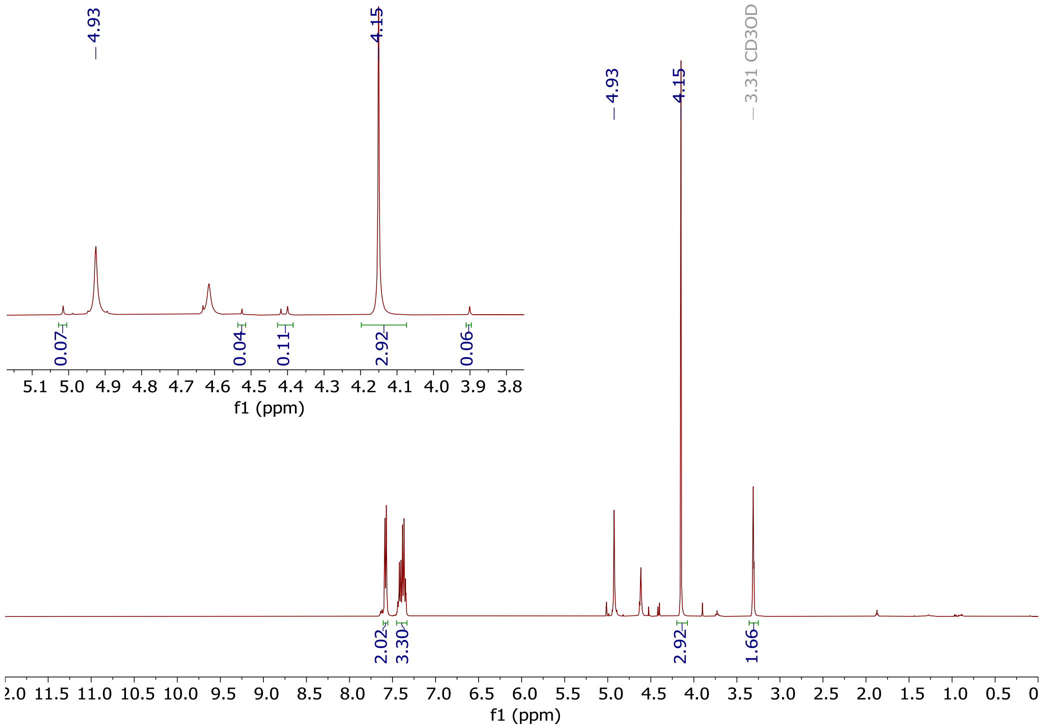

*Figure 2. ¹H-NMR of **1** in 10% D_2O:MeOD-d_4 at 10 minutes.*

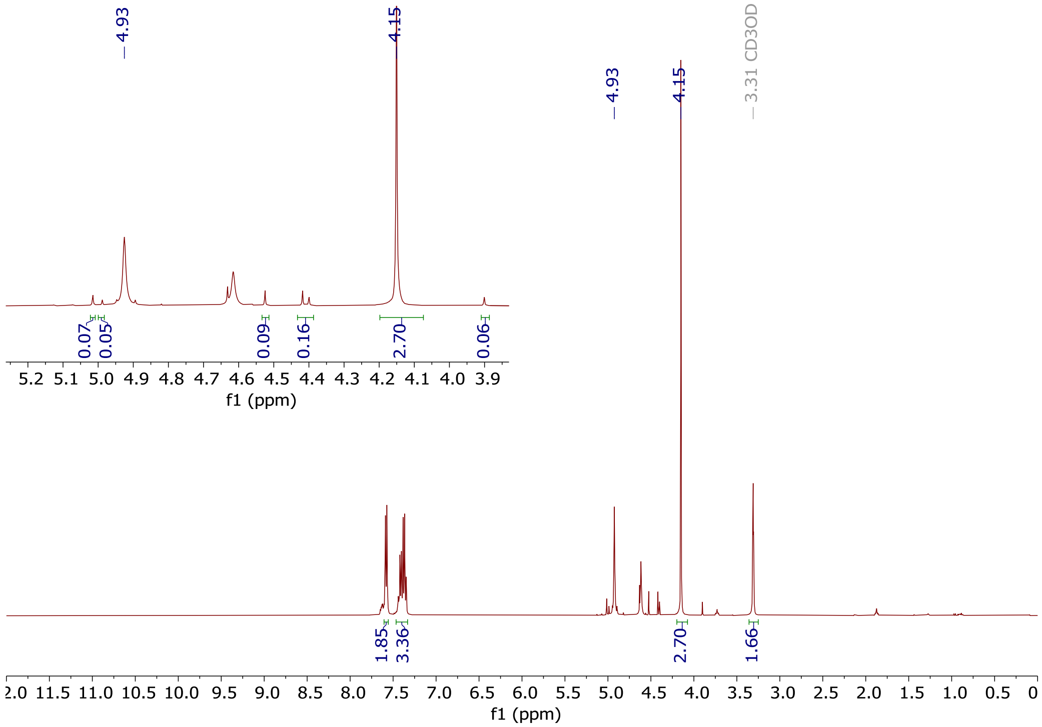

*Figure 3. ^{1}H-NMR of **1** in 10% D_2O:MeOD-d_4 at 60 minutes.*

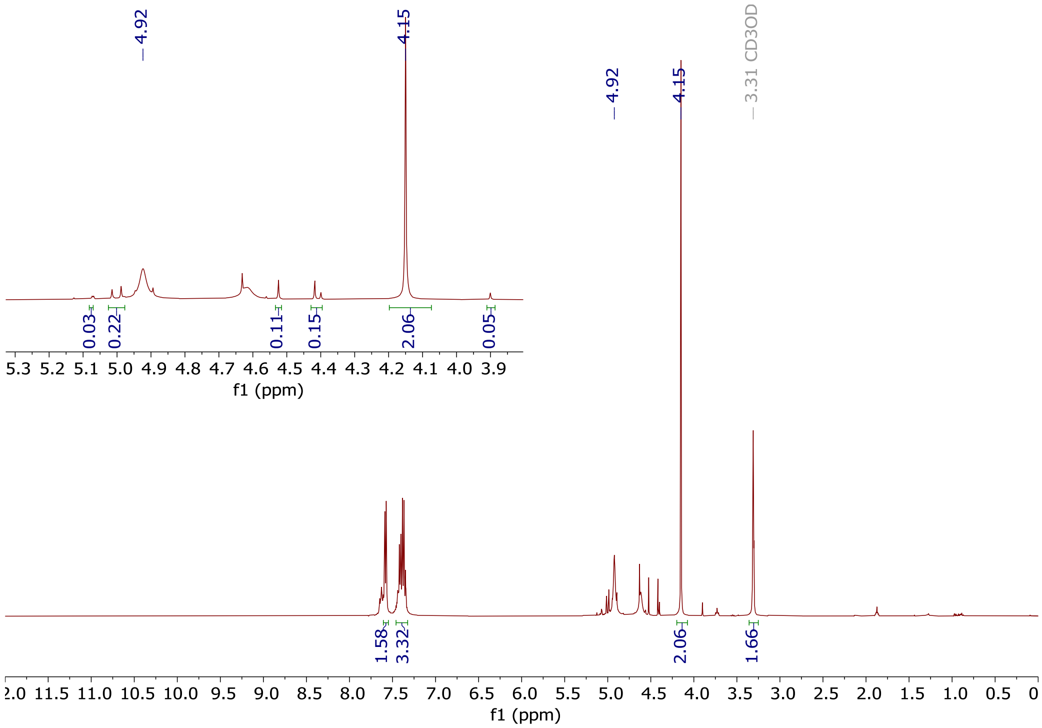

*Figure 4. ^{1}H-NMR of **1** in 10% D_2O:MeOD-d_4 at 24 hours.*

Dodecylsilane **2**

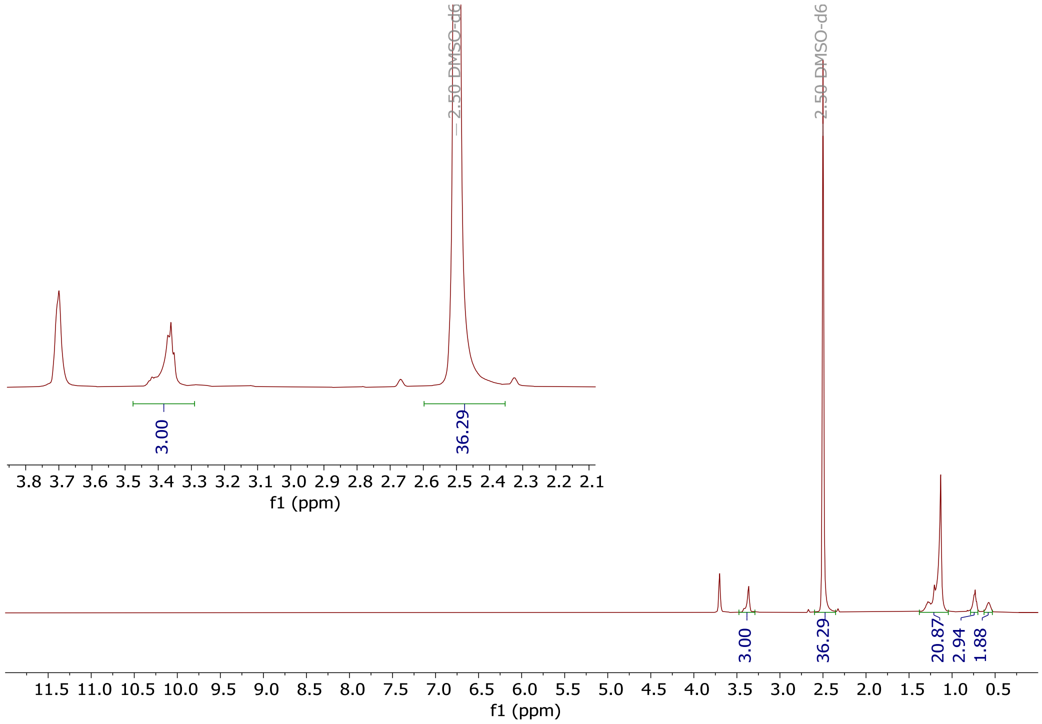

*Figure 5. ^{1}H-NMR of **2** in 10% D_2O:DMSO-d_6 at 0 minutes.*

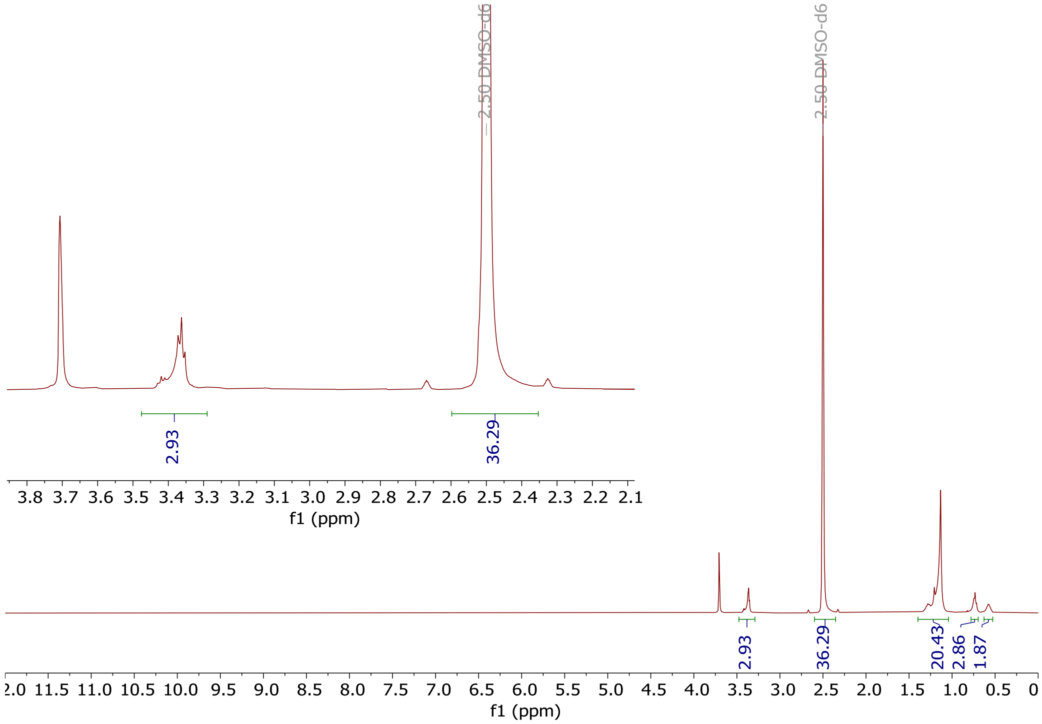

*Figure 6. ^{1}H-NMR of **2** in 10% D_2O:DMSO-d_6 at 10 minutes.*

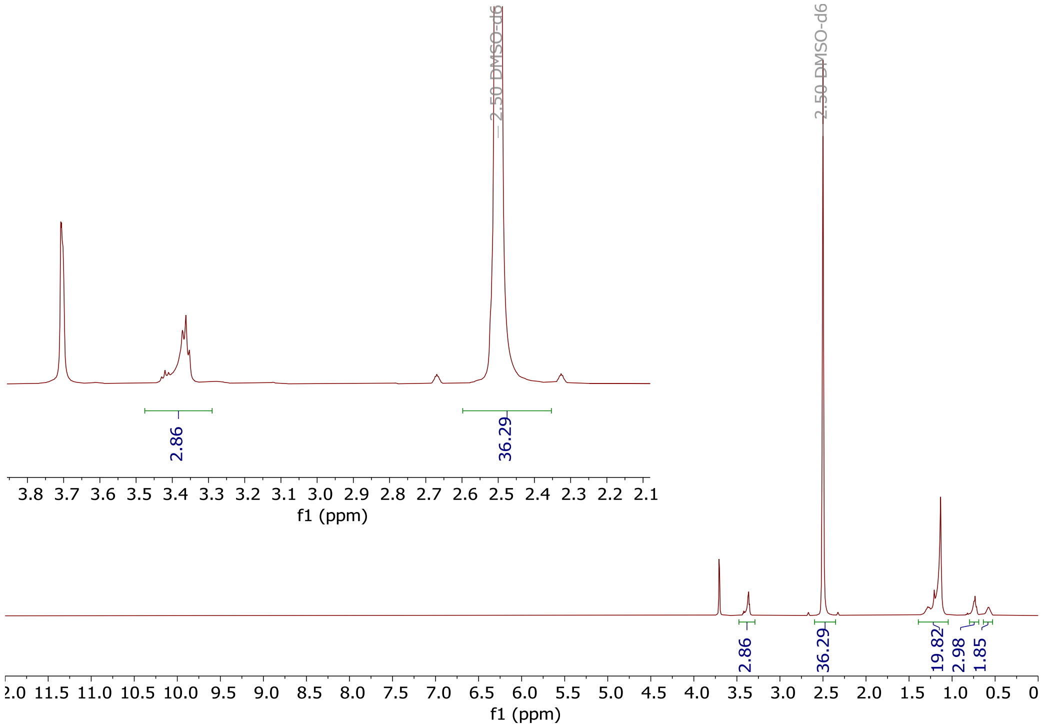

Figure 7. [1]*H-NMR of* ***2*** *in 10%* D_2O*:DMSO-*d_6 *at 60 minutes.*

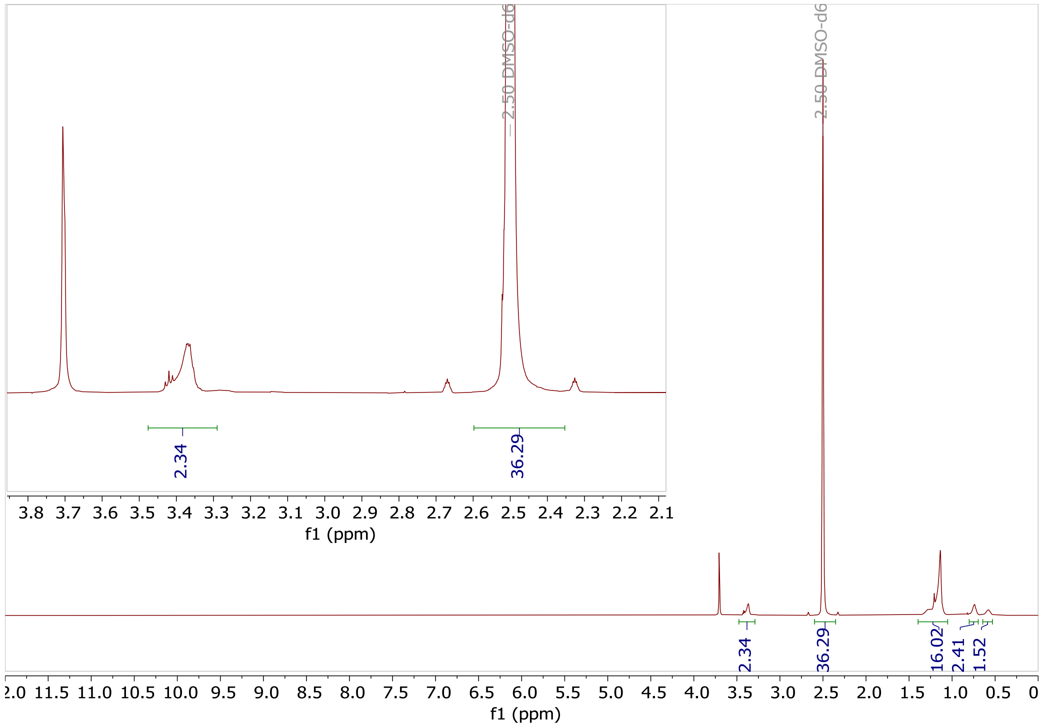

Figure 8. [1]*H-NMR of* ***2*** *in 10%* D_2O*:DMSO-*d_6 *at 24 hours.*

Diphenylsilane **3**

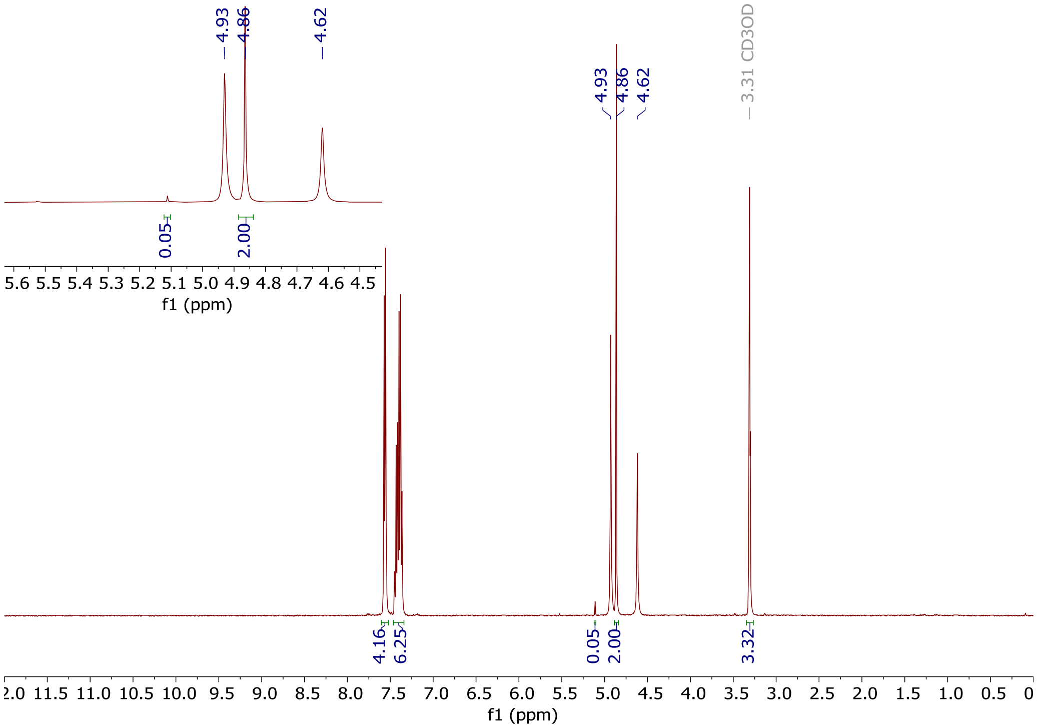

*Figure 9. ^{1}H-NMR of **3** in 10% D_2O:MeOD-d_4 at 0 minutes.*

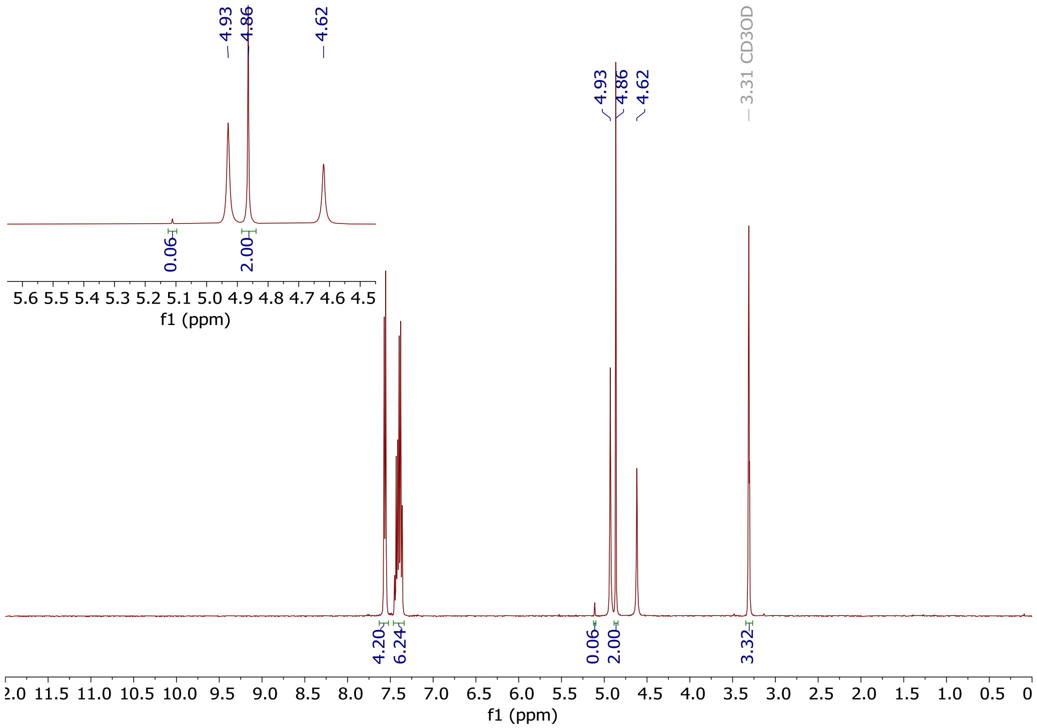

*Figure 10. ^{1}H-NMR of **3** in 10% D_2O:MeOD-d_4 at 10 minutes.*

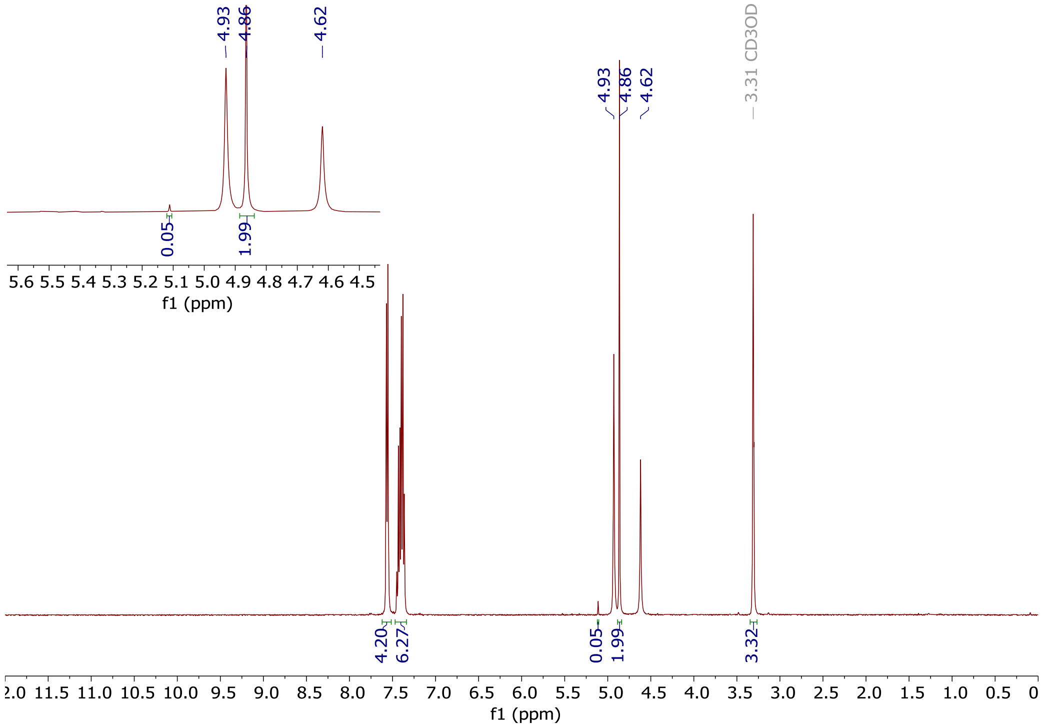

Figure 11. [1]*H-NMR of* ***3*** *in 10%* D_2O*:MeOD-*d_4 *at 60 minutes.*

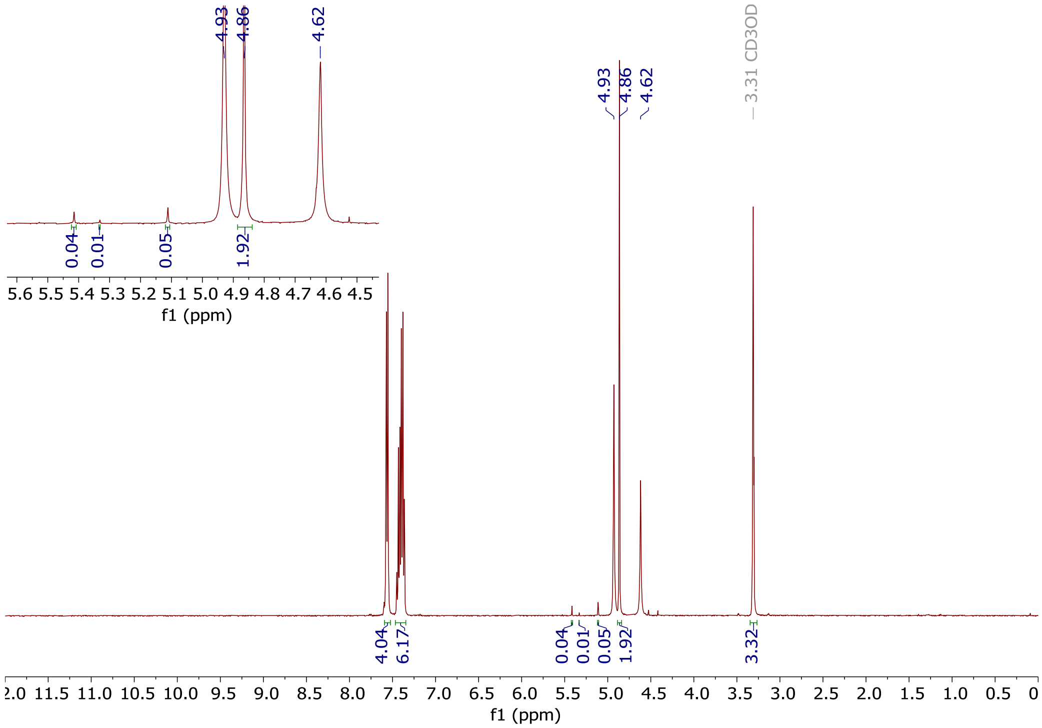

Figure 12. [1]*H-NMR of* ***3*** *in 10%* D_2O*:MeOD-*d_4 *at 24 hours.*

Diethylsilane **4**

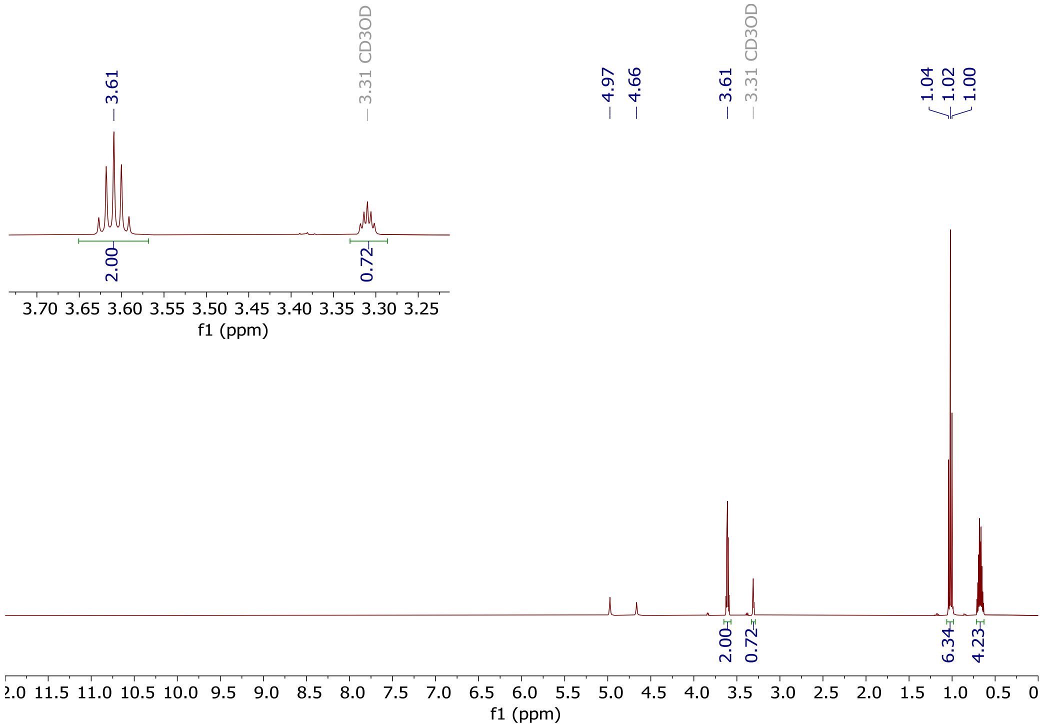

Figure 13. ^{1}H-NMR of **4** in 10% D_2O:MeOD-d_4 at 0 mins.

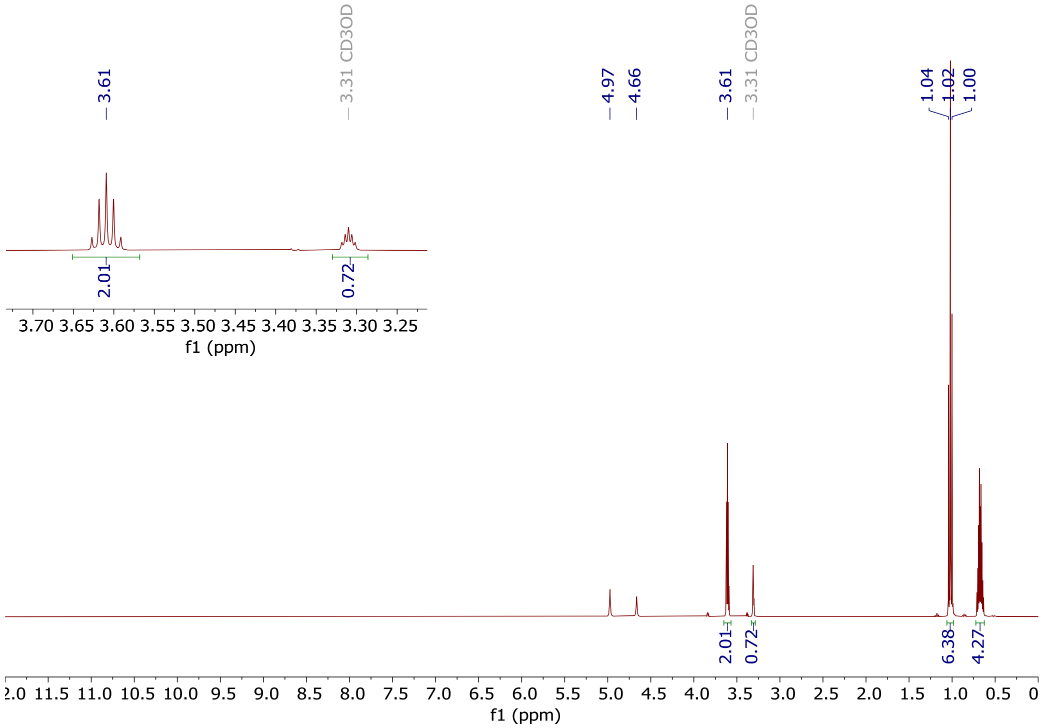

Figure 14. ^{1}H-NMR of **4** in 10% D_2O:MeOD-d_4 at 10 mins.

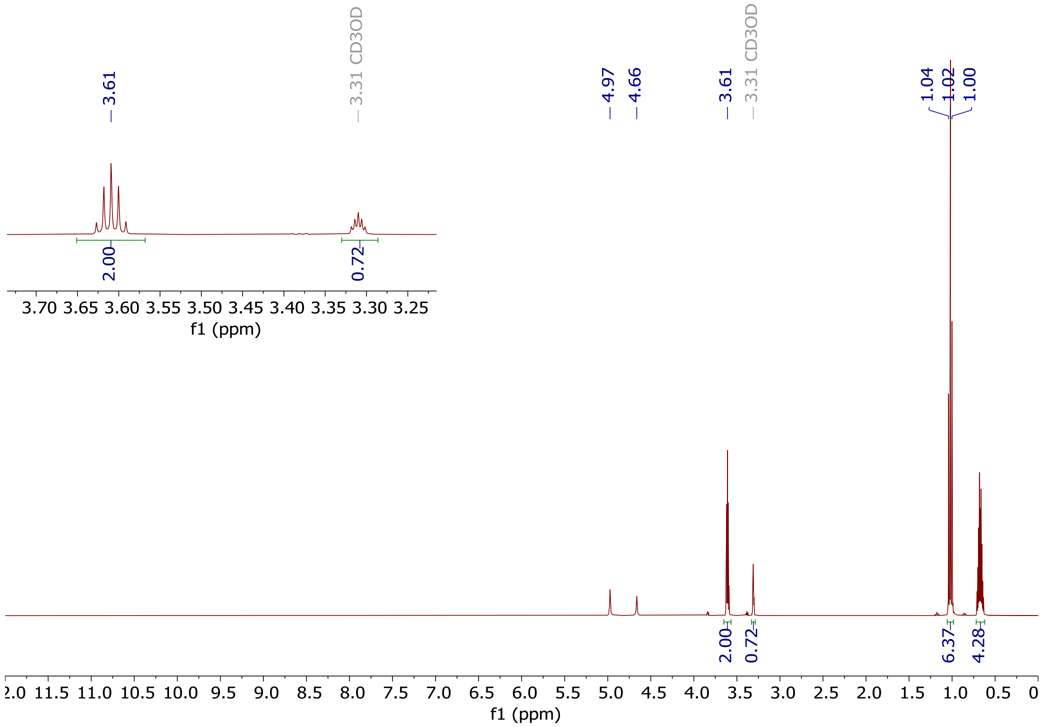

*Figure 15. ^{1}H-NMR of **4** in 10% D_2O:MeOD-d_4 at 60 mins.*

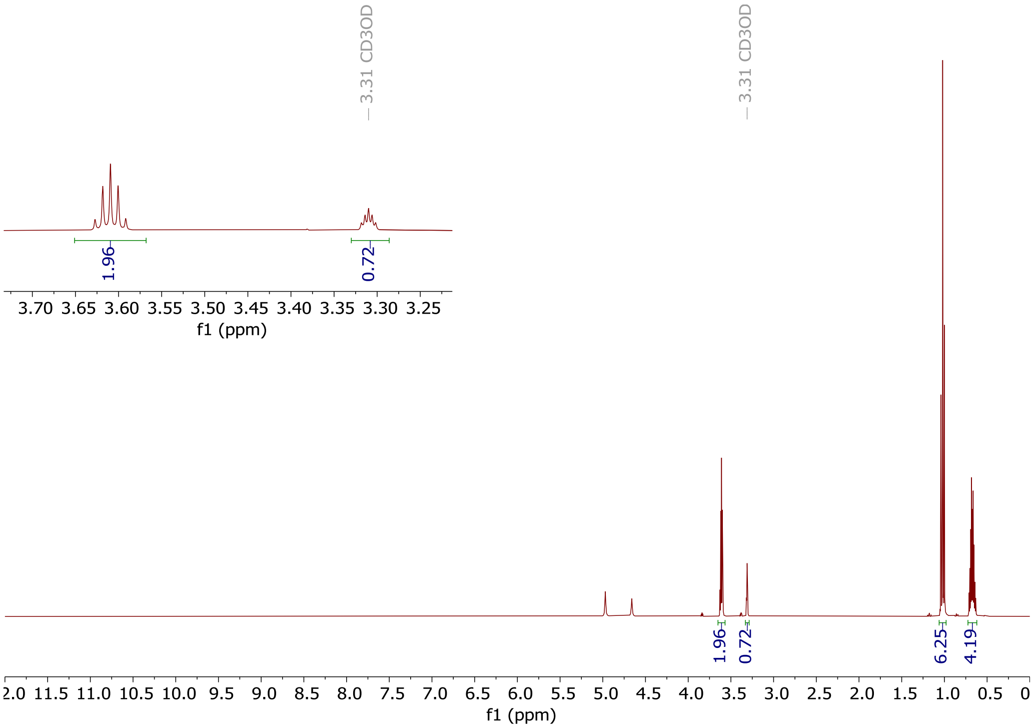

*Figure 16. ^{1}H-NMR of **4** in 10% D_2O:MeOD-d_4 at 24 hours.*

n-Hexylphenylsilane **5**

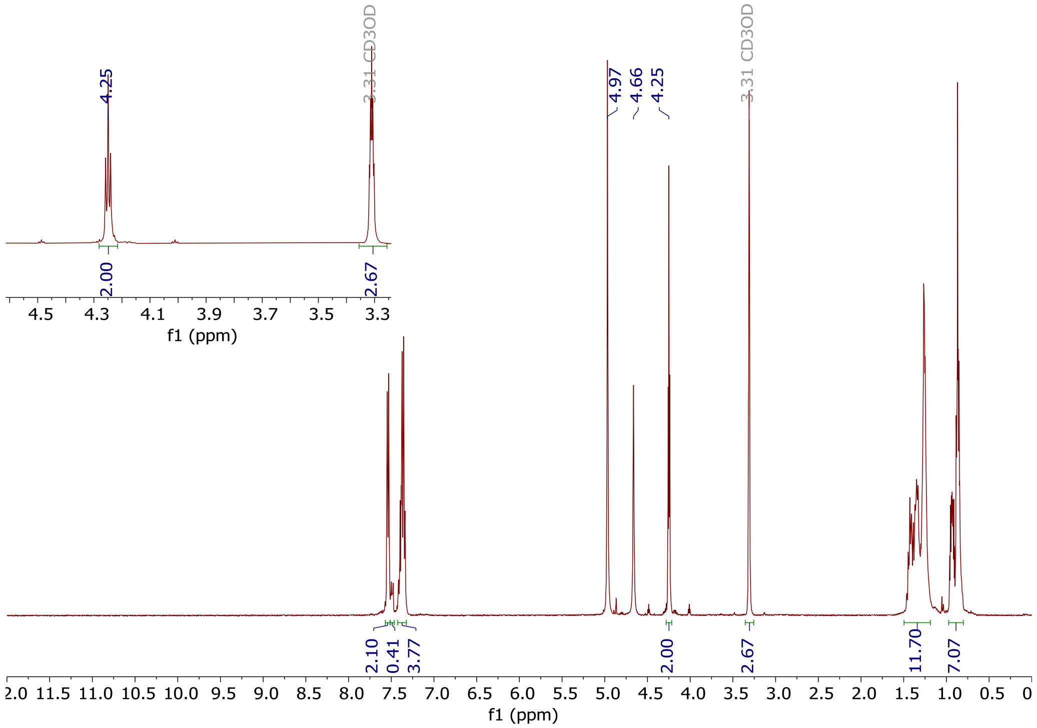

*Figure 17. ^{1}H-NMR of **5** in 10% D_2O:MeOD-d_4 at 0 minutes.*

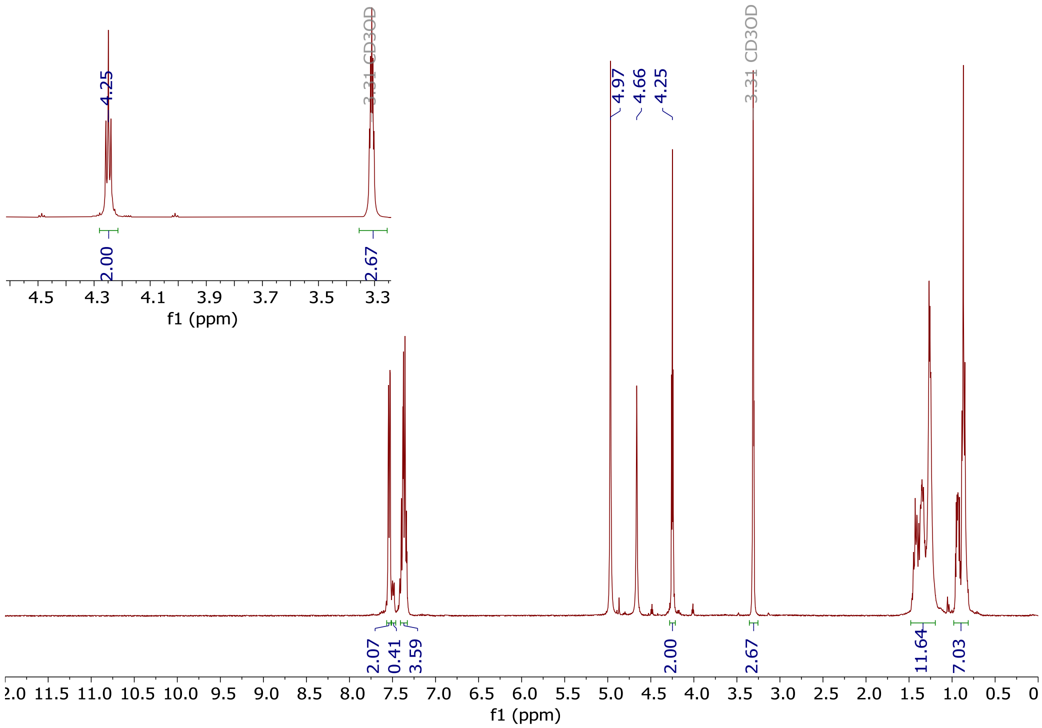

*Figure 18. ^{1}H-NMR of **5** in 10% D_2O:MeOD-d_4 at 10 minutes.*

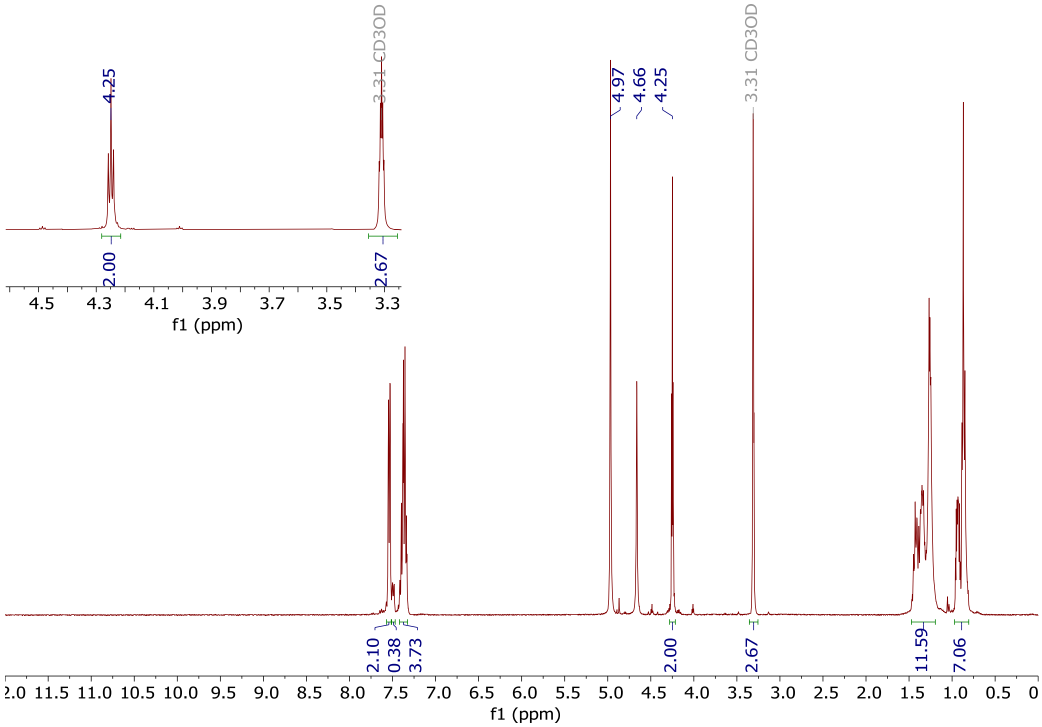

*Figure 19. ^{1}H-NMR of **5** in 10% D_2O:MeOD-d_4 at 60 minutes.*

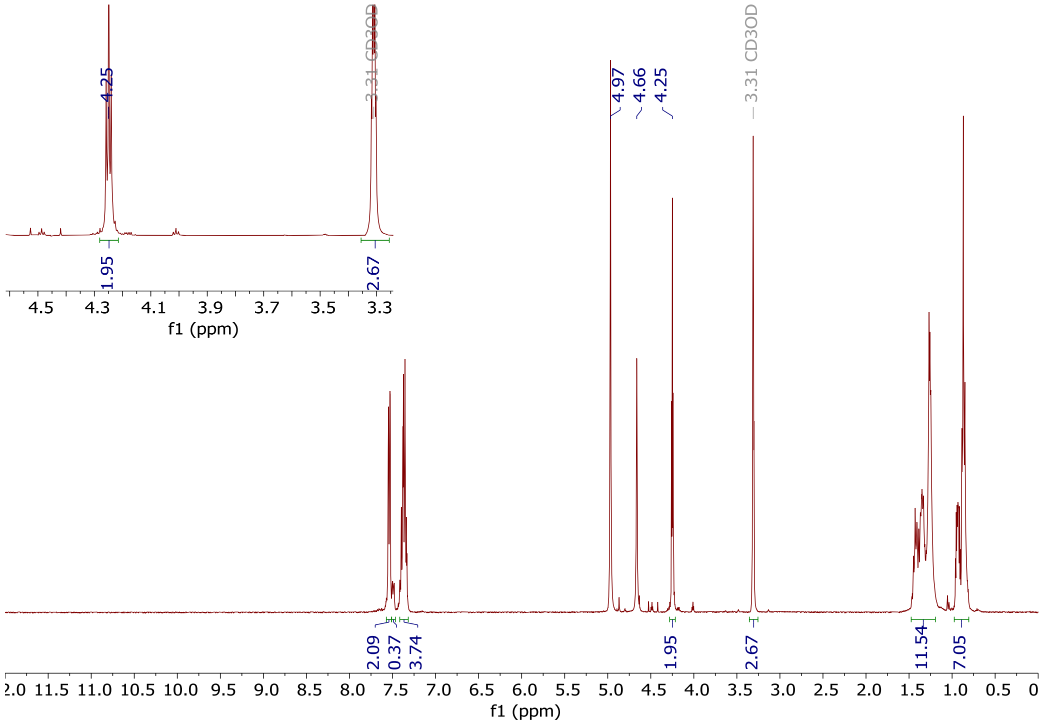

*Figure 20. ^{1}H-NMR of **5** in 10% D_2O:MeOD-d_4 at 24 hours.*

Triphenylsilane **6**

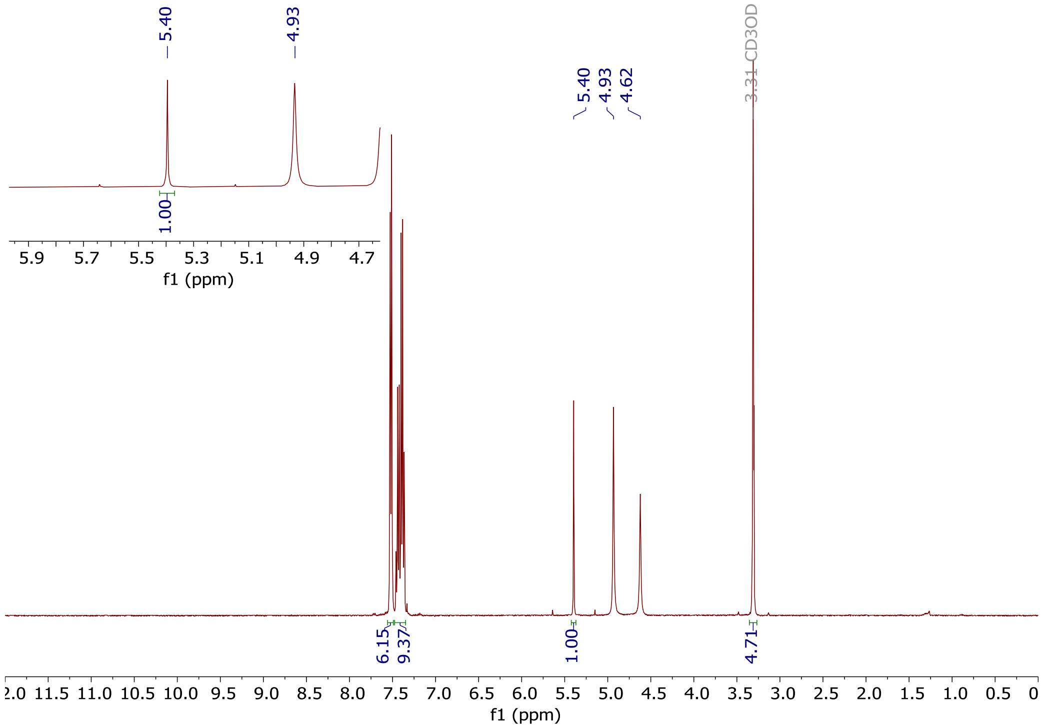

*Figure 21. ^{1}H-NMR of **6** in 10% D_2O:MeOD-d_4 at 0 minutes.*

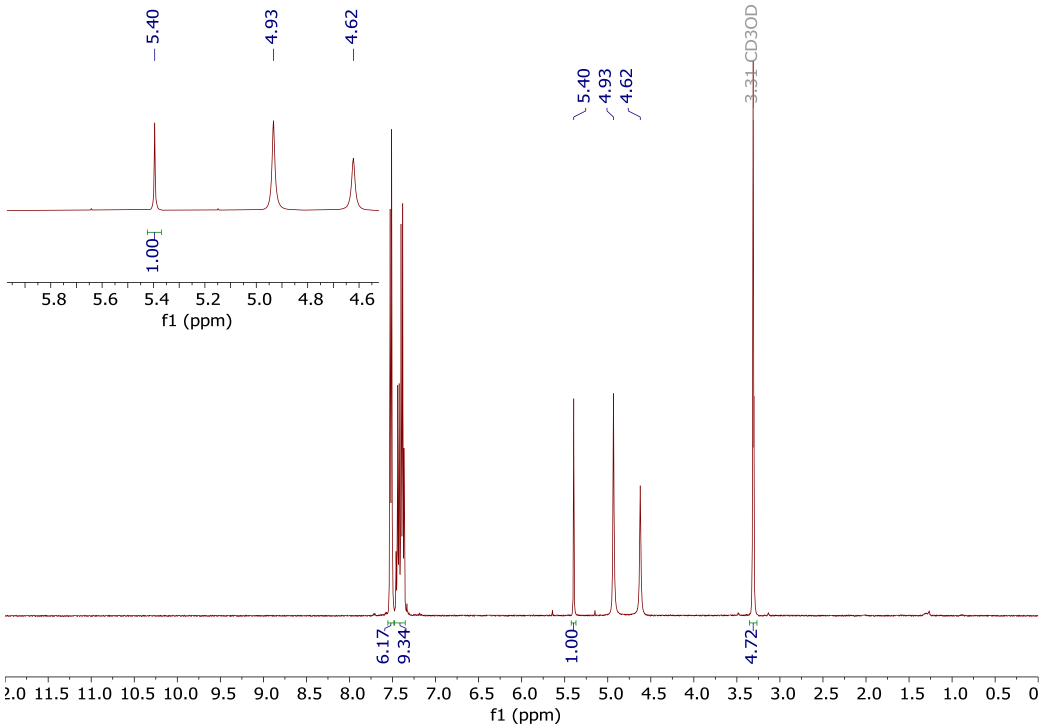

*Figure 22. ^{1}H-NMR of **6** in 10% D_2O:MeOD-d_4 at 10 minutes.*

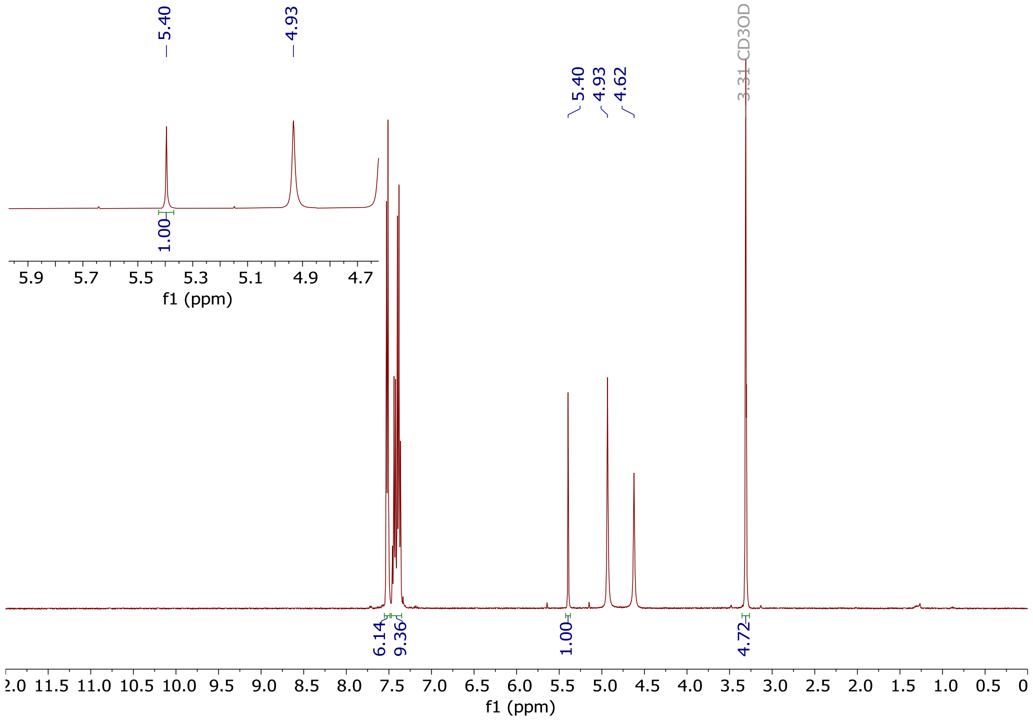

*Figure 23. ^{1}H-NMR of **6** in 10% D_2O:MeOD-d_4 at 60 minutes.*

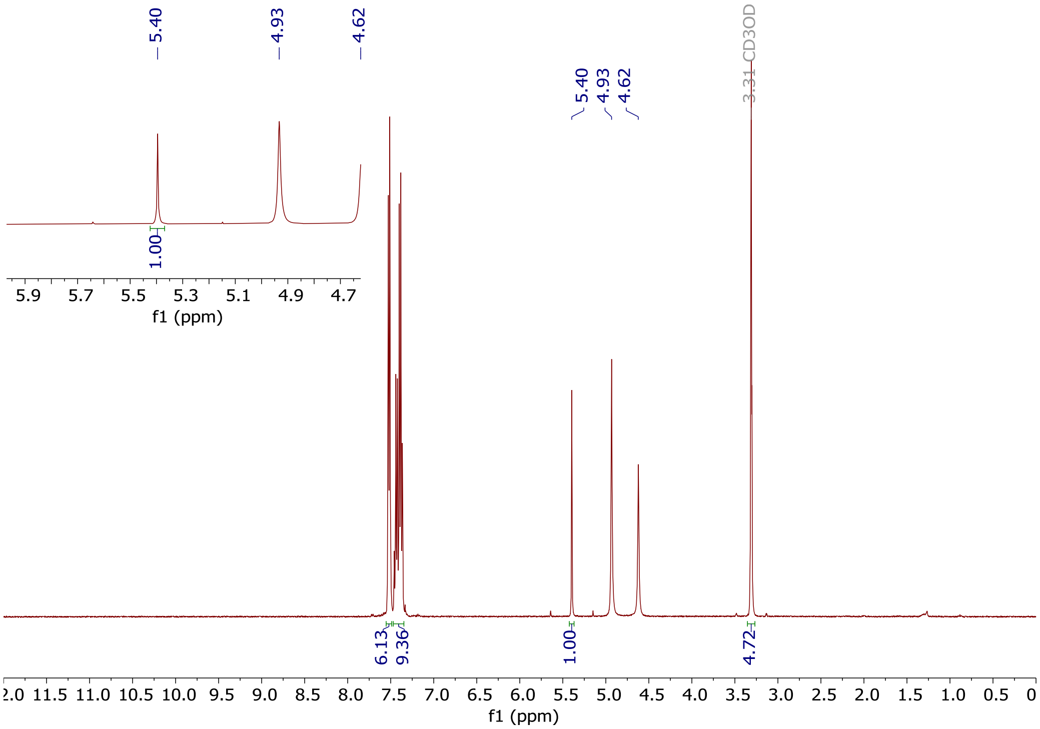

*Figure 24. ^{1}H-NMR of **6** in 10% D_2O:MeOD-d_4 at 24 hours.*

Triethylsilane **7**

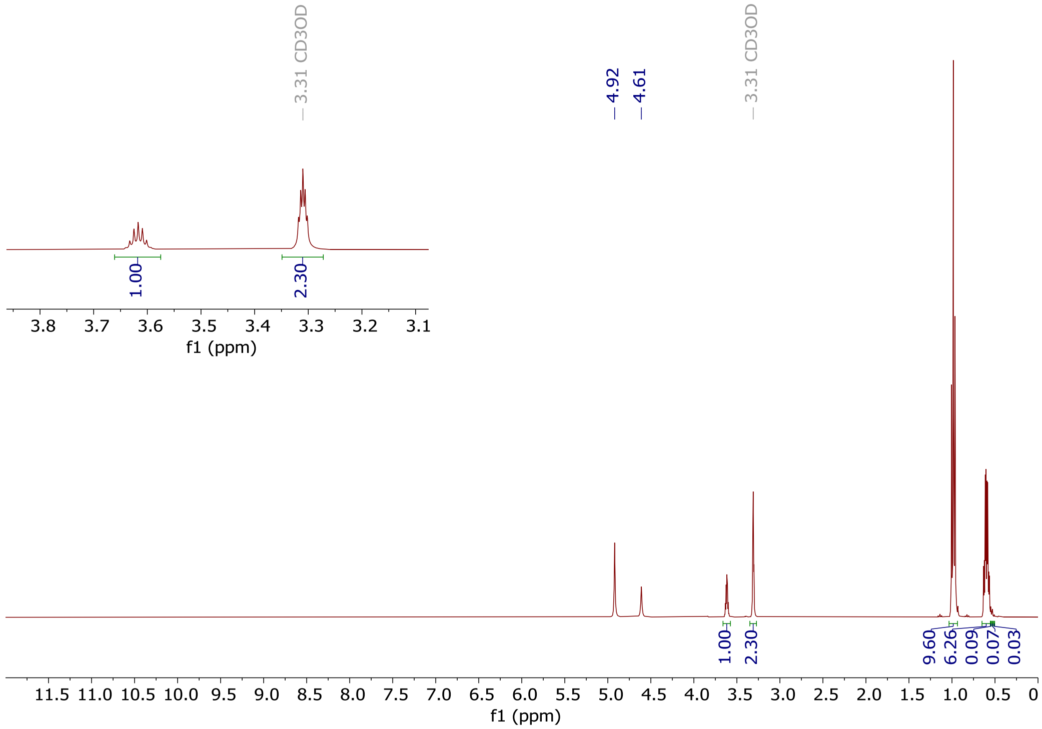

*Figure 25. ^{1}H-NMR of **7** in 10% D_2O:MeOD-d_4 at 0 minutes.*

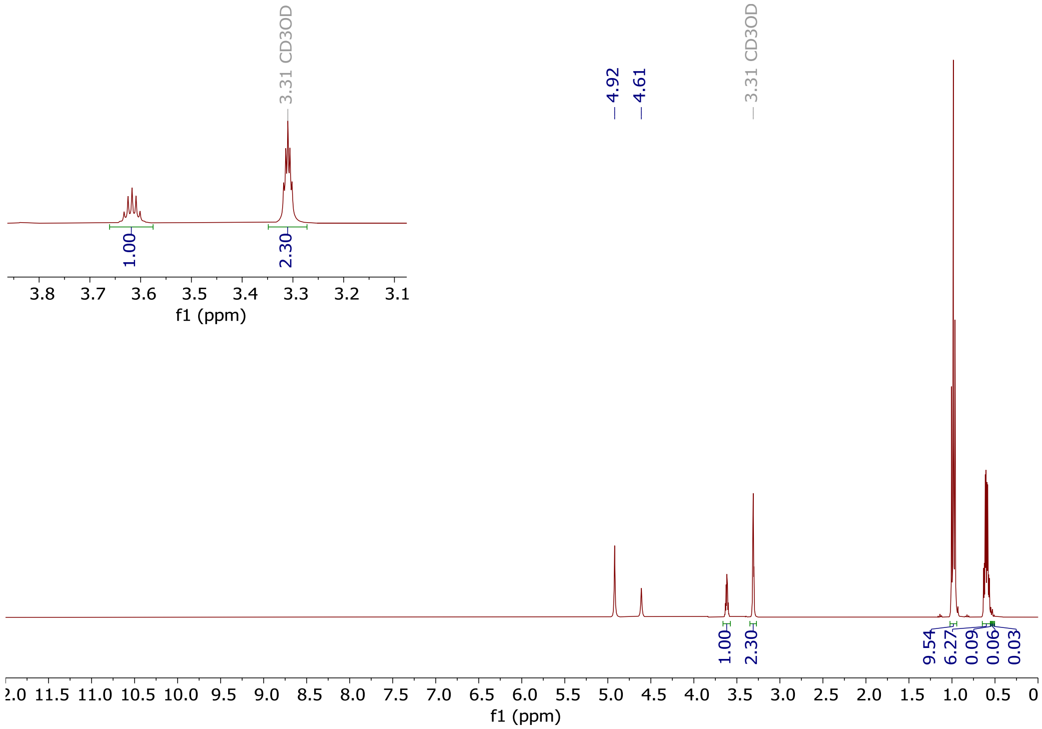

*Figure 26. ^{1}H-NMR of **7** in 10% D_2O:MeOD-d_4 at 10 minutes.*

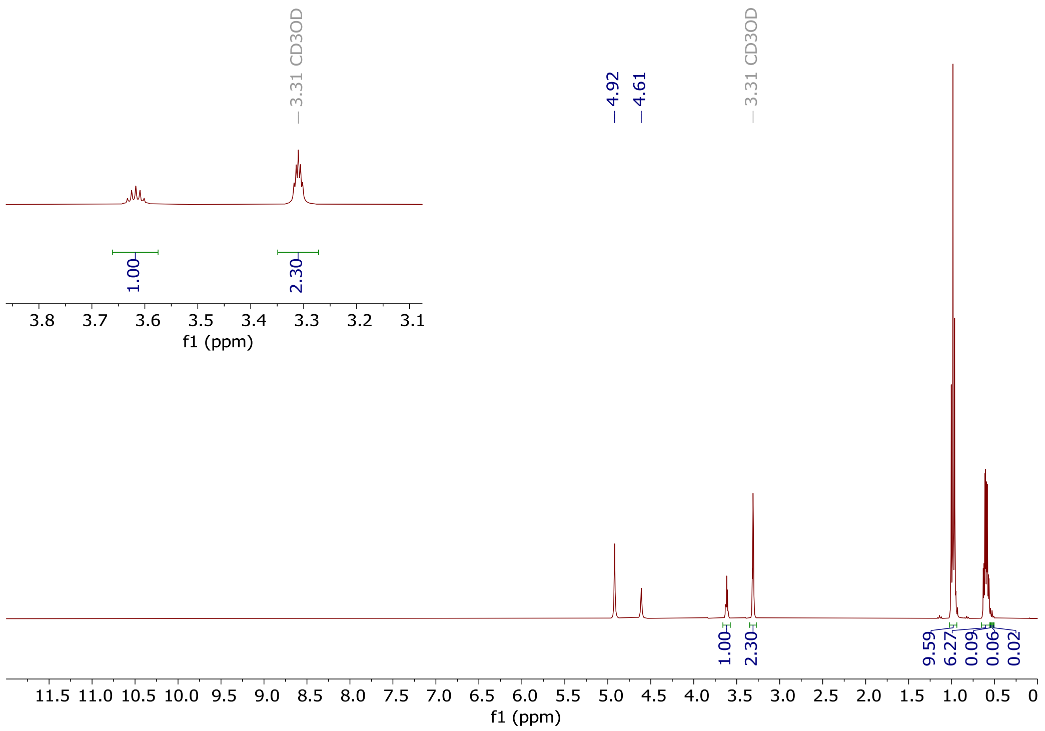

Figure 27. ^{1}*H-NMR of* ***7*** *in 10%* D_2O*:MeOD-*d_4 *at 60 minutes.*

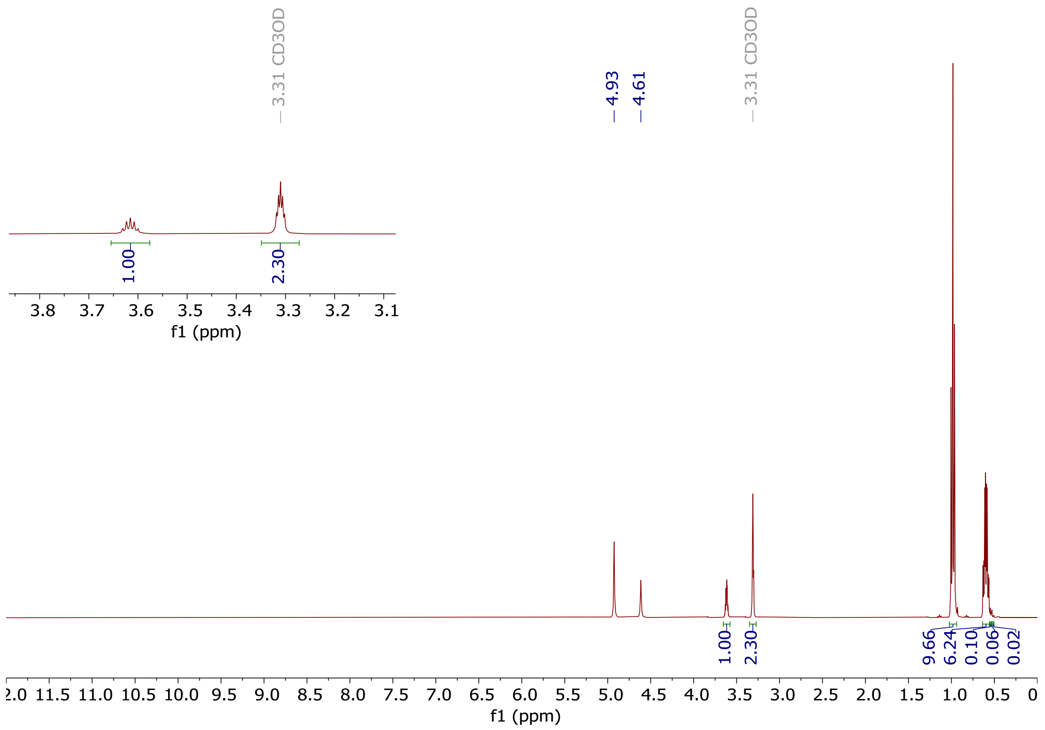

Figure 28. ^{1}*H-NMR of* ***7*** *in 10%* D_2O*:MeOD-*d_4 *at 24 hours.*

Triisopropylsilane **8**

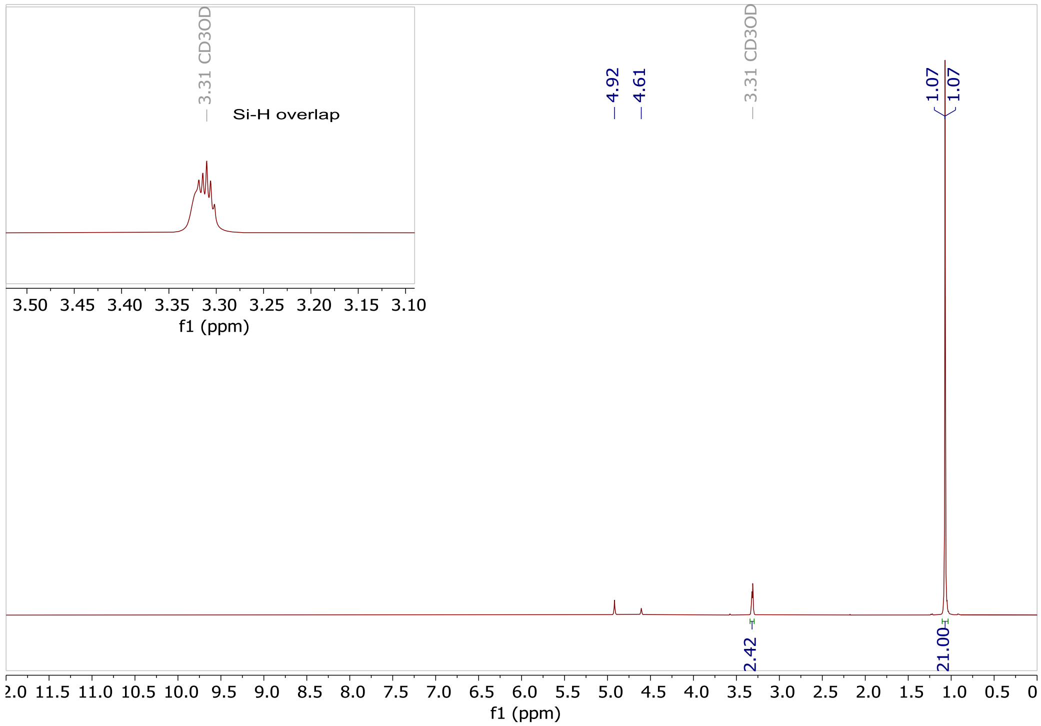

*Figure 29. ^{1}H-NMR of **8** in 10% D_2O:MeOD-d_4 at 0 minutes.*

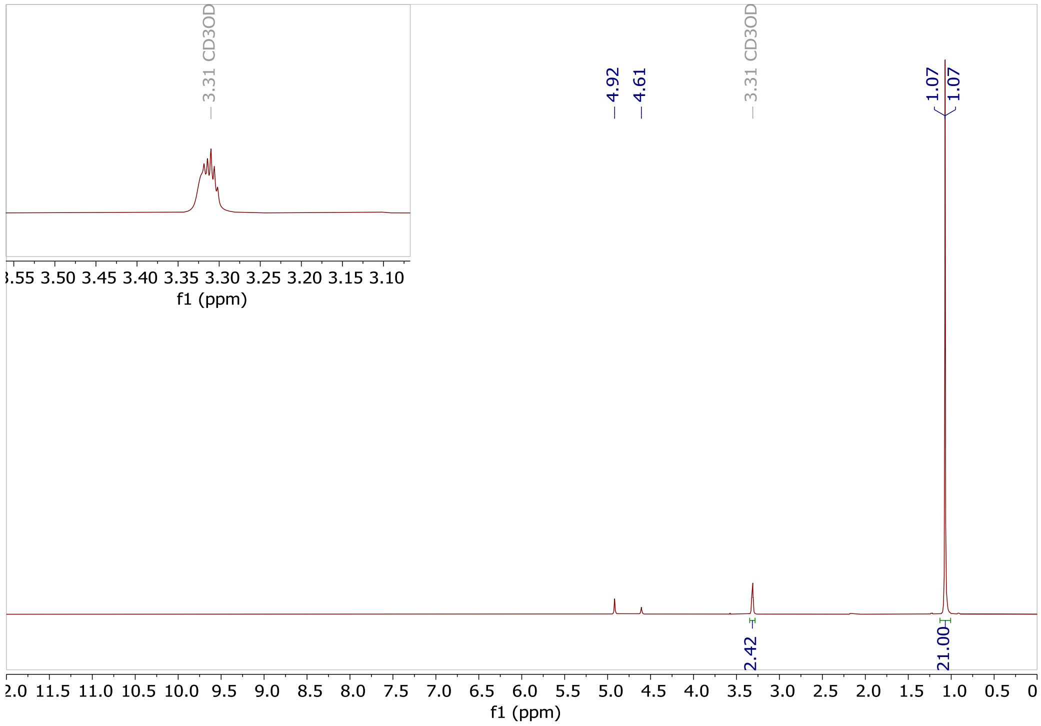

*Figure 30. ^{1}H-NMR of **8** in 10% D_2O:MeOD-d_4 at 10 minutes.*

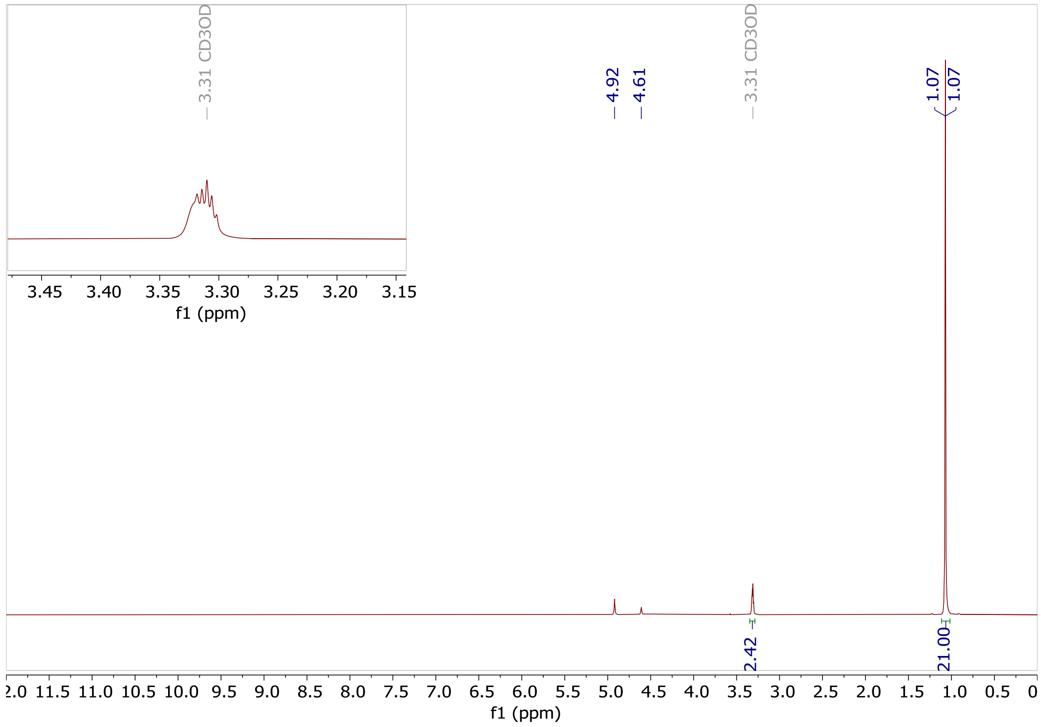

*Figure 31. ^{1}H-NMR of **8** in 10% D_2O:MeOD-d_4 at 60 minutes.*

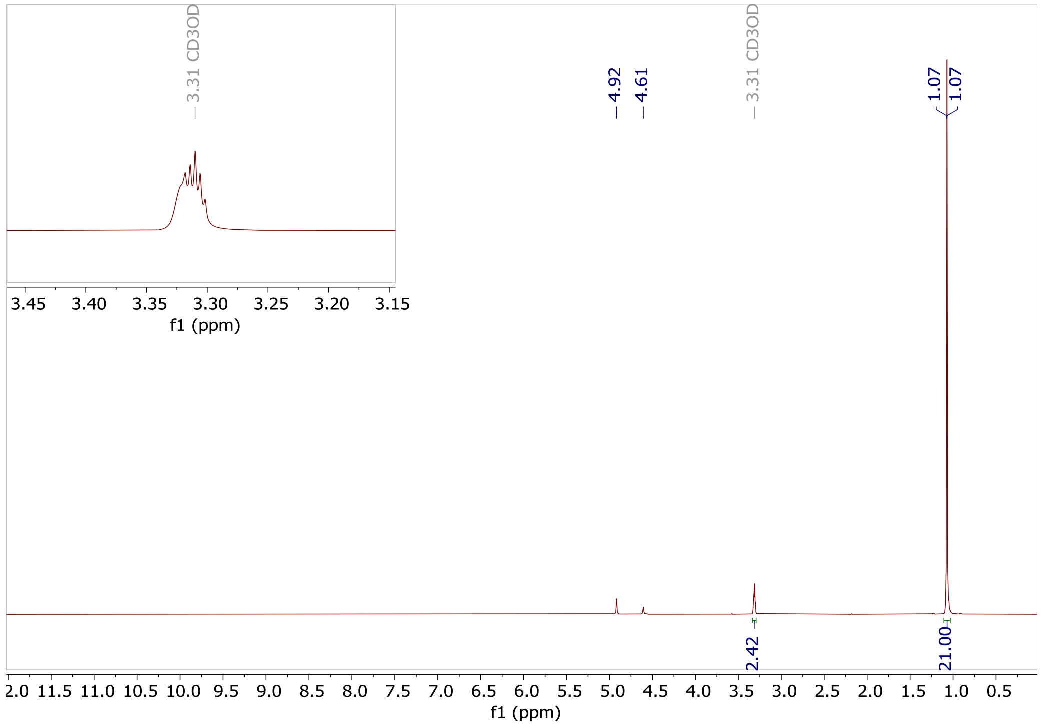

*Figure 32. ^{1}H-NMR of **8** in 10% D_2O:MeOD-d_4 at 24 hours.*

Dimethylphenylsilane **9**

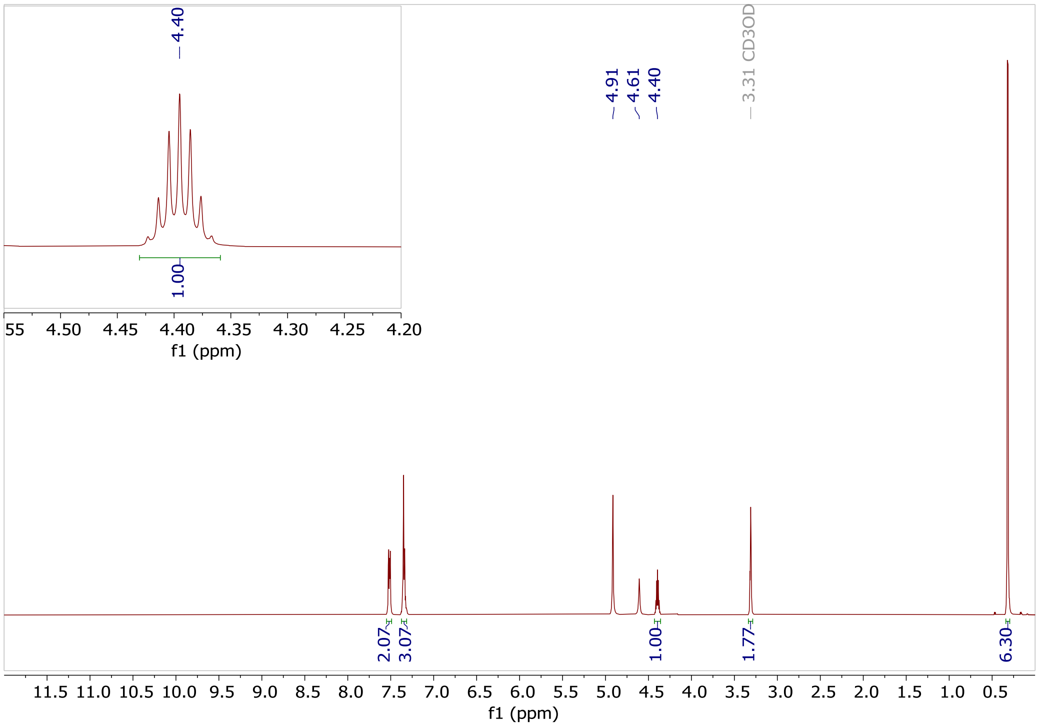

*Figure 33. ^{1}H-NMR of **9** in 10% D_2O:MeOD-d_4 at 0 minutes.*

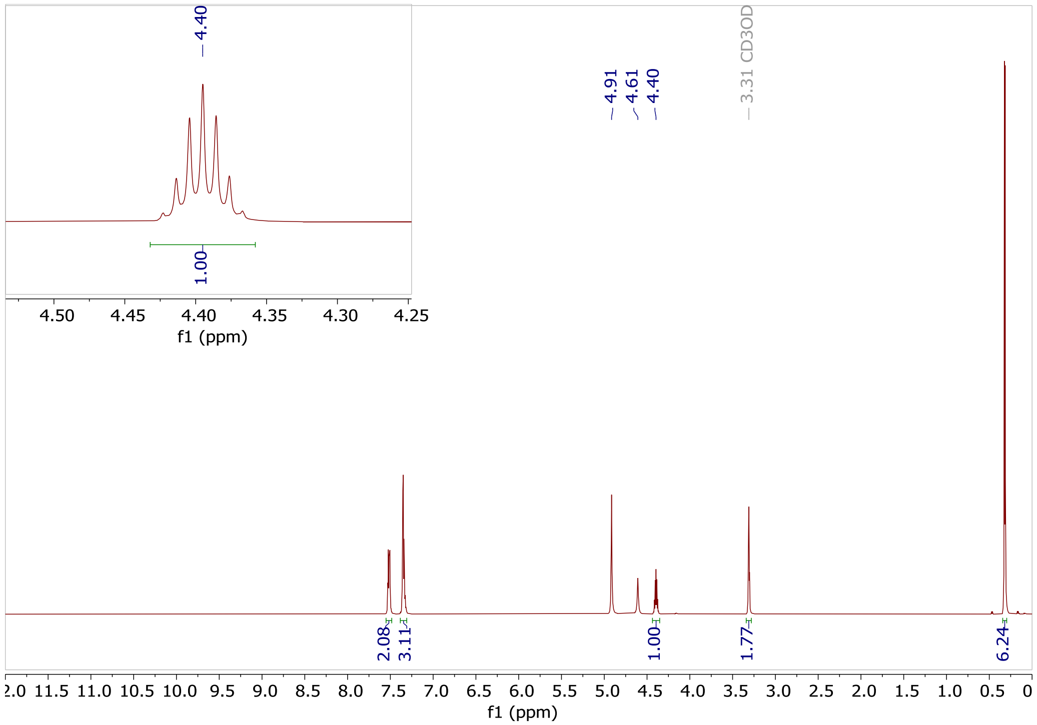

*Figure 34. ^{1}H-NMR of **9** in 10% D_2O:MeOD-d_4 at 10 minutes.*

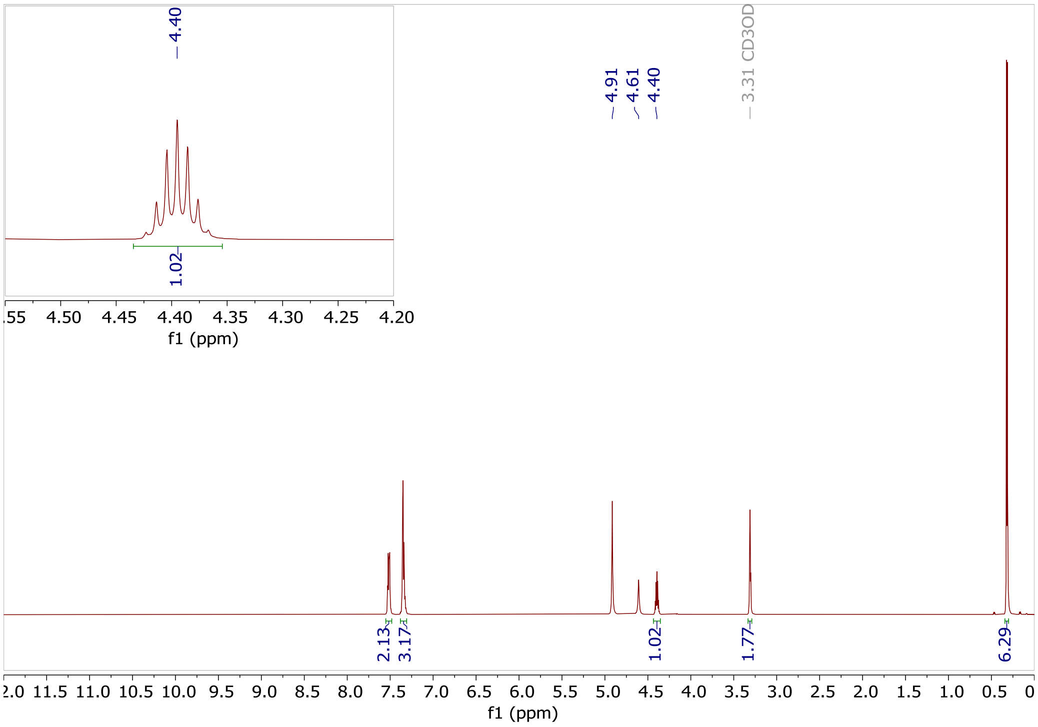

*Figure 35. ^{1}H-NMR of **9** in 10% D_2O:MeOD-d_4 at 60 minutes.*

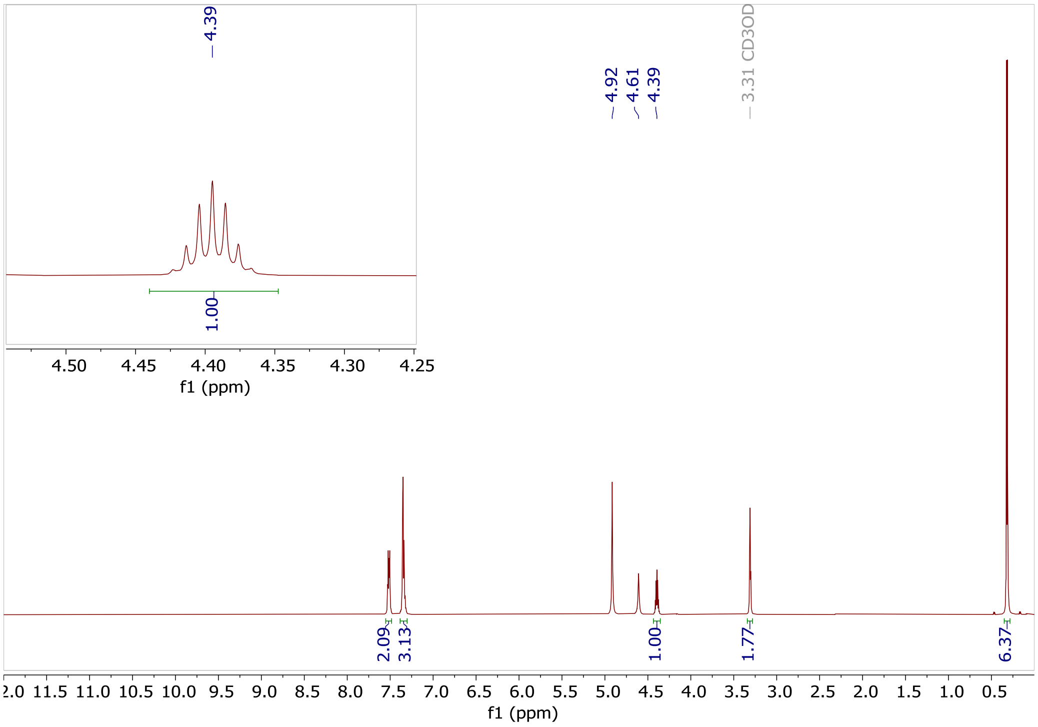

*Figure 36. ^{1}H-NMR of **9** in 10% D_2O:MeOD-d_4 at 24 hours.*

Diphenylmethylsilane **10**

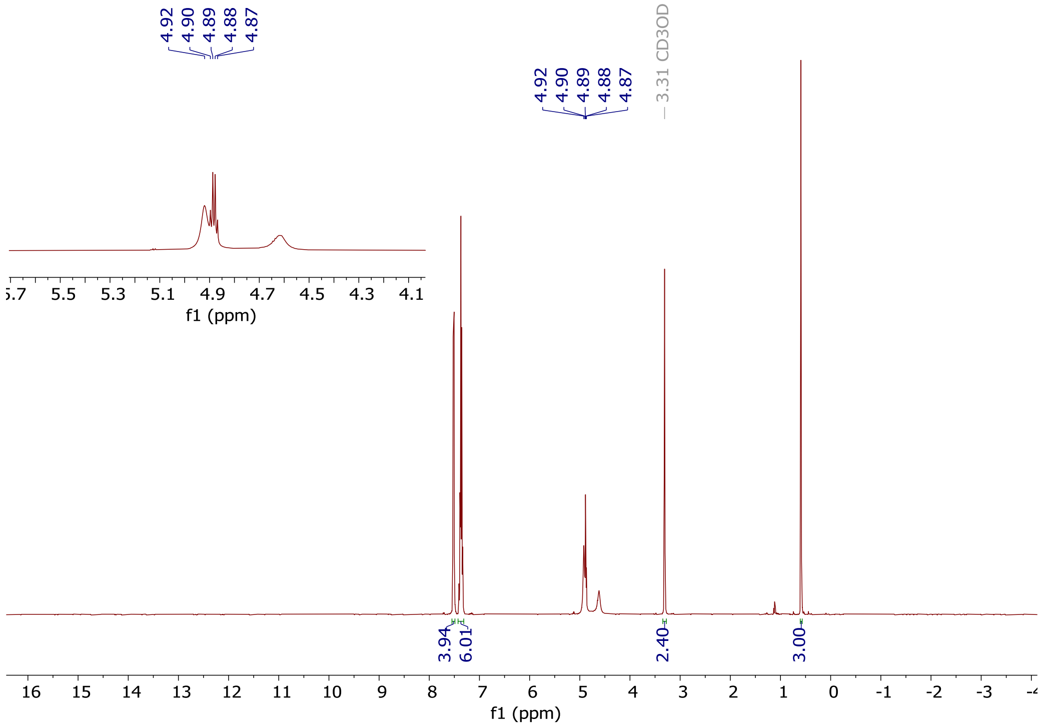

*Figure 37. ^{1}H-NMR of **10** in 10% D_2O:MeOD-d_4 at 0 minutes.*

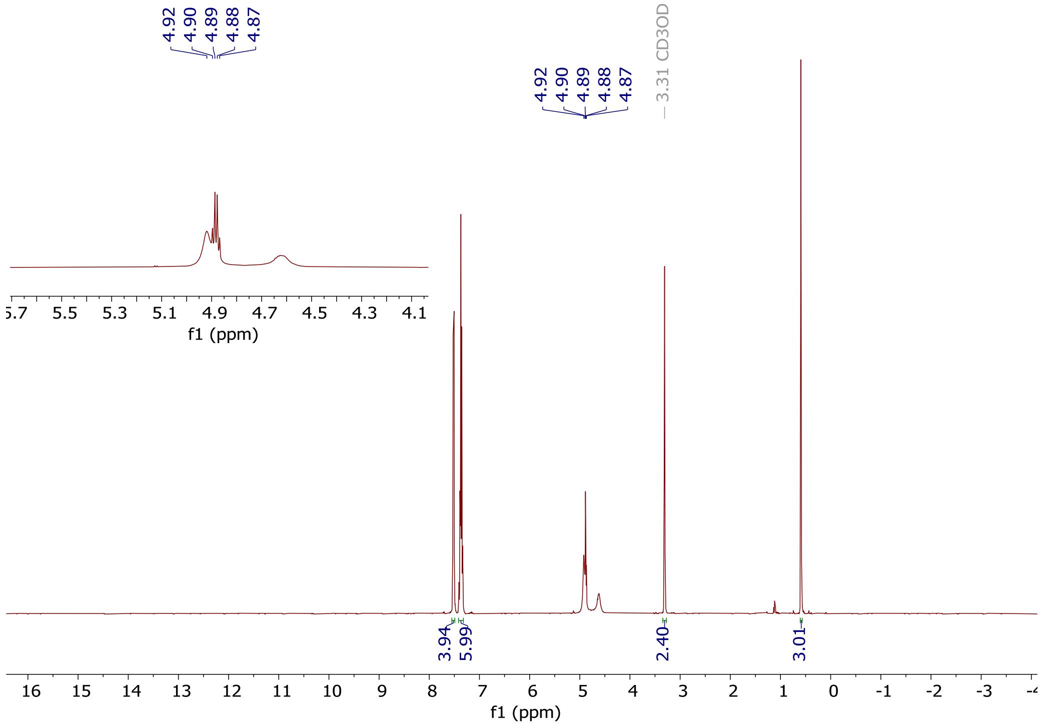

*Figure 38. ^{1}H-NMR of **10** in 10% D_2O:MeOD-d_4 at 10 minutes.*

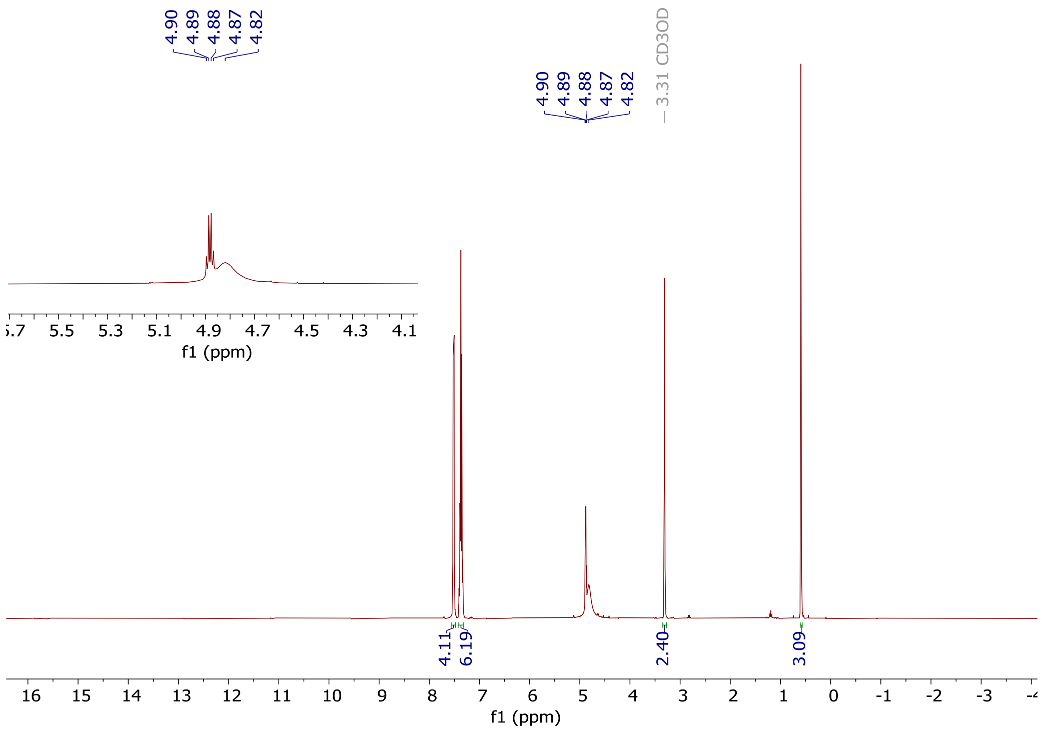

Figure 39. ^{1}*H-NMR of* ***10*** *in 10%* D_2O*:MeOD-*d_4 *at 60 minutes.*

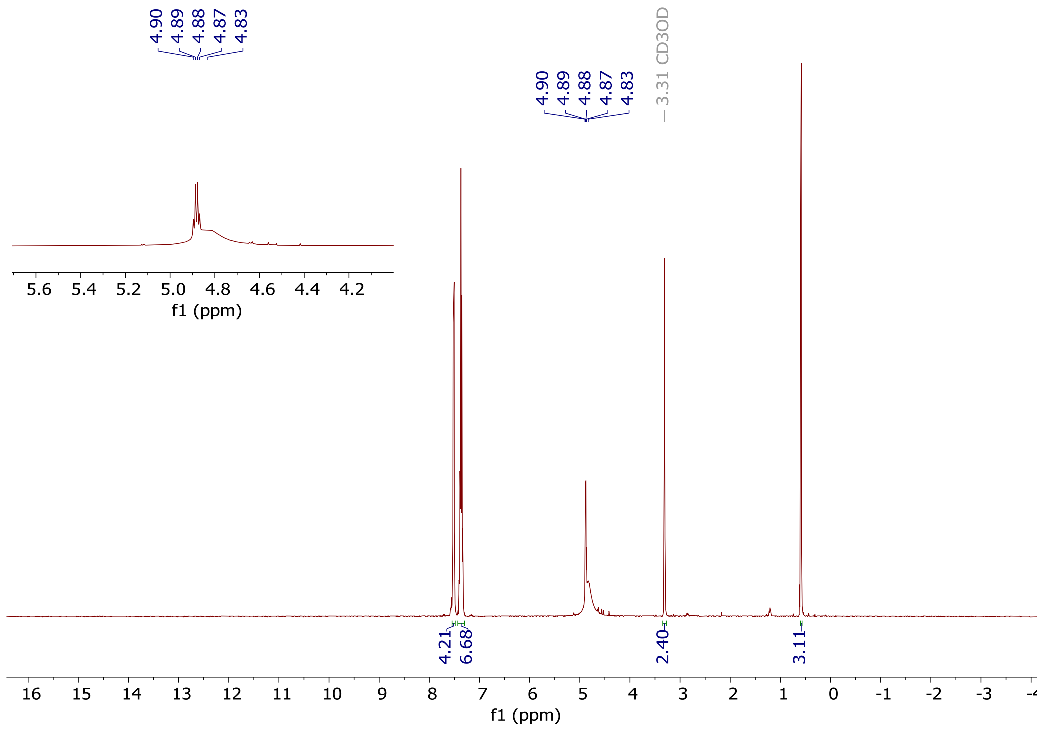

Figure 40. ^{1}*H-NMR of* ***10*** *in 10%* D_2O*:MeOD-*d_4 *at 24 hours.*

Reactivity in water evaluated using ^{1}H-NMR after liquid/liquid extraction (Study 2)

Experimental

To a flame-dried flask under nitrogen was added the silane (0.25 mmol) followed by distilled H_2O (2.5 mL, 0.1 M). The reaction was stirred under nitrogen for 1 hour, and then extracted using 5 x 5 mL EtOAc. The combined organic fraction was dried over $MgSO_4$, then concentrated under vacuum. The percent recovery was calculated based on the mass remaining after drying. The sample was dissolved in the appropriate deuterated solvent for ^{1}H-, ^{13}C-, ^{29}Si-NMR acquisition. To minimize evaporative loss of material, a modified extraction was used for the volatile hydrosilanes (phenylsilane, diethylsilane, triisopropylsilane, triethylsilane, and dimethylphenylsilane): the reaction was cooled to 0 °C, $MgSO_4$ powder was added, followed by 1 eq. of mesitylene internal standard and $CDCl_3$. The entire mixture was sonicated for 3 minutes and filtered into an NMR tube for NMR analysis.

Phenylsilane **1**

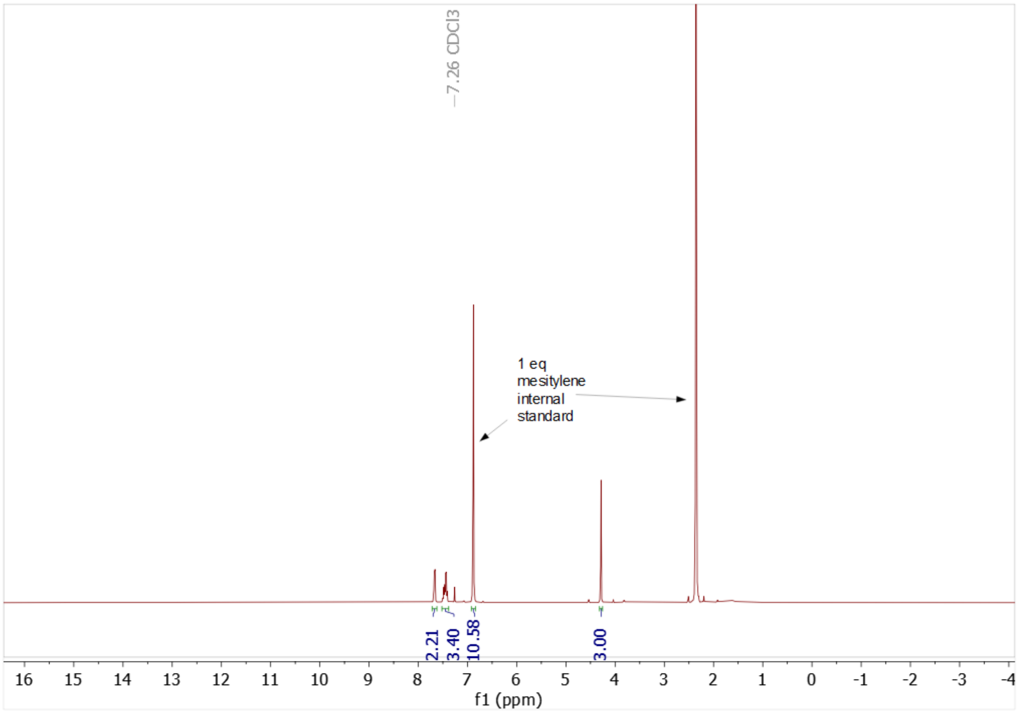

*Figure 41. ^{1}H-NMR ($CDCl_3$) of **1** after aqueous workup, with 1 equivalent of mesitylene internal standard.*

Dodecylsilane **2**

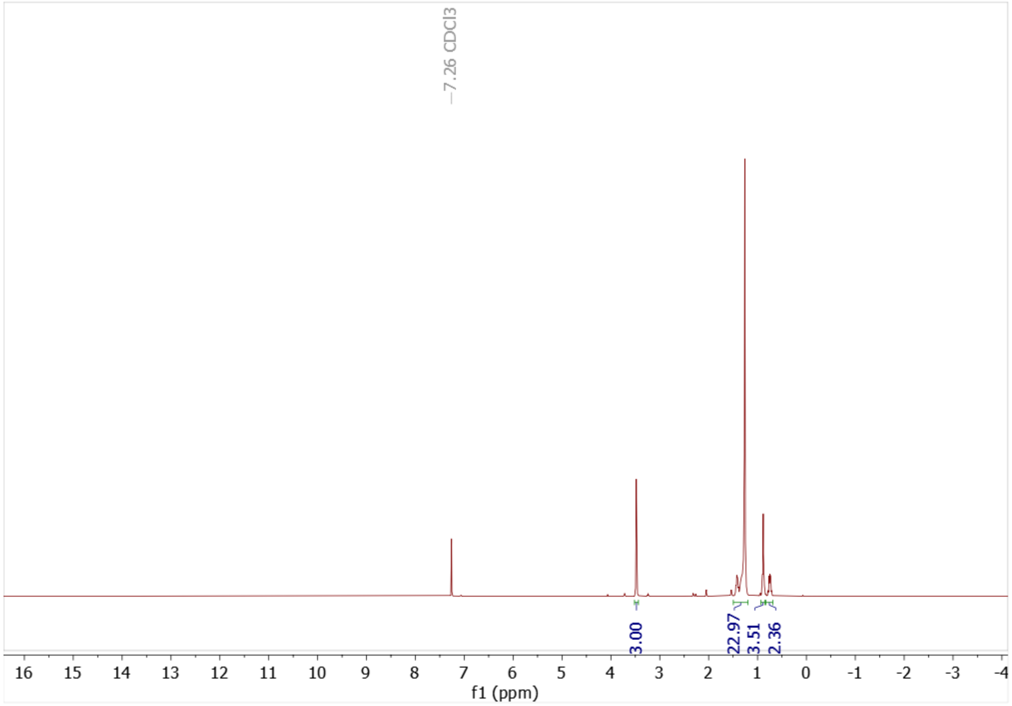

*Figure 42. ^{1}H-NMR ($CDCl_3$) of **2** after aqueous workup.*

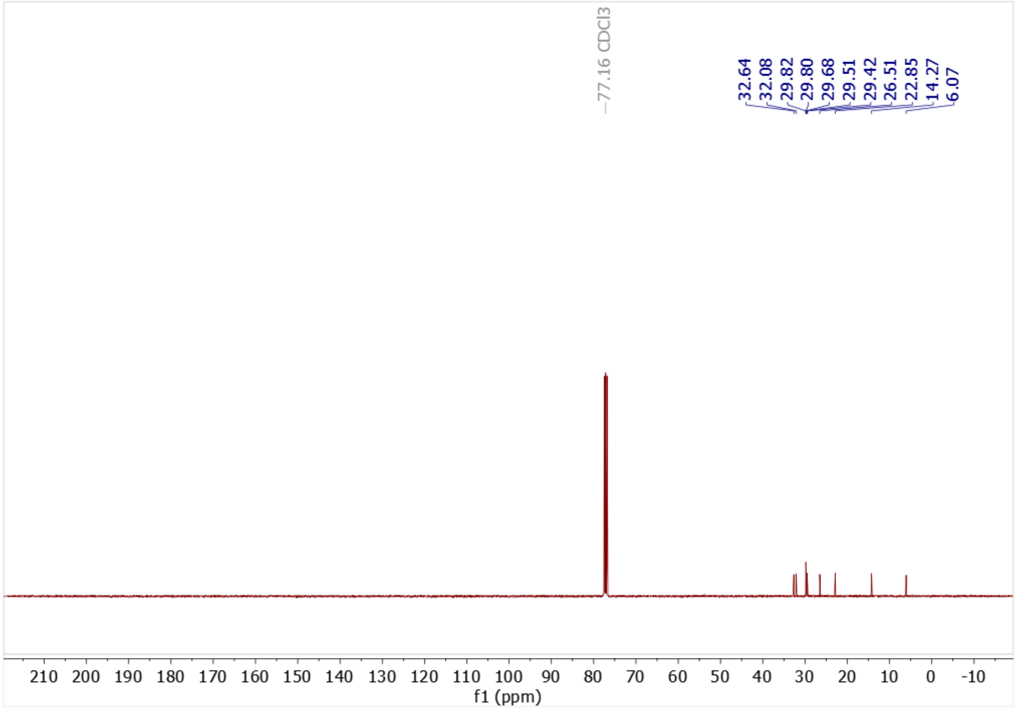

Figure 43. ^{13}*C-NMR (CDCl*$_3$*) of* ***2*** *after aqueous workup.*

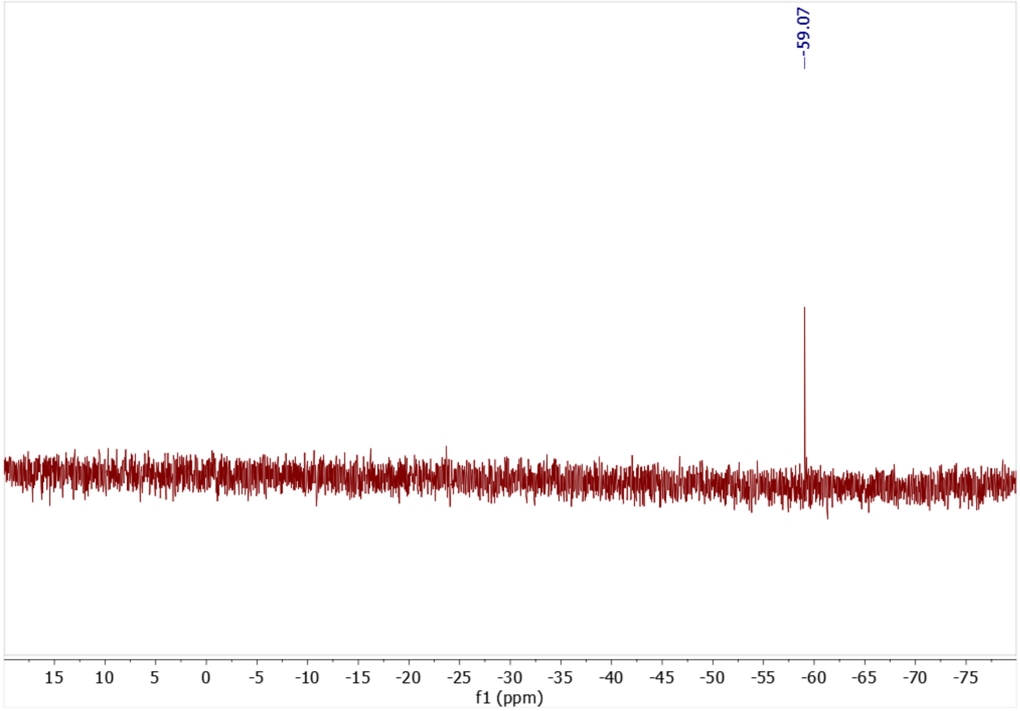

Figure 44. ^{29}*Si-NMR (CDCl*$_3$*) of* ***2*** *after aqueous workup.*

Diphenylsilane **3**

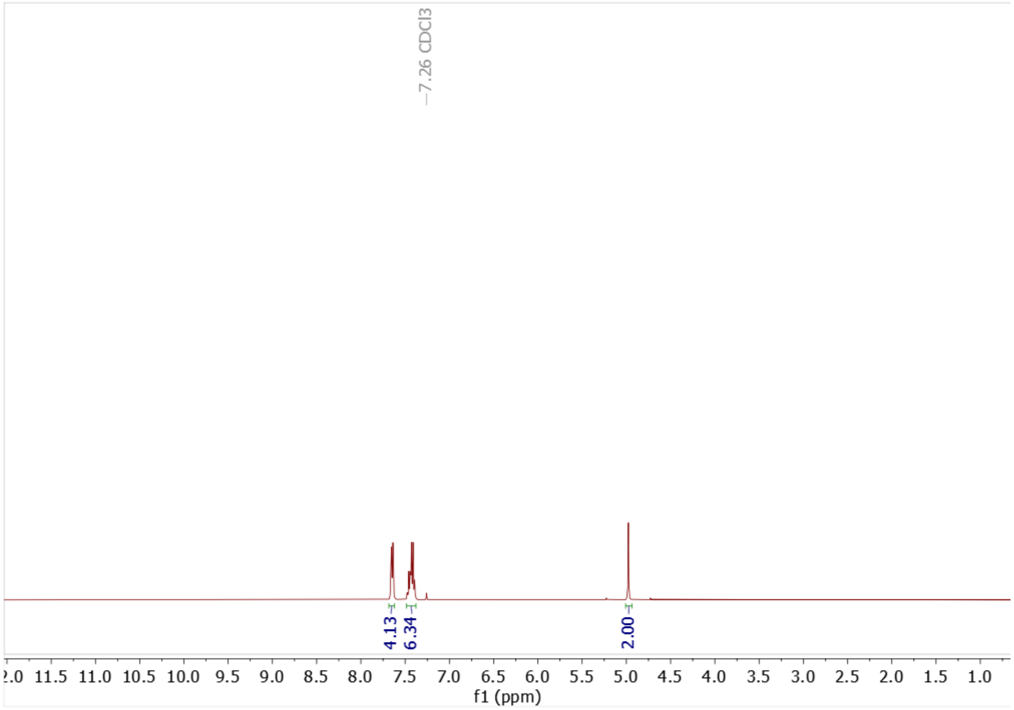

*Figure 45. ^{1}H-NMR ($CDCl_3$) of **3** after aqueous workup.*

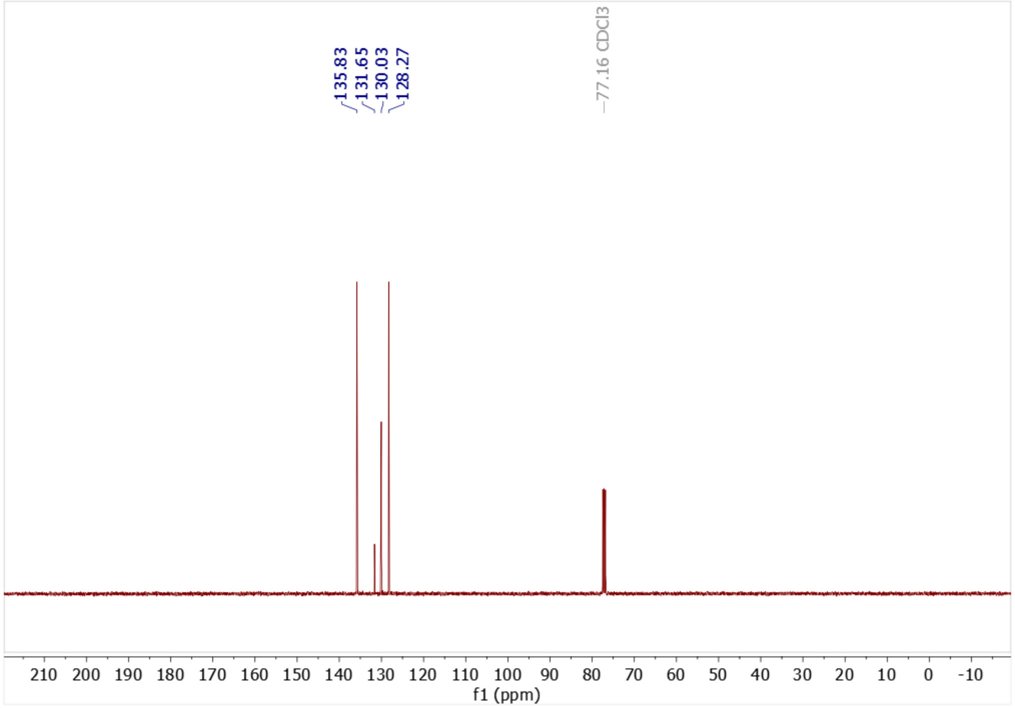

*Figure 46. ^{13}C-NMR ($CDCl_3$) of **3** after aqueous workup.*

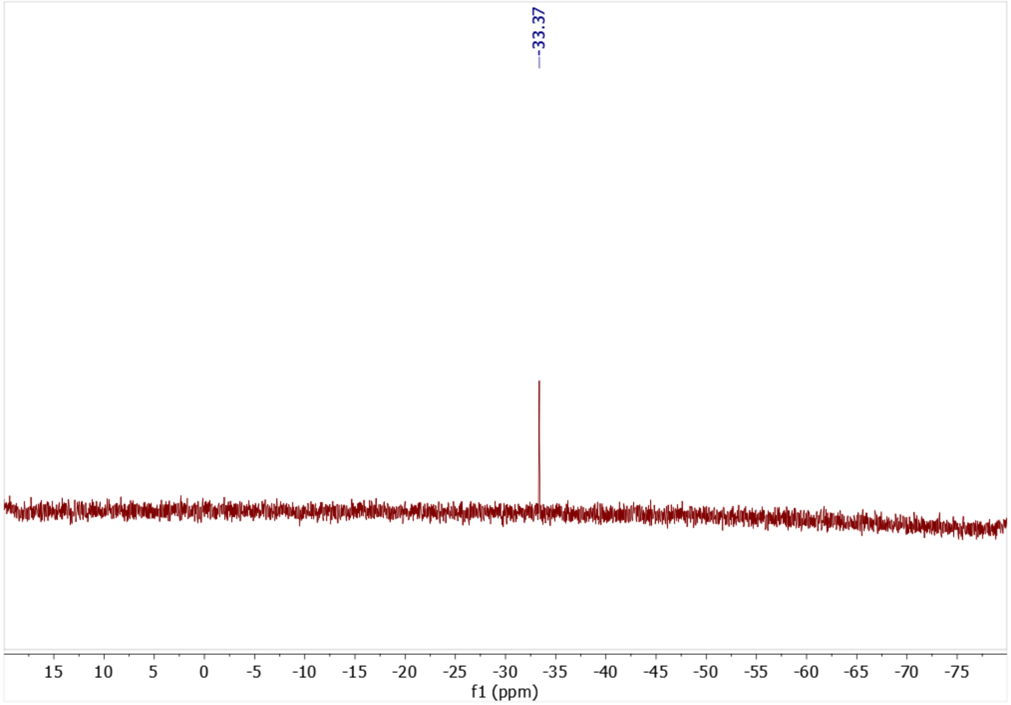

Figure 47. ^{29}*Si-NMR (CDCl*$_3$*) of* ***3*** *after aqueous workup.*

Diethylsilane **4**

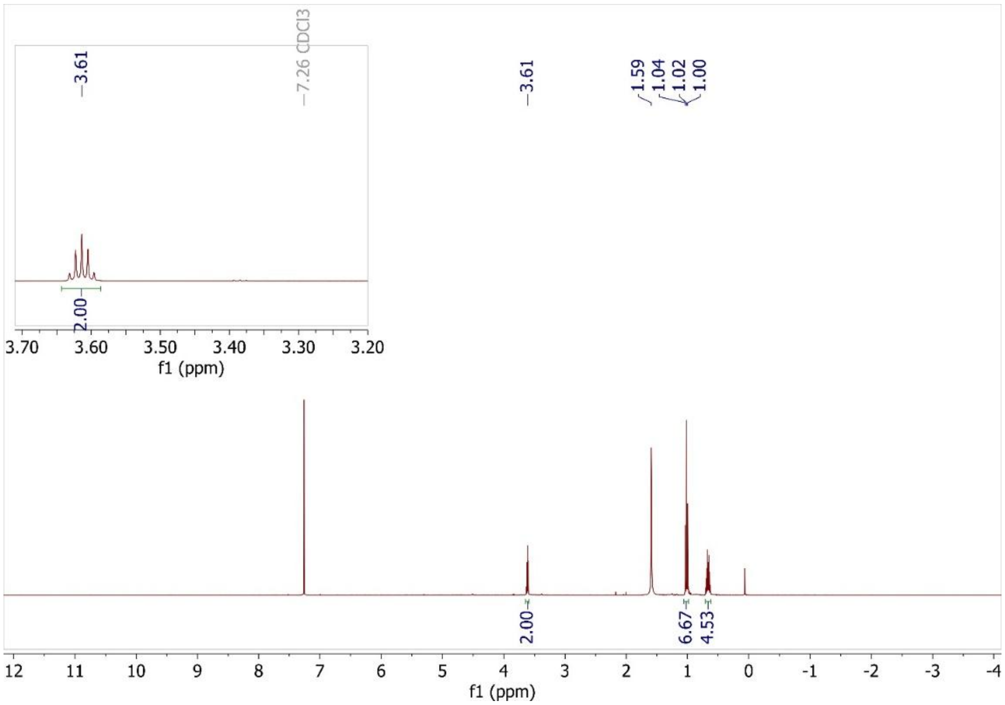

Figure 48. ^{1}*H-NMR (CDCl*$_3$*) of* ***4*** *after aqueous workup.*

n-Hexylphenylsilane **5**

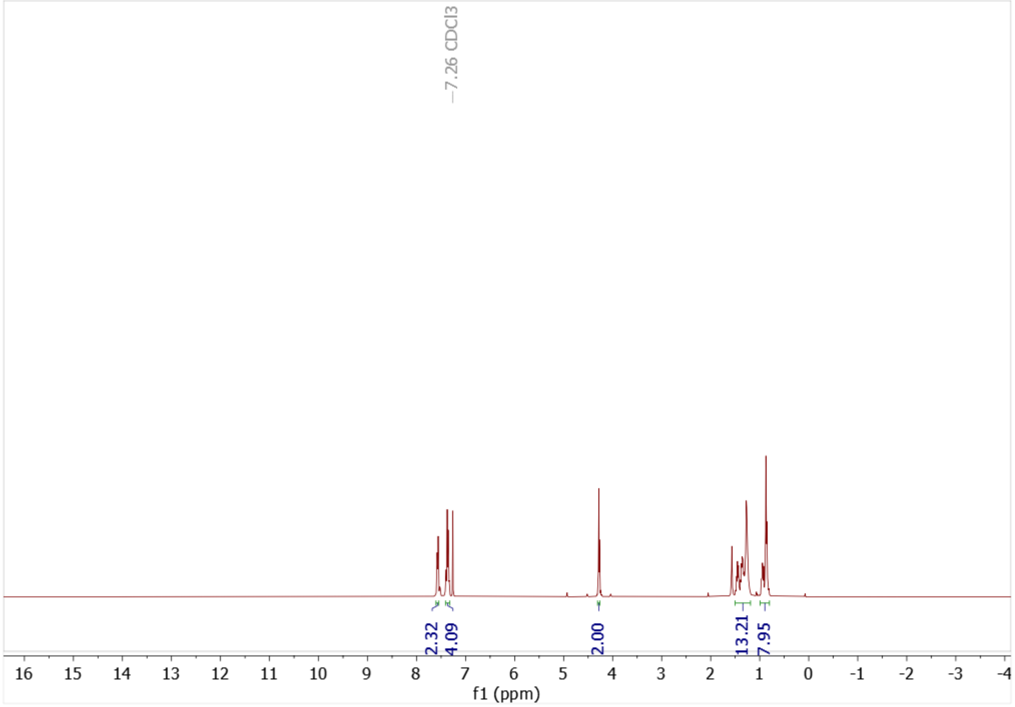

*Figure 49. ^{1}H-NMR ($CDCl_3$) of **5** after aqueous workup.*

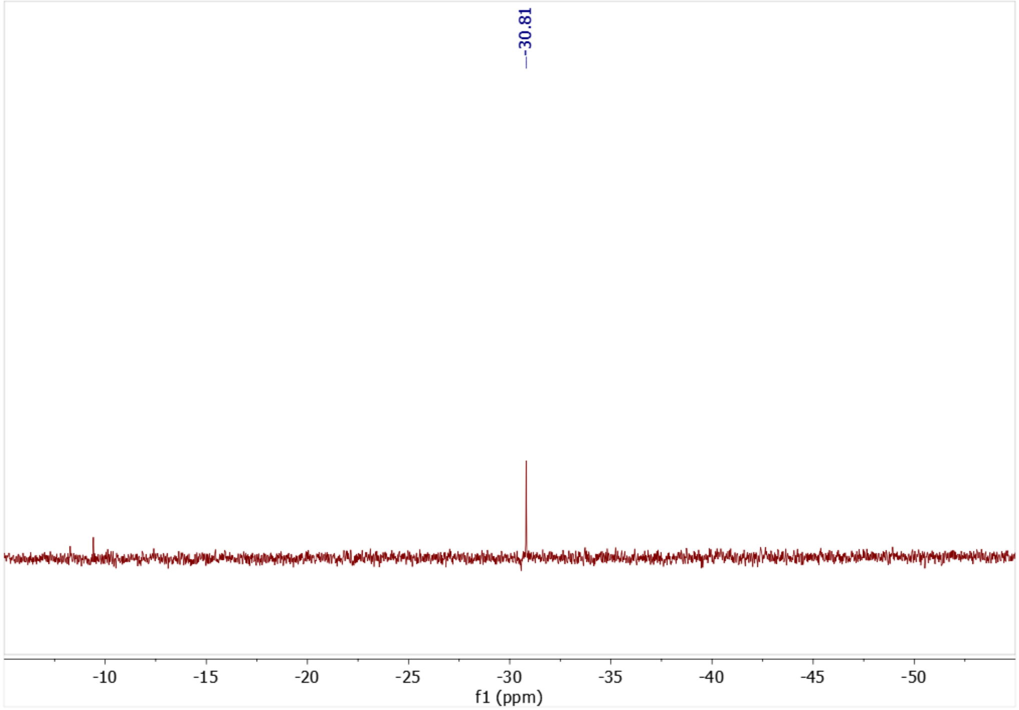

*Figure 50. ^{29}Si-NMR ($CDCl_3$) of **5** after aqueous workup.*

Triphenylsilane **6**

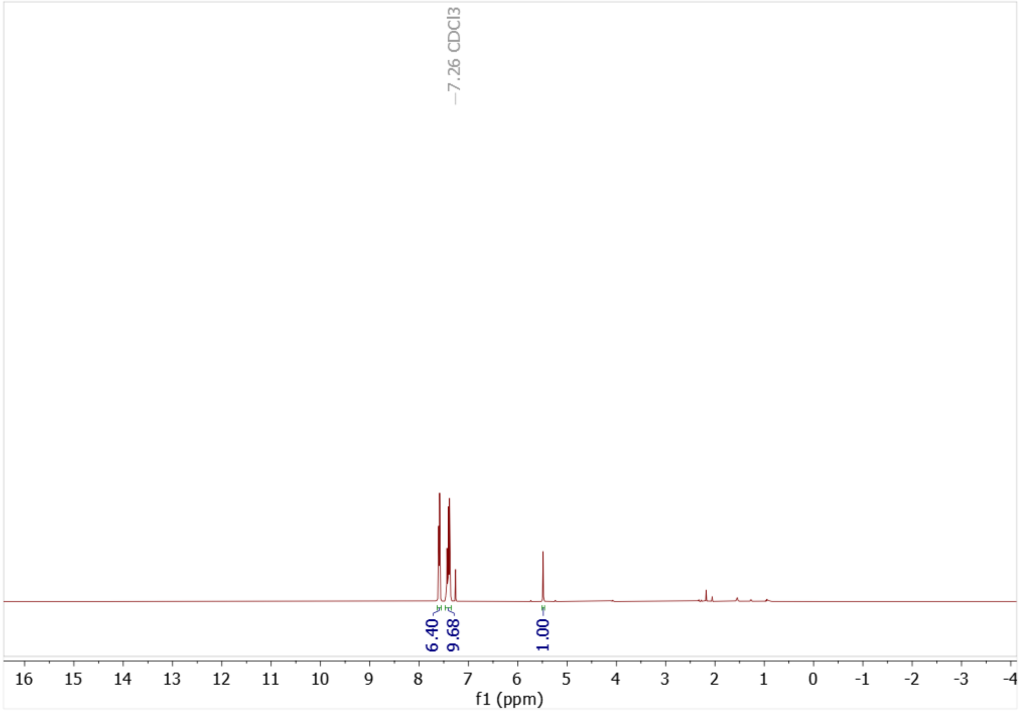

*Figure 51. ^{1}H-NMR ($CDCl_3$) of **6** after aqueous workup.*

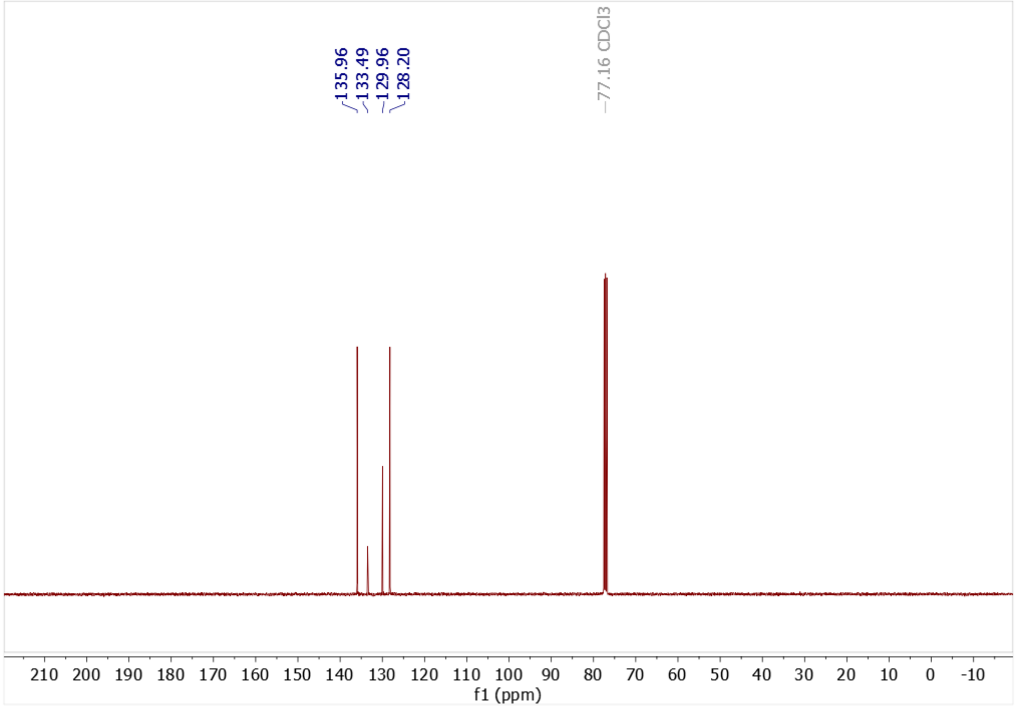

*Figure 52. ^{13}C-NMR ($CDCl_3$) of **6** after aqueous workup.*

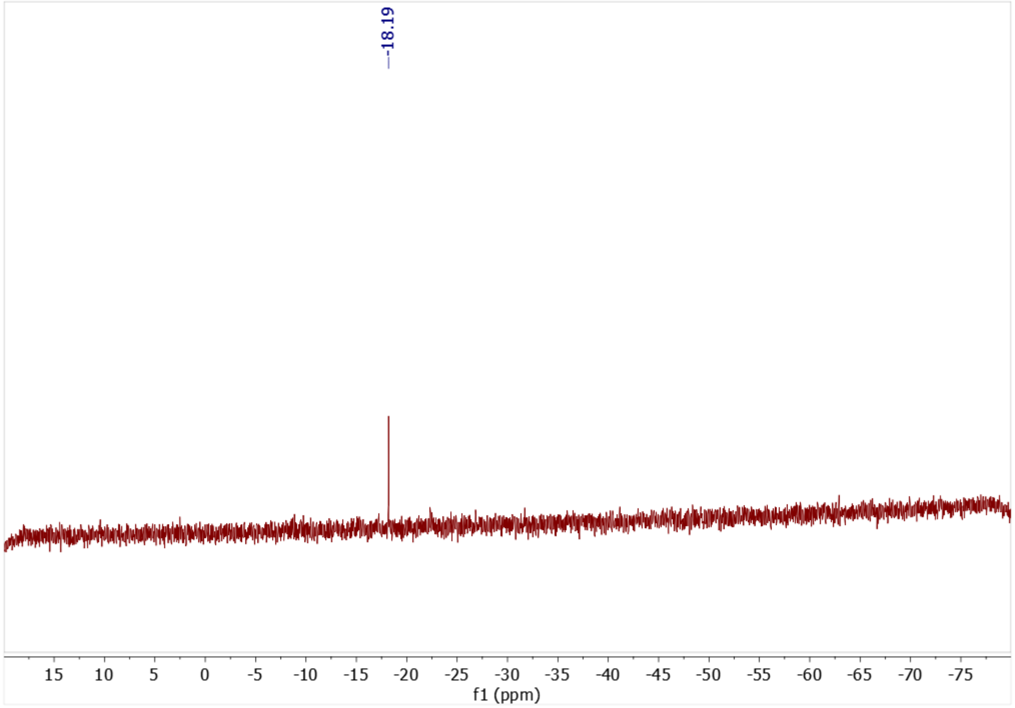

*Figure 53. ^{29}Si-NMR ($CDCl_3$) of **6** after aqueous workup.*

Triethylsilane **7**

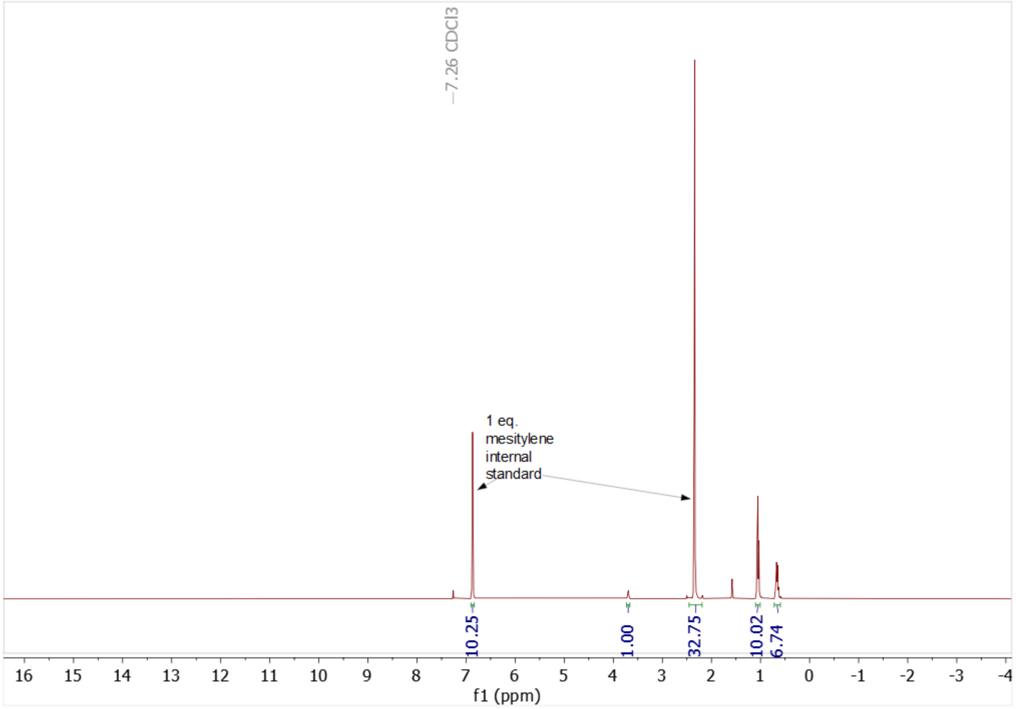

*Figure 54. ^{1}H-NMR ($CDCl_3$) of **7** after aqueous workup, with 1 equivalent of mesitylene internal standard.*

Triisopropylsilane **8**

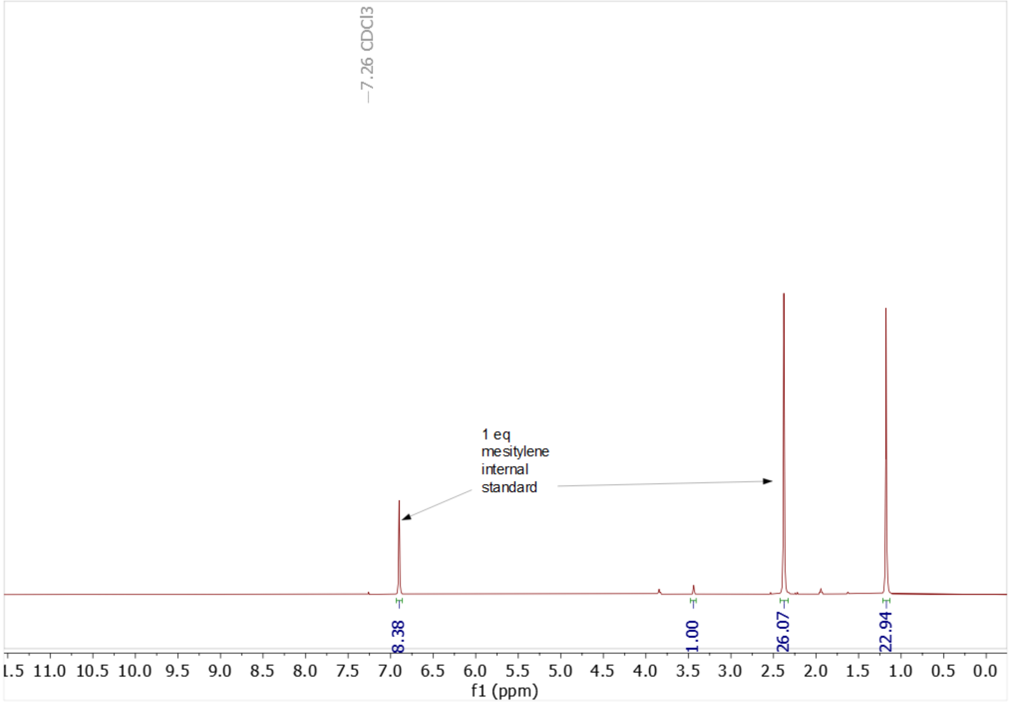

*Figure 55. ^{1}H-NMR ($CDCl_3$) of **8** after aqueous workup, with 1 equivalent of mesitylene internal standard.*

Dimethylphenylsilane **9**

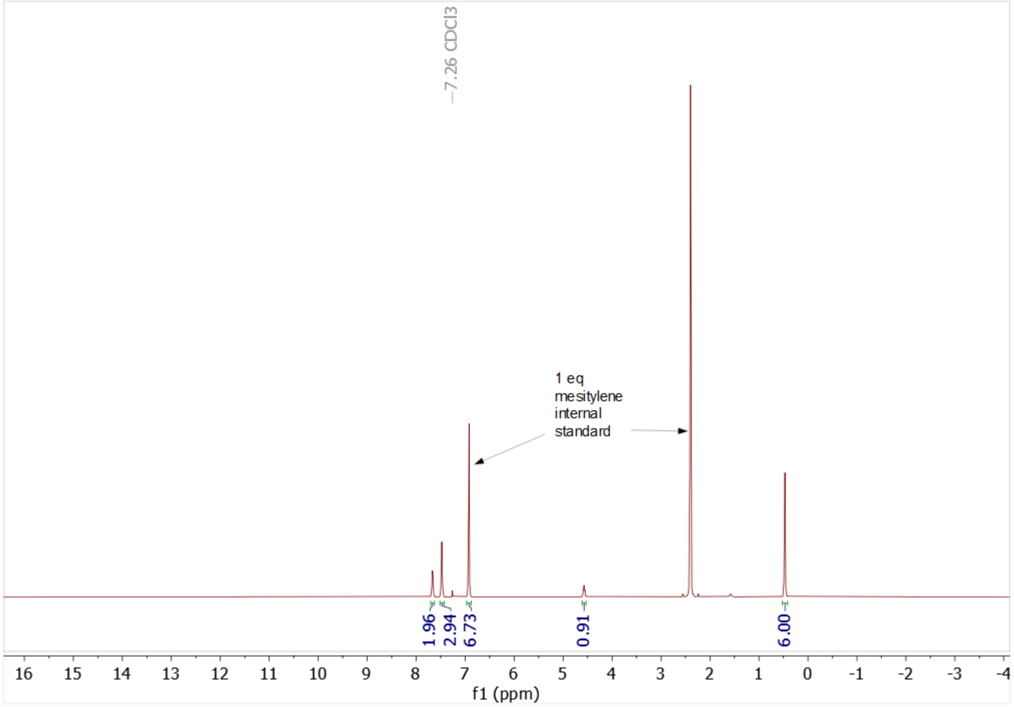

*Figure 56. ^{1}H-NMR ($CDCl_3$) of **9** after aqueous workup, with 1 equivalent of mesitylene internal standard.*

Diphenylmethylsilane **10**

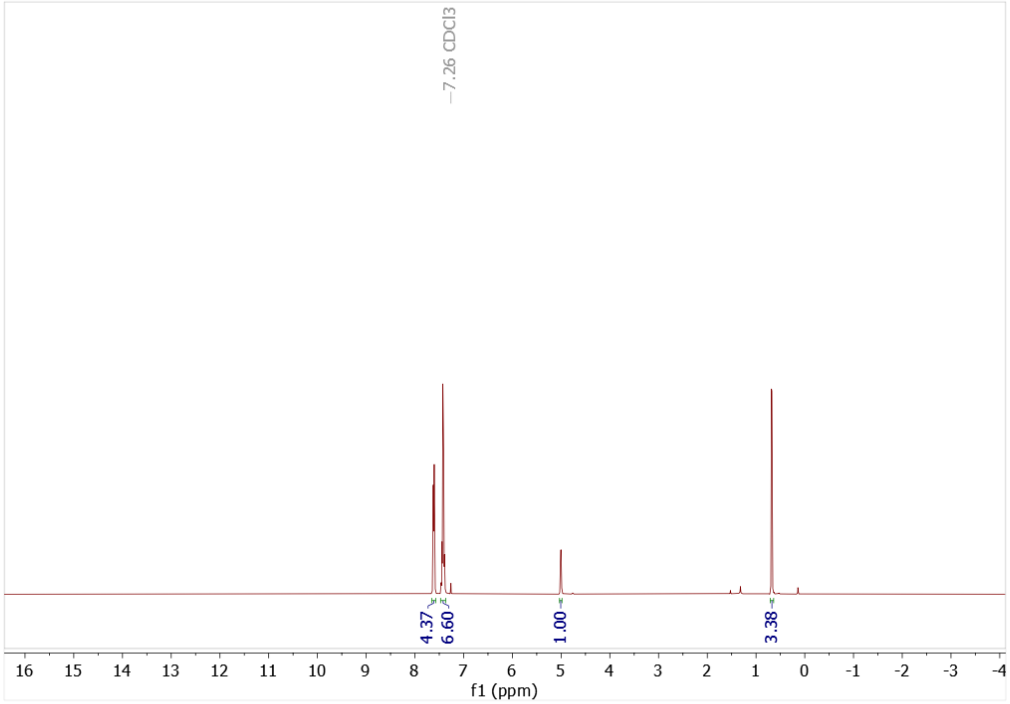

*Figure 57. ^{1}H-NMR ($CDCl_3$) of **10** after aqueous workup.*

Reactivity in water evaluated using ^{1}H-NMR after direct removal of solvent *in vacuo* (Study 3)

Experimental

To a flame-dried flask under nitrogen was added silane (0.25 mmol) followed by distilled H_2O (1 mL, 0.25 M). The reaction was stirred under nitrogen for 1 hour and then concentrated under vacuum to remove H_2O. The sample was reconstituted in the appropriate deuterated solvent for NMR acquisition.

Phenylsilane **1**

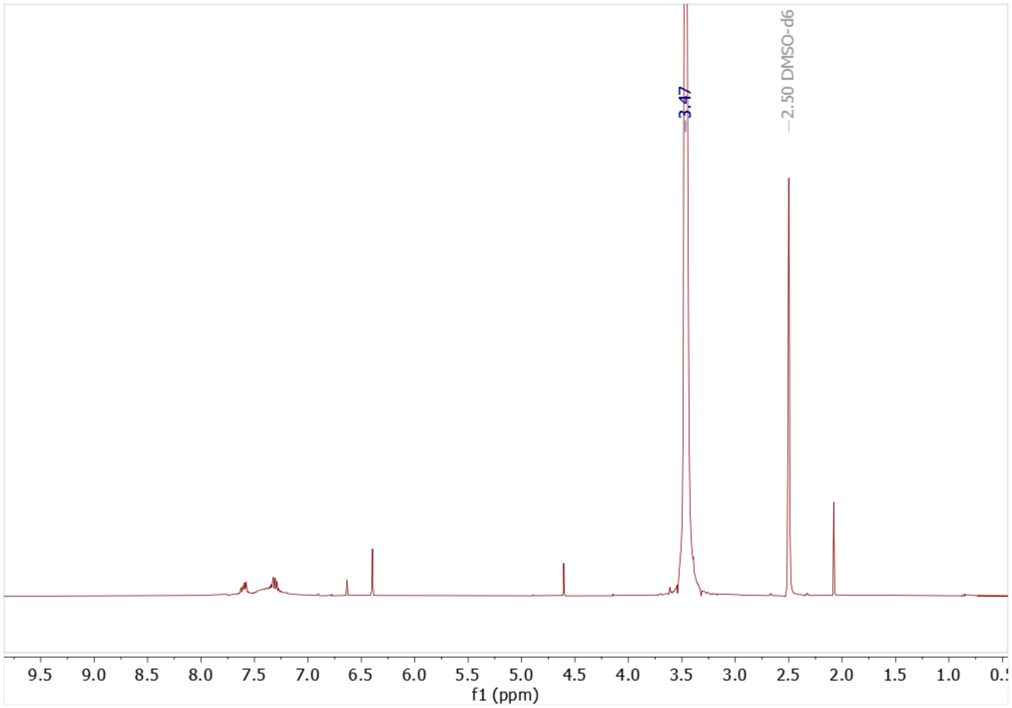

*Figure 58. ^{1}H-NMR (DMSO-d_6) of **1** after direct removal of solvent in vacuo.*

Dodecylsilane **2**

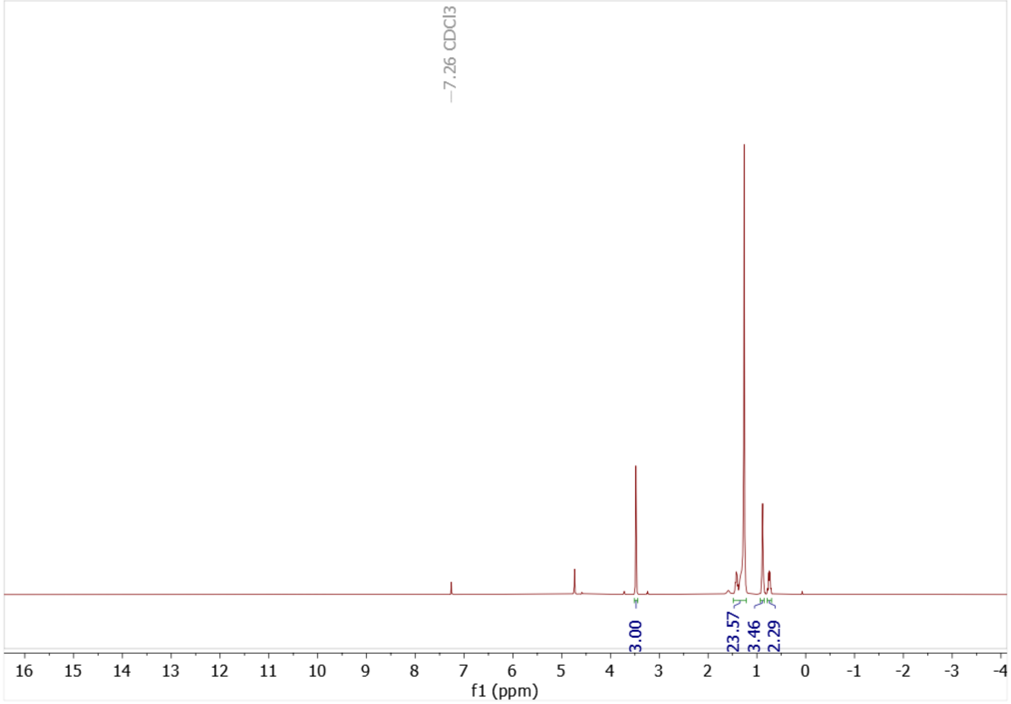

*Figure 59. ^{1}H-NMR ($CDCl_3$) of **2** after direct removal of solvent in vacuo.*

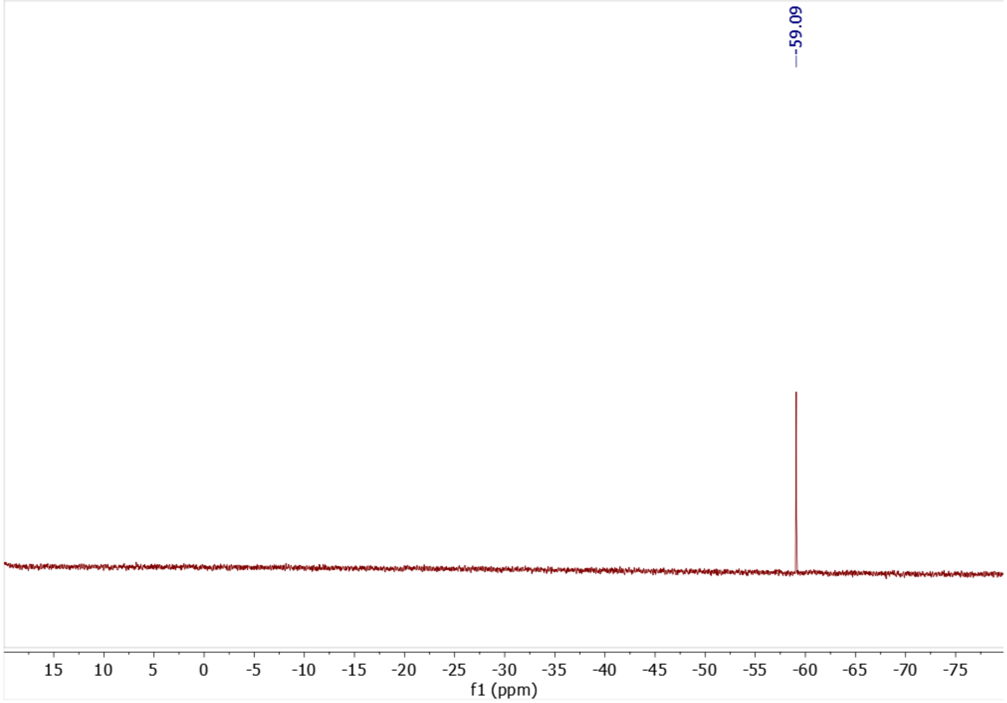

*Figure 60. ^{29}Si-NMR ($CDCl_3$) of **2** after direct removal of solvent in vacuo.*

Diphenylsilane **3**

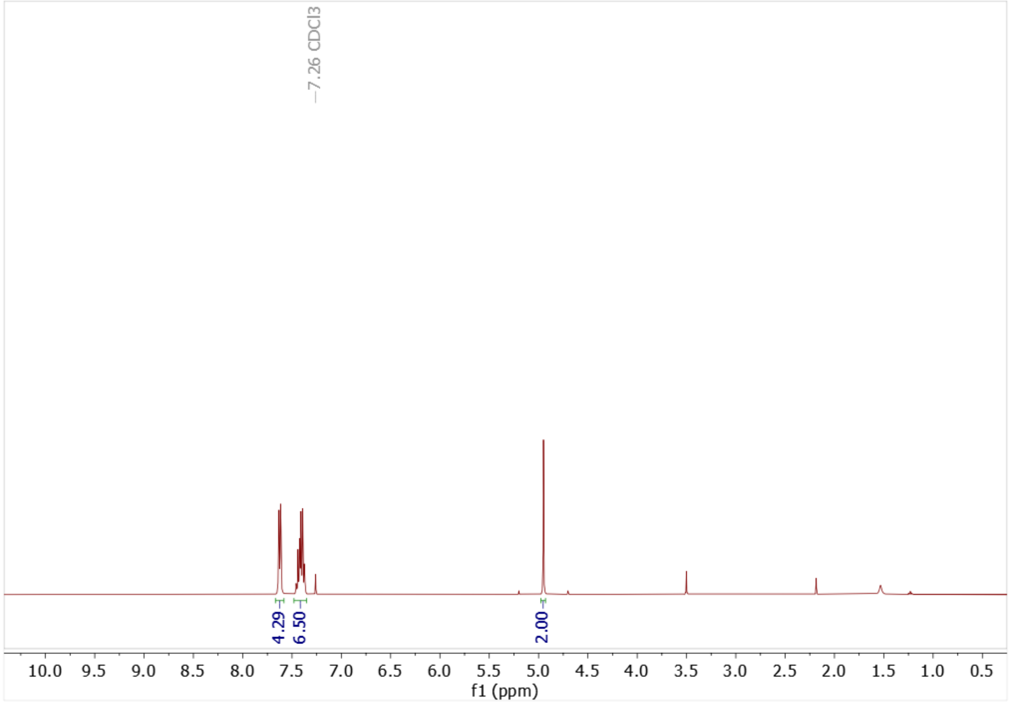

*Figure 61. ^{1}H-NMR ($CDCl_3$) of **3** after direct removal of solvent in vacuo.*

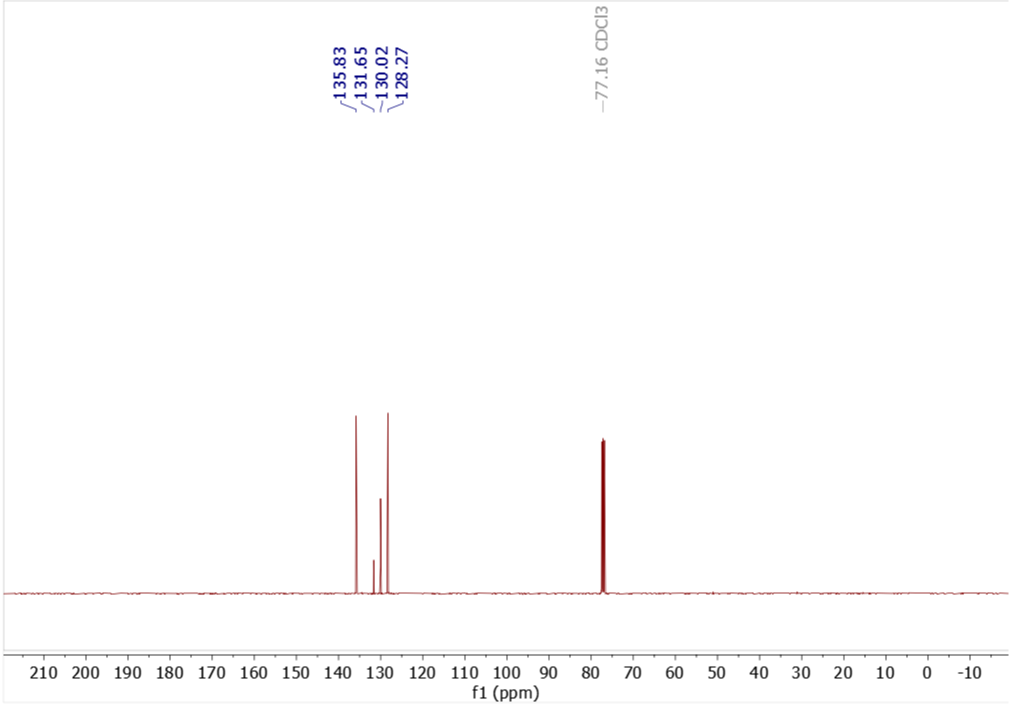

*Figure 62. ^{13}C-NMR ($CDCl_3$) of **3** after direct removal of solvent in vacuo.*

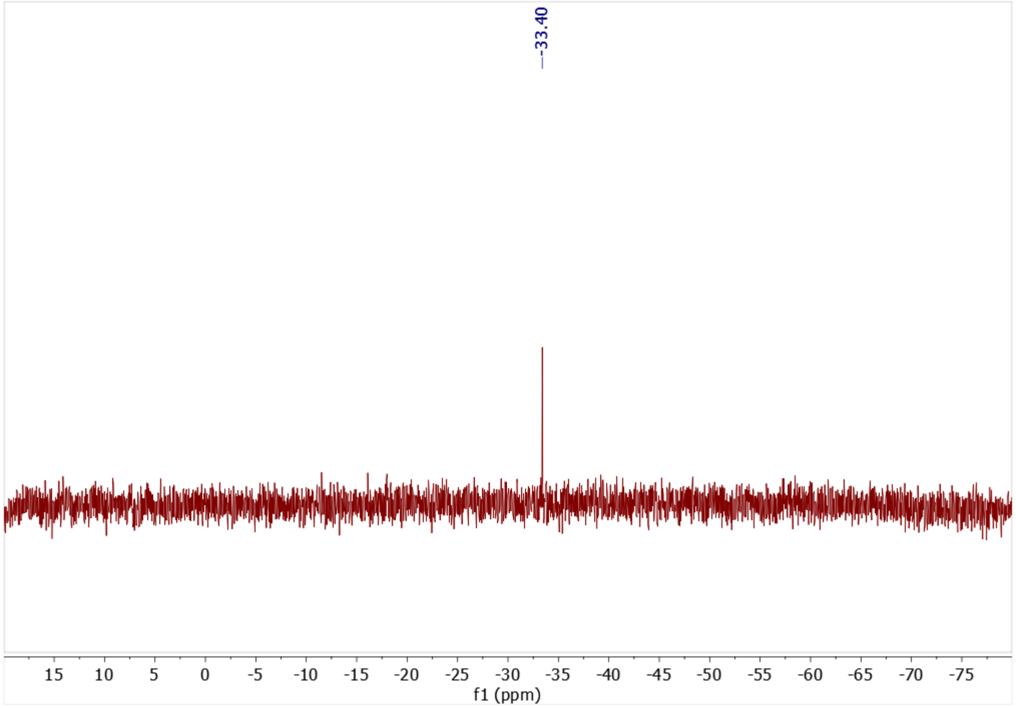

*Figure 63. ^{29}Si-NMR ($CDCl_3$) of **3** after direct removal of solvent in vacuo.*

n-Hexylphenylsilane **5**

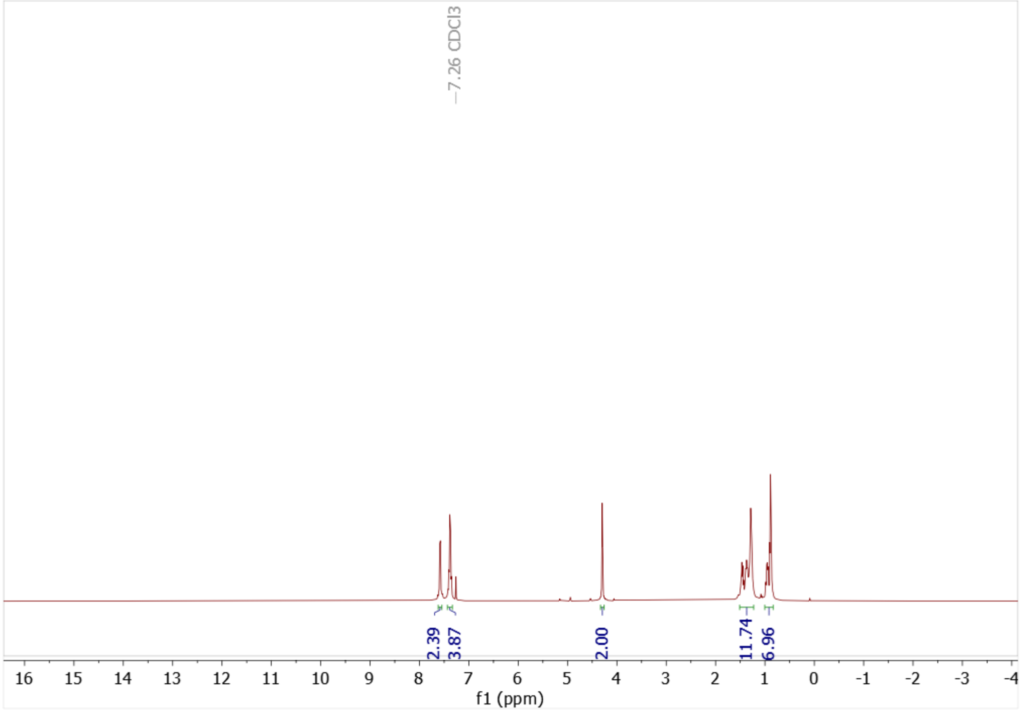

*Figure 64. ^{1}H-NMR ($CDCl_3$) of **5** after direct removal of solvent in vacuo.*

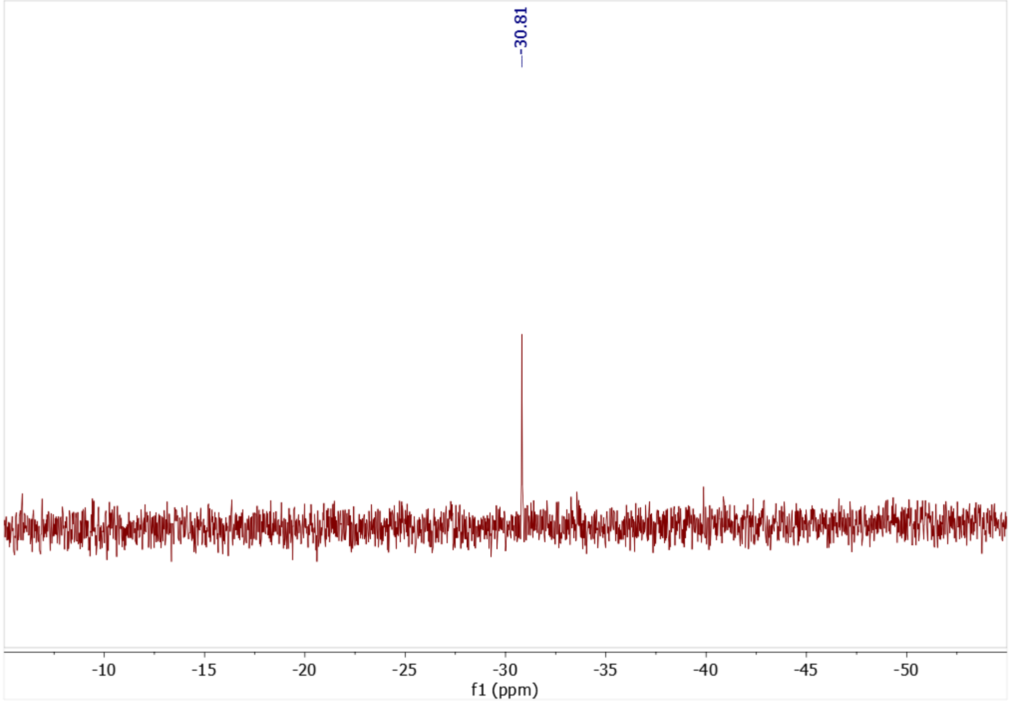

*Figure 65. ^{29}Si-NMR ($CDCl_3$) of **5** after direct removal of solvent in vacuo.*

Triphenylsilane **6**

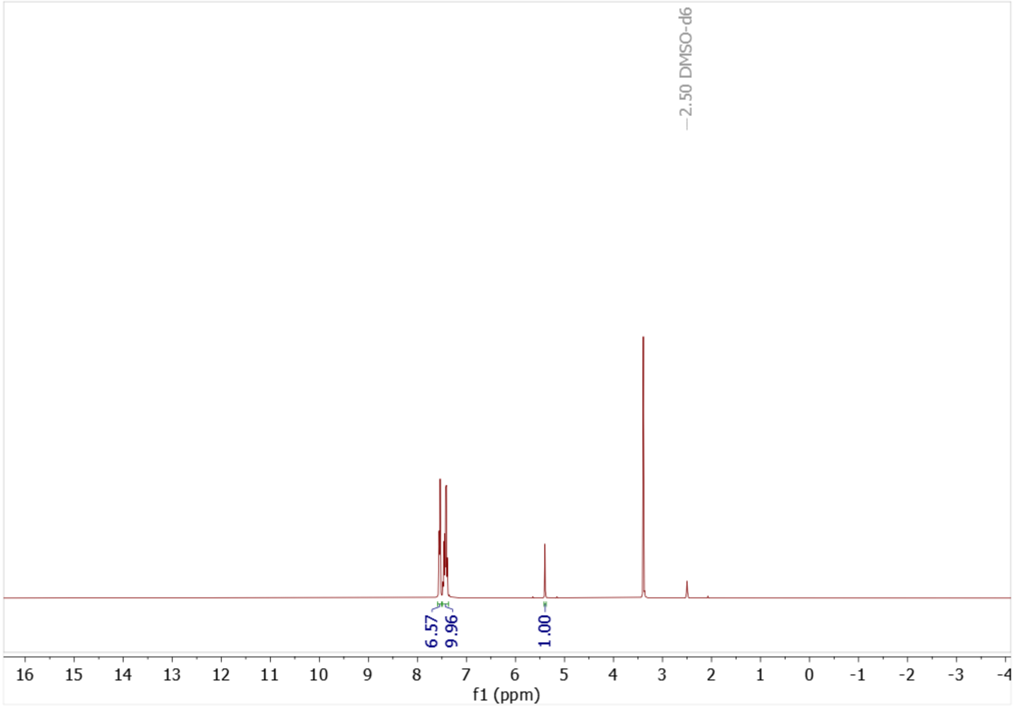

*Figure 66. ^{1}H-NMR (DMSO-d$_6$) of **6** after direct removal of solvent in vacuo.*

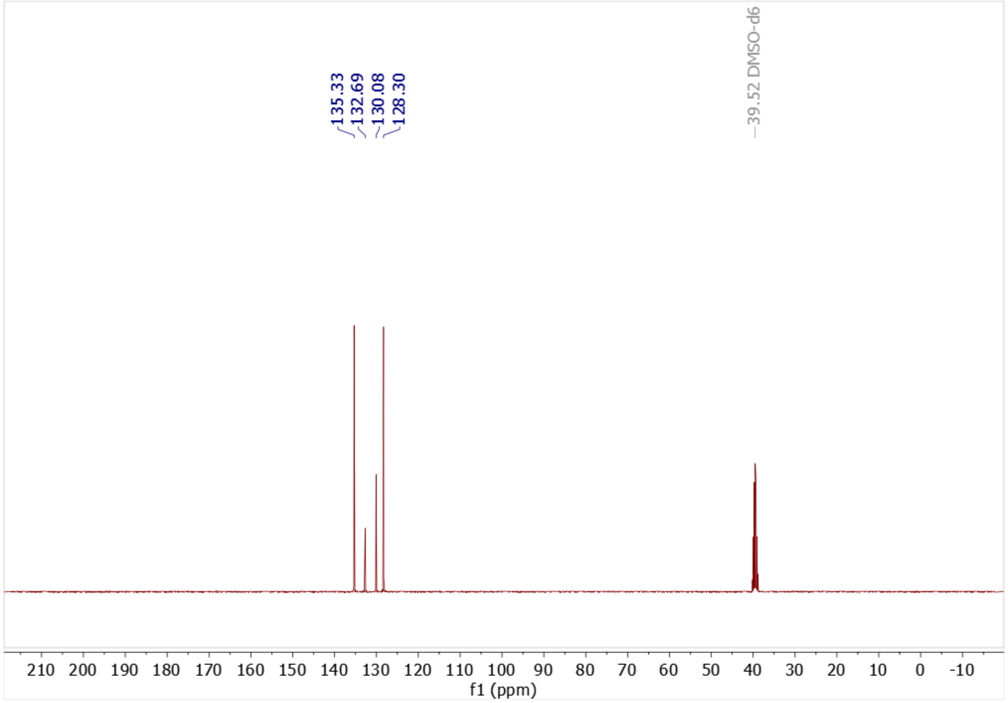

*Figure 67. ^{13}C-NMR (DMSO-d$_6$) of **6** after direct removal of solvent in vacuo.*

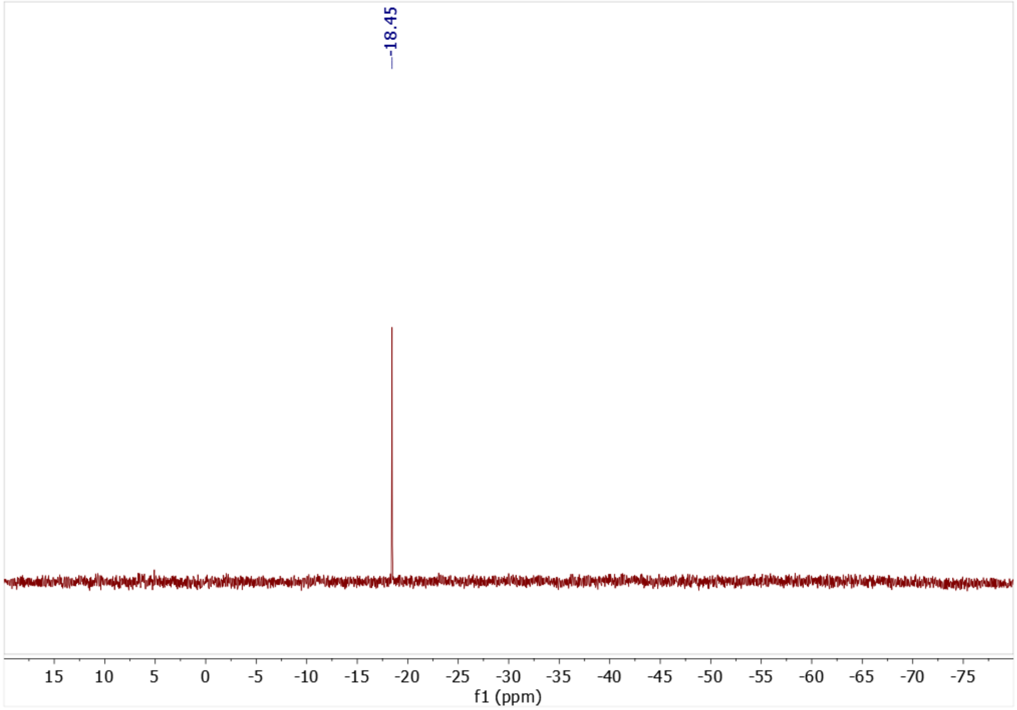

*Figure 68. ^{29}Si-NMR (DMSO-d_6) of **6** after direct removal of solvent in vacuo.*

Diphenylmethylsilane **10**

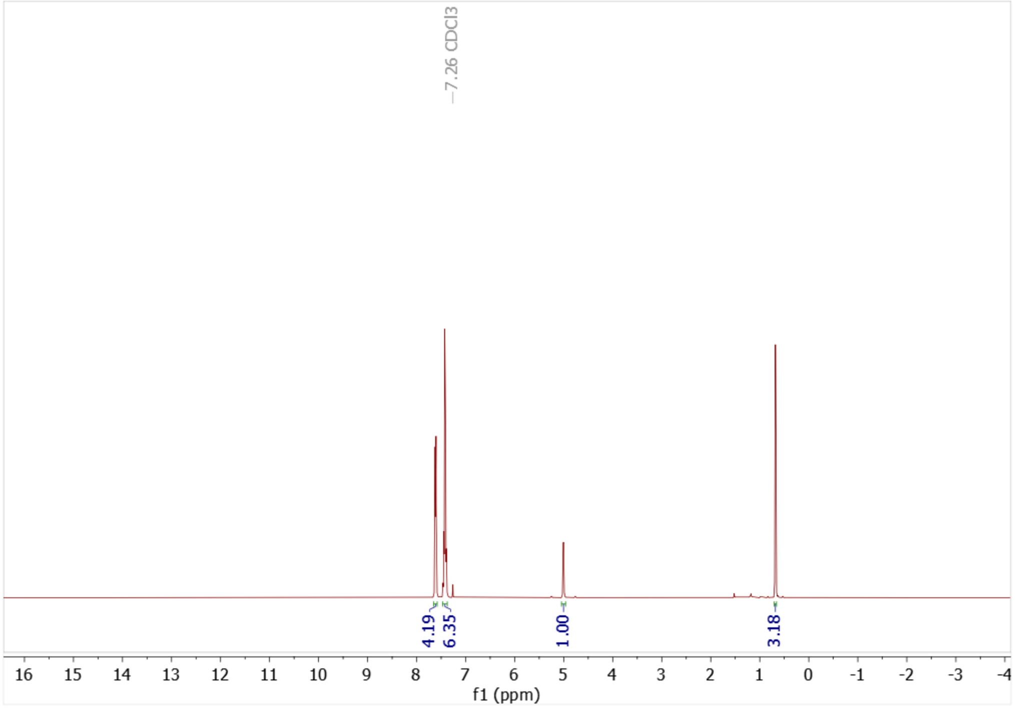

*Figure 69. ^{1}H-NMR ($CDCl_3$) of **10** after direct removal of solvent in vacuo.*

Reactivity in water evaluated using GCMS (Study 4)

Experimental

Calibration curve:

5 different concentrations (0.005 M, 0.010 M, 0.015 M, 0.020 M, and 0.025 M) of hydrosilane in ethyl acetate were prepared and ran through the GCMS. A calibration curve was created from the data performed in triplicates. For diethylsilane, an additional sixth calibration point at 0.0010 M was prepared.

Preparation of reaction trials:

Hydrosilane (0.5 mmol) added to a vial, followed by 5 mL of distilled water, and then stirred for 1 hour. The reaction was transferred to a separatory funnel and extracted with 25 mL of ethyl acetate. An aliquot (1.5 mL) of this organic layer was then transferred to a GC vial and then 0.5 μL was injected (via autosampler) into the GCMS to determine the amount of leftover hydrosilane. This was repeated three times, and the average response was used to calculate the concentration of hydrosilane in the organic layer.

GCMS acquisition method:

The GCMS system was Agilent 5977C GC/MSD with an electron impact (EI) ionization technique. The inlet was set to 250 °C, and the injection volume was (0.5 μL). The GC oven was kept at 50 °C for 1 minute and then ramped to 200 °C at a rate of 10 °C per minute unless otherwise specified. For triphenylsilane, the GC oven was kept at 80 °C for 1 minute and then ramped to 280 °C at a rate of 10 °C per minute. For diethylsilane, the GC oven was kept at 40 °C and held for 5 minutes. For triethylsilane, the GC oven was kept at 30 °C for 1 minute and then ramped to 180 °C at a rate of 10 °C per minute. For diphenylmethylsilane, the GC oven was kept at 80 °C for 1 minute and then ramped to 290 °C at a rate of 10 °C per minute. The carrier gas was He for all runs.

Phenylsilane **1**

Fragmentor Voltage **Collision Energy** 0 **Ionization Mode** EI

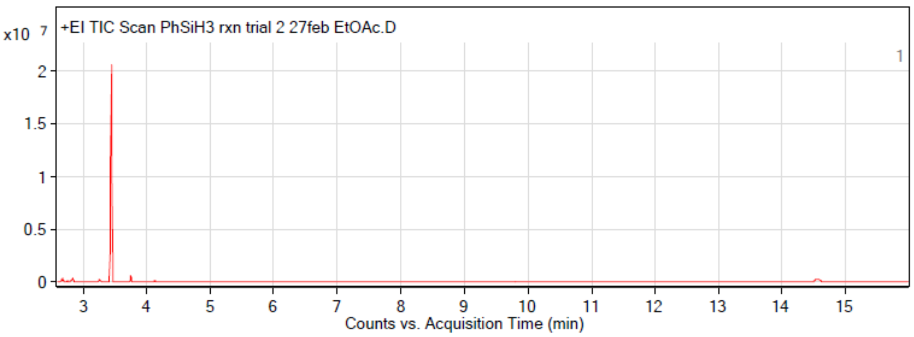

Figure 70. Chromatogram of $PhSiH_3$ reaction

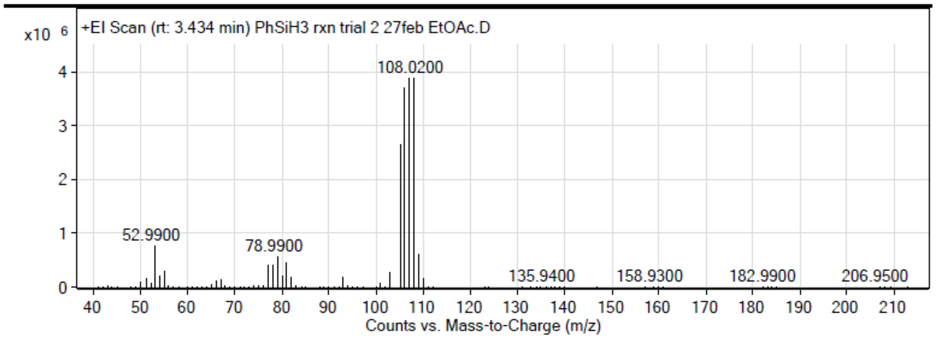

Figure 71. Mass spectrum of $PhSiH_3$ reaction

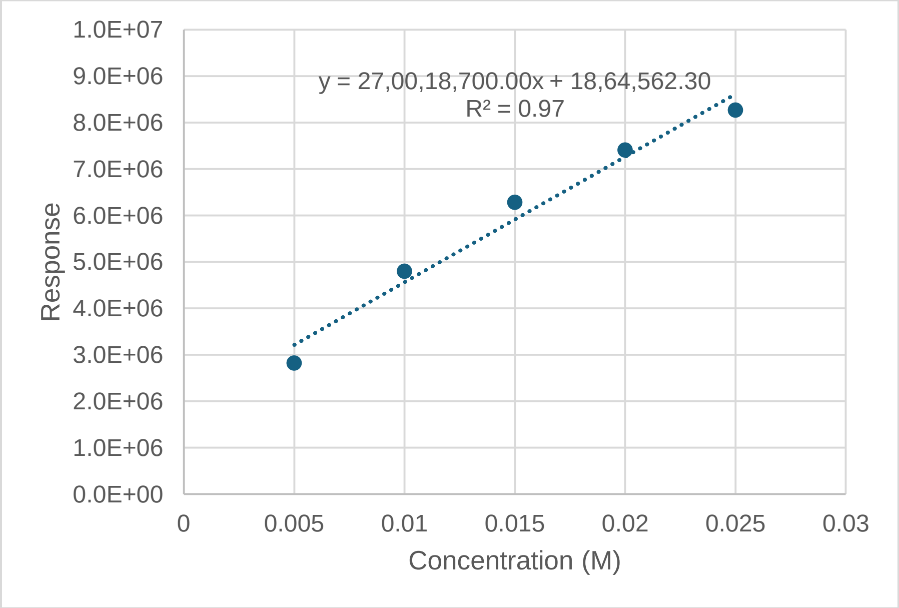

Figure 72. Calibration curve of $PhSiH_3$ in EtOAc

Dodecylsilane **2**

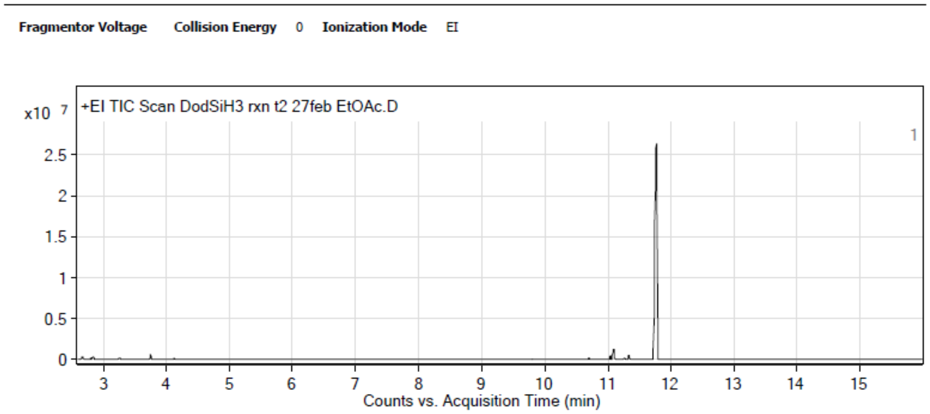

Figure 73. Chromatogram of dodecylsilane reaction

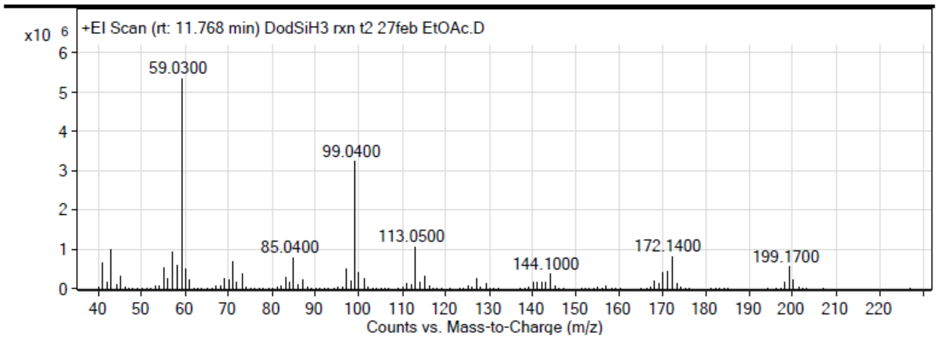

Figure 74. Mass spectrum of dodecylsilane reaction

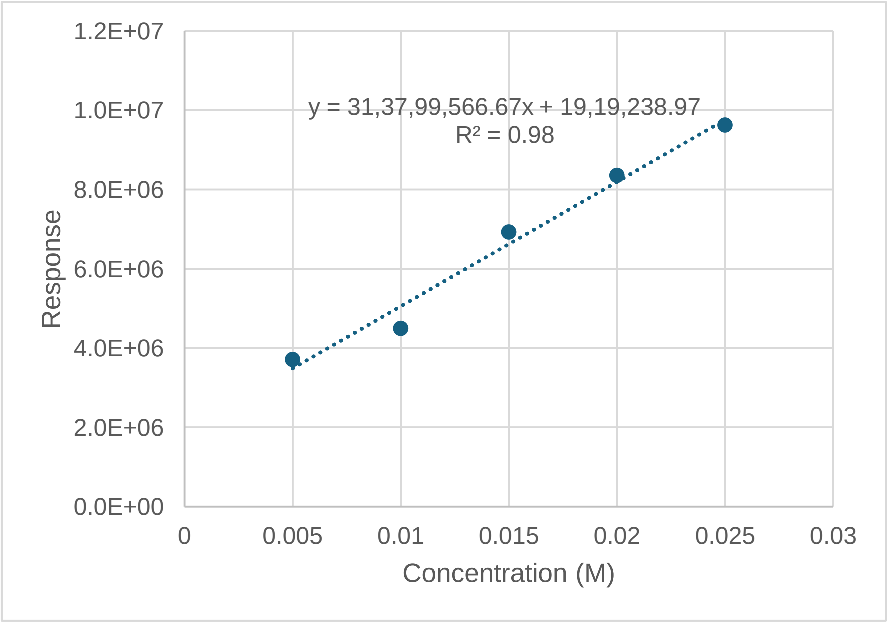

Figure 75. Calibration curve of dodecylsilane in EtOAc

Diphenylsilane **3**

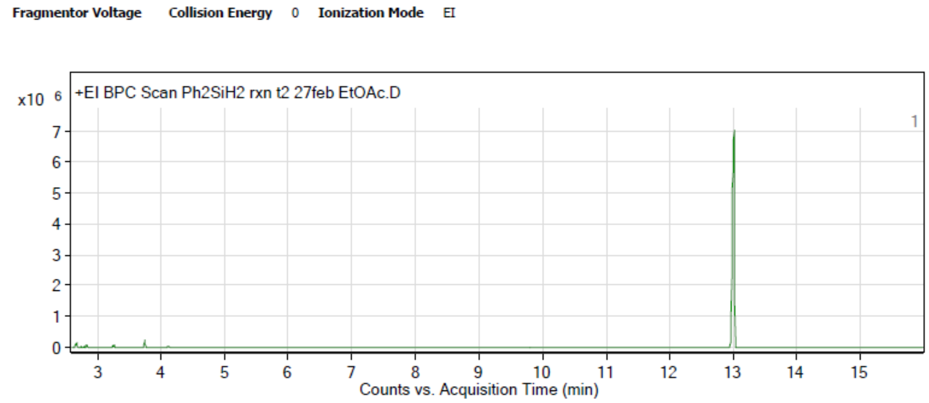

Figure 76. Chromatogram of Ph_2SiH_2 reaction

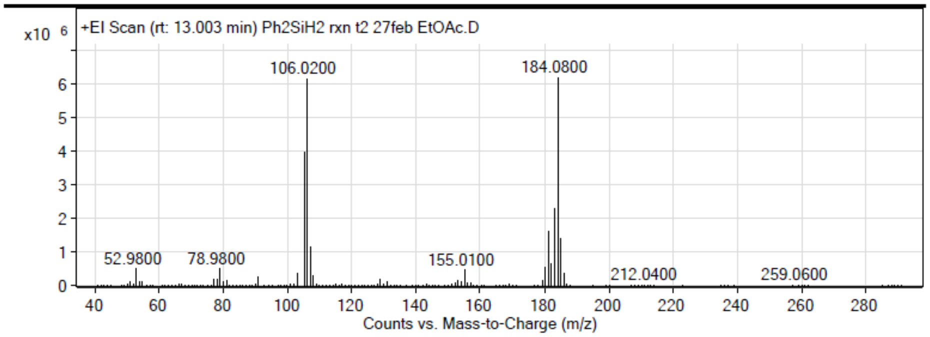

Figure 77. Mass spectrum of Ph_2SiH_2 reaction

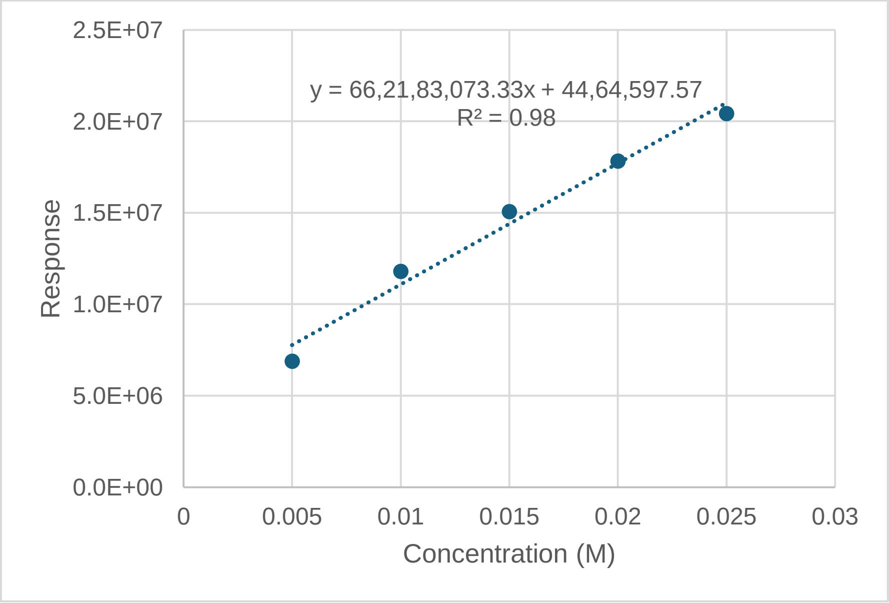

Figure 78. Calibration curve of Ph_2SiH_2 in EtOAc

Diethylsilane **4**

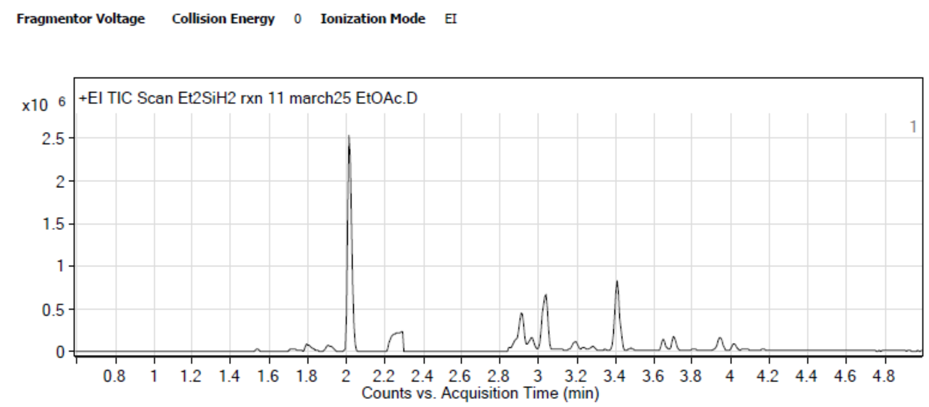

Figure 79. Chromatogram of Et_2SiH_2 reaction

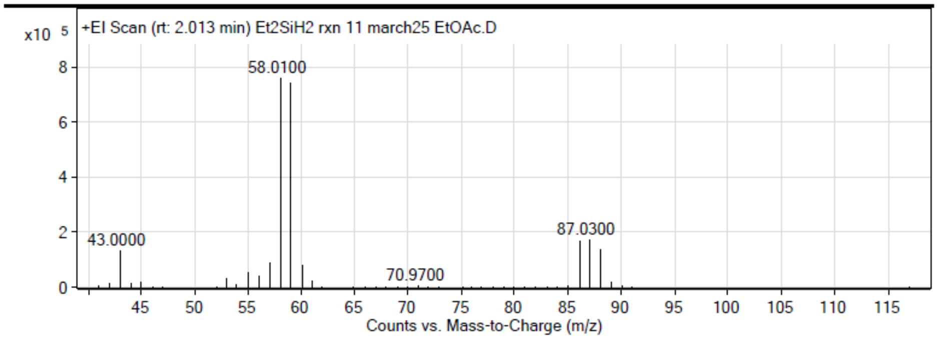

Figure 80. Mass spectrum of Et_2SiH_2 reaction

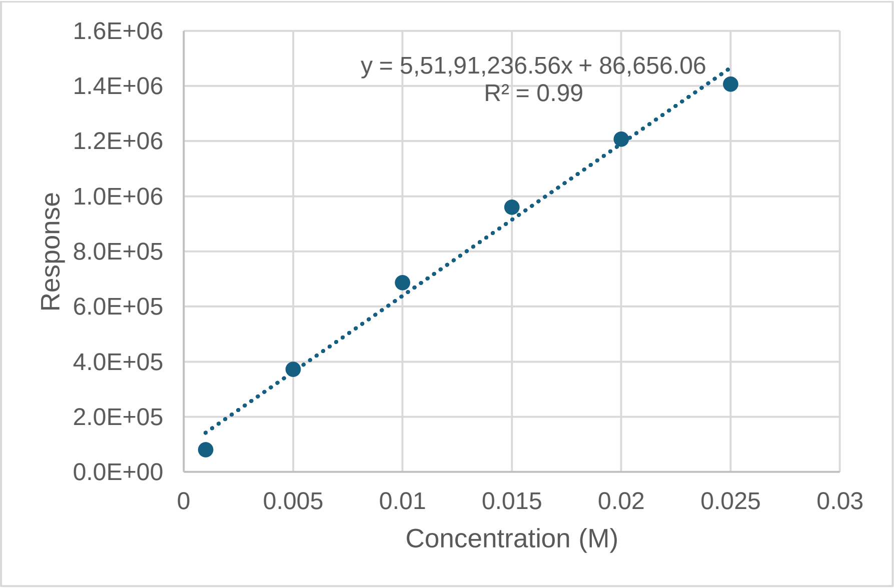

Figure 81. Calibration curve of Et_2SiH_2 in EtOAc

n-Hexylphenylsilane **5**

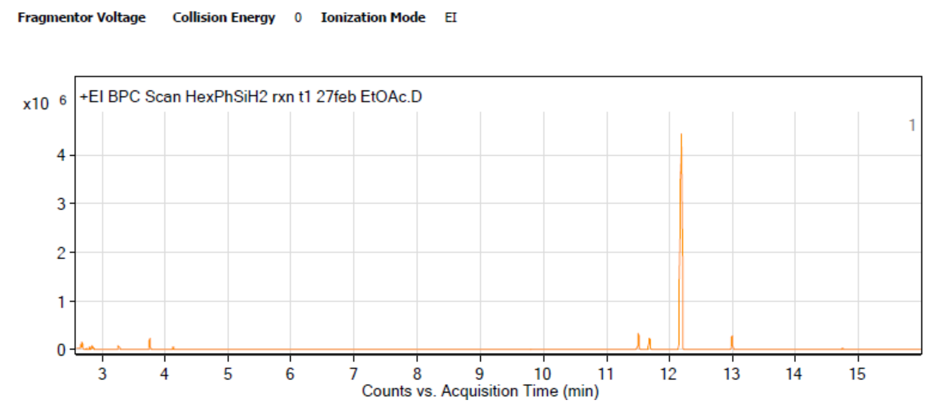

Figure 82. Chromatogram of Hex(Ph)SiH$_2$ reaction

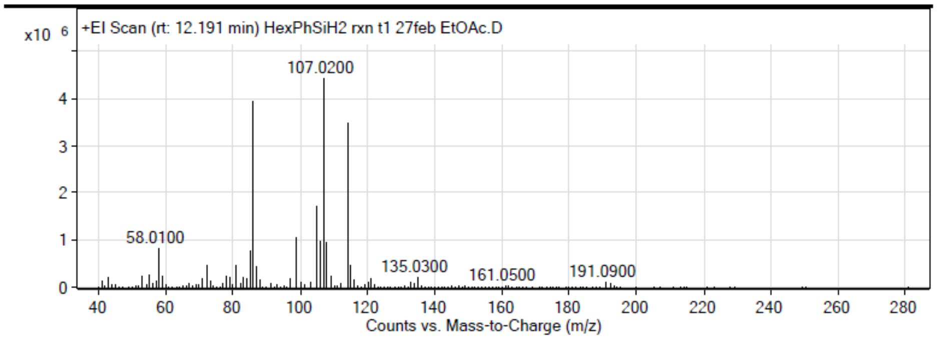

Figure 83. Mass spectrum of Hex(Ph)SiH$_2$ reaction

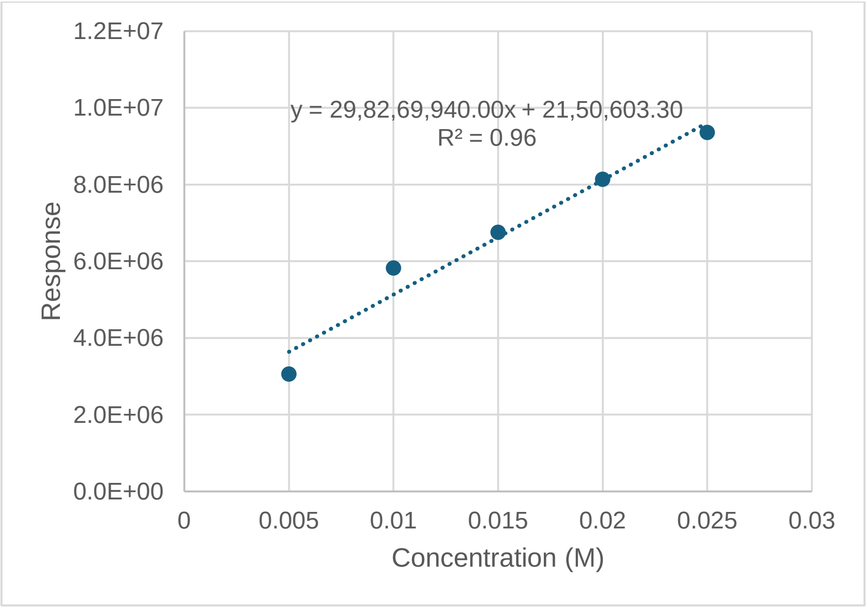

Figure 84. Calibration curve of Hex(Ph)SiH$_2$ in EtOAc

Triphenylsilane **6**

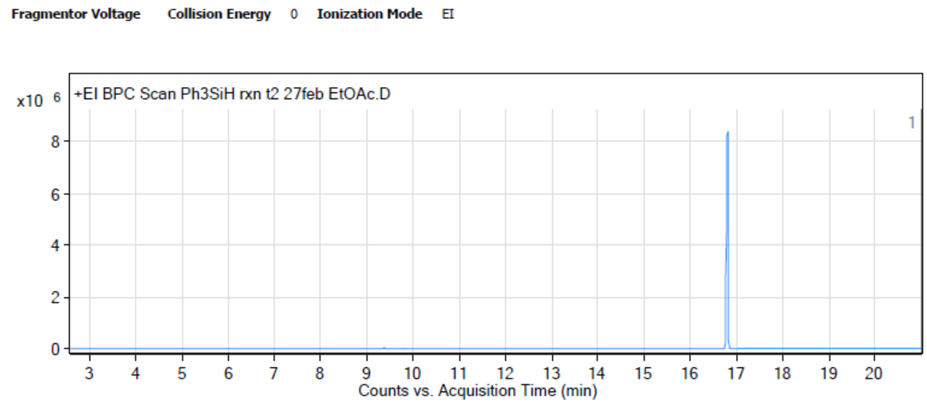

Figure 85. Chromatogram of Ph_3SiH reaction

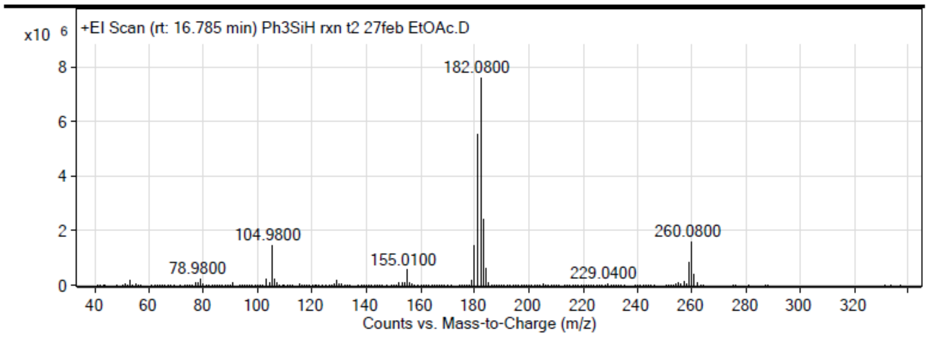

Figure 86. Mass spectrum of Ph_3SiH reaction

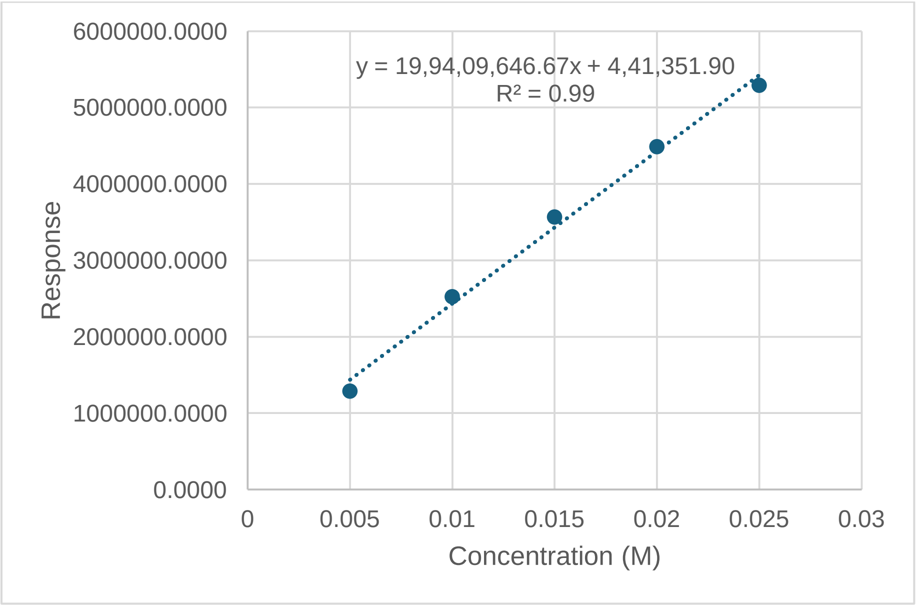

Figure 87. Calibration curve of Ph_3SiH in EtOAc

Triethylsilane **7**

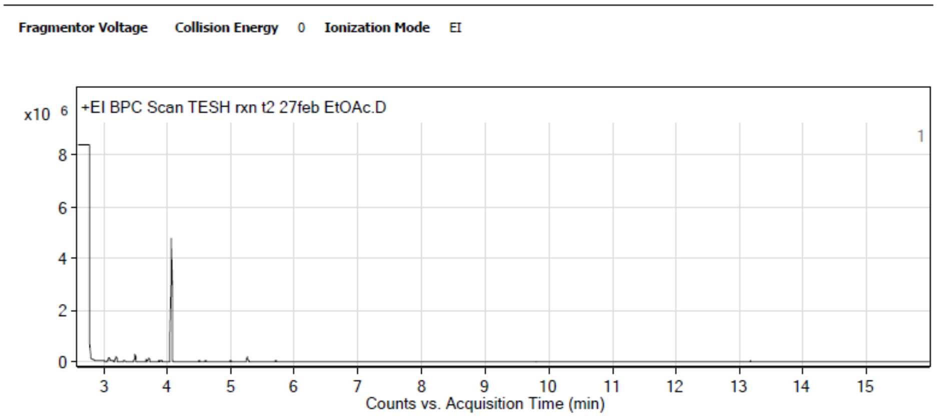

Figure 88. Chromatogram for Et_3SiH reaction

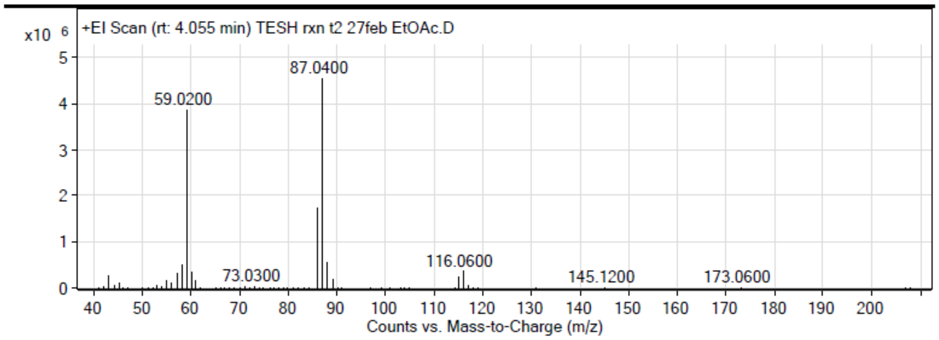

Figure 89. Mass spectrum of Et_3SiH reaction

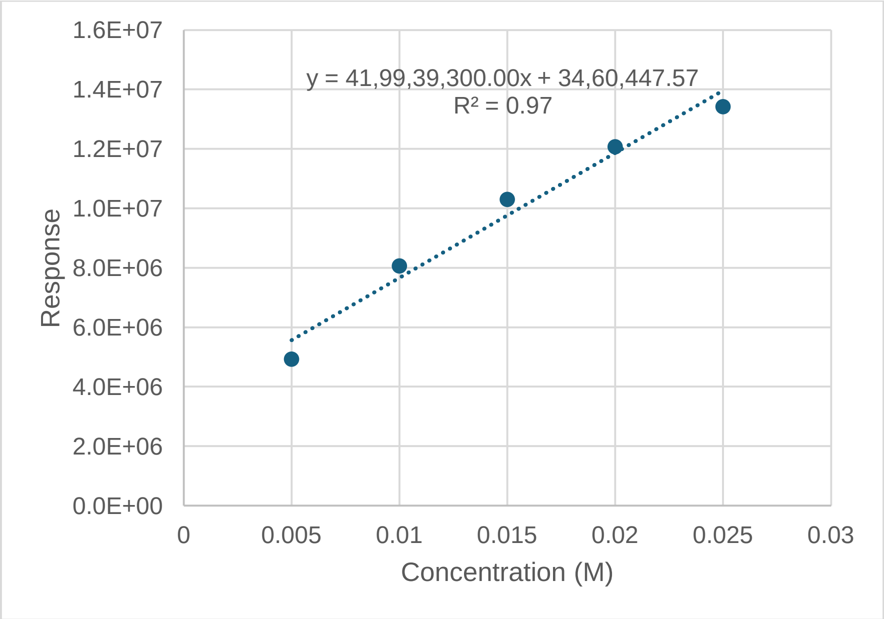

Figure 90. Calibration curve of Et_3SiH in EtOAc

Triisopropylsilane **8**

Fragmentor Voltage Collision Energy 0 Ionization Mode EI

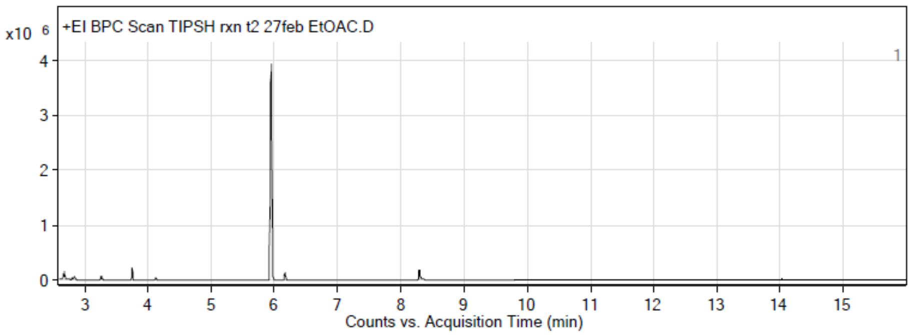

Figure 91. Chromatogram of iPr_3SiH reaction

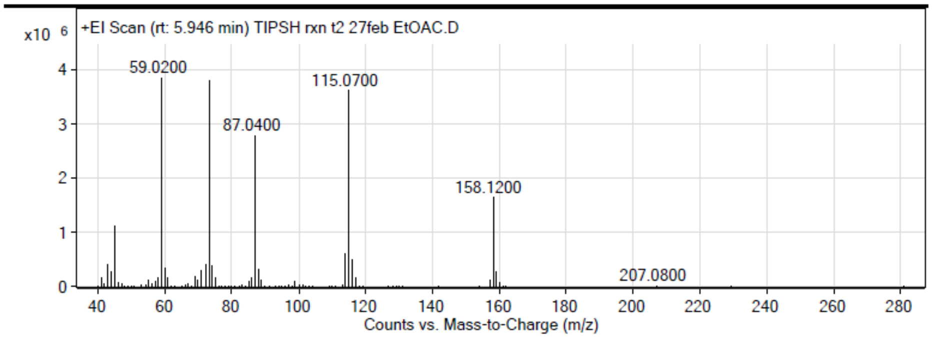

Figure 92. Mass spectrum of iPr_3SiH reaction

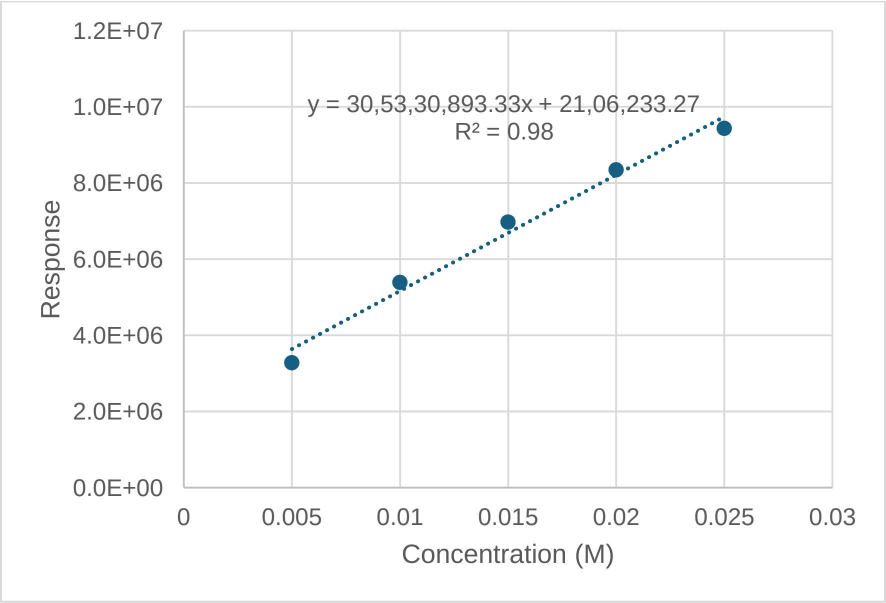

Figure 93. Calibration curve of iPr_3SiH in EtOAc

Dimethylphenylsilane **9**

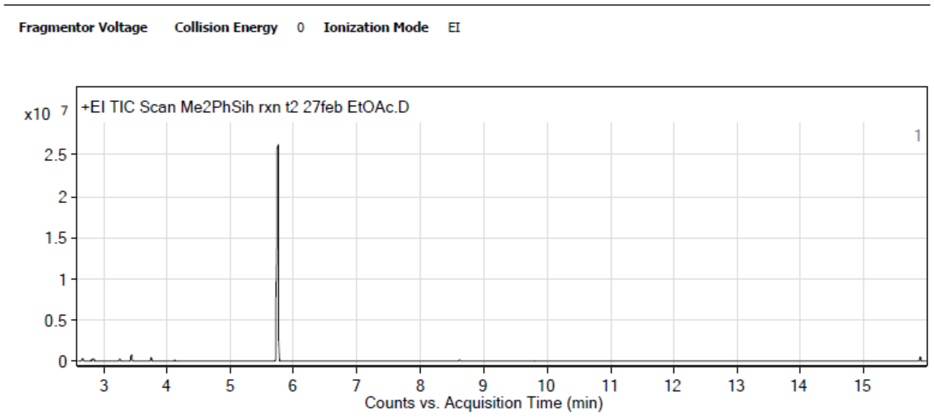

Figure 94. Chromatogram of Me_2PhSiH reaction

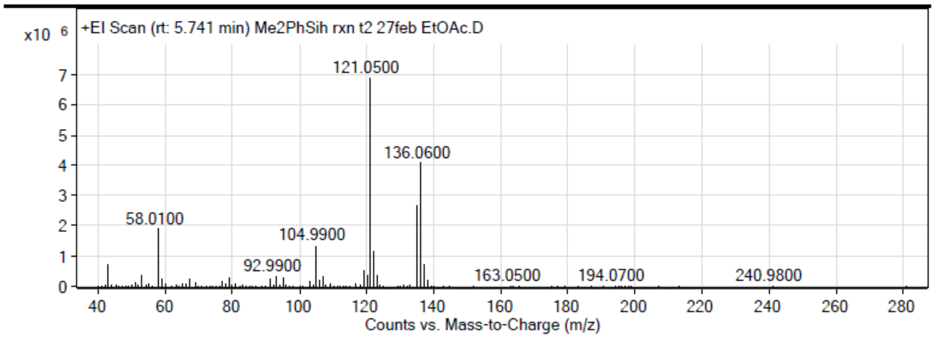

Figure 95. Mass spectrum of Me_2PhSiH reaction

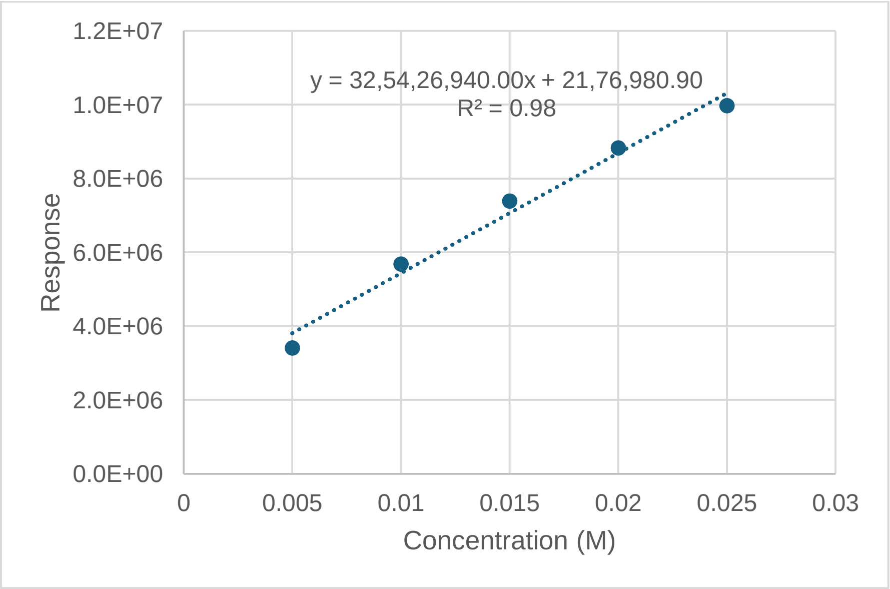

Figure 96. Calibration curve of Me_2PhSiH in EtOAc

Diphenylmethylsilane **10**

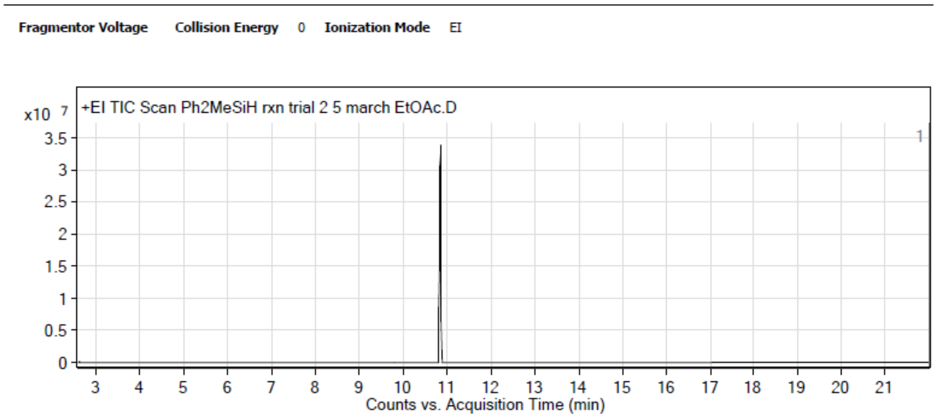

Figure 97. Chromatogram of Ph_2MeSiH reaction

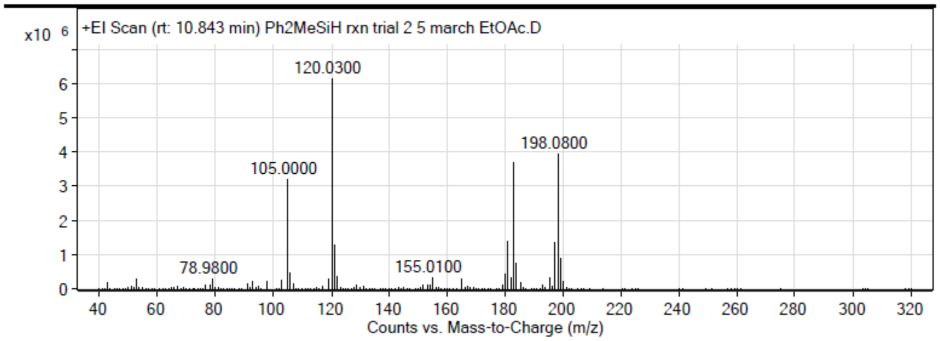

Figure 98. Mass spectrum of Ph_2MeSiH reaction

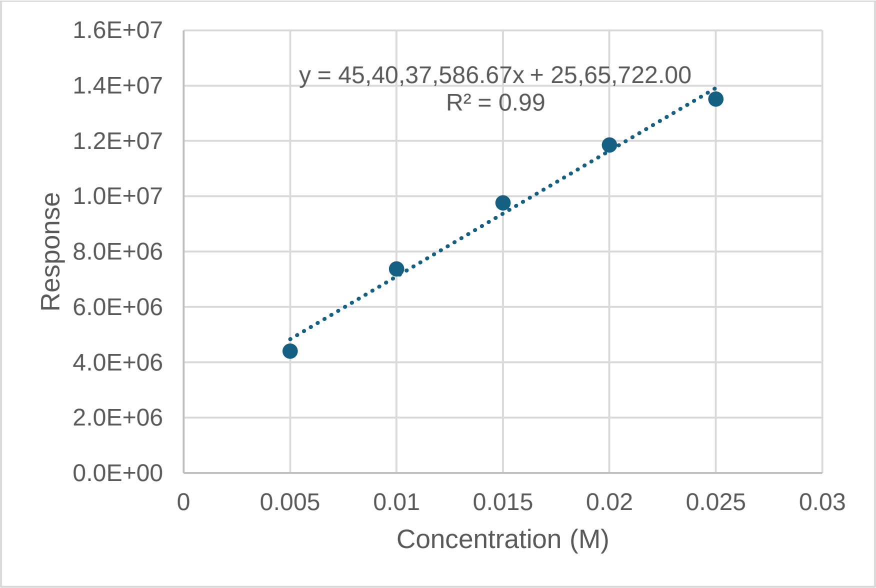

Figure 99. Calibration curve of Ph_2MeSiH in EtOAc

Reactivity in buffered aqueous solutions evaluated using ^{1}H-NMR (Study 5)

Experimental

To a flame-dried flask under nitrogen was added the silane (1 mmol) followed by PBS buffer solution (10 mL, 0.1 M, pH = 7.4). The reaction was stirred under nitrogen for 1 hour and then extracted using 5 x 5 mL EtOAc. The combined organic fraction was dried over $MgSO_4$, then concentrated under vacuum. The percent recovery was calculated based on the mass remaining after drying. The sample was dissolved in the appropriate deuterated solvent for ^{1}H-, ^{13}C-, ^{29}Si-NMR acquisition.

Dodecylsilane **2**

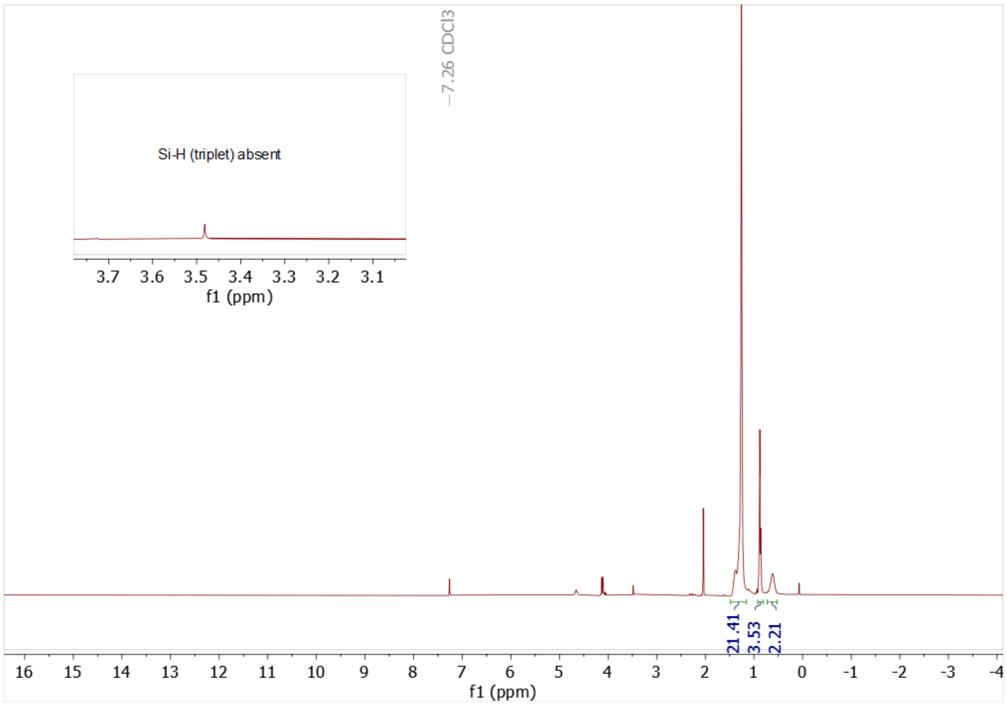

*Figure 100. ^{1}H-NMR ($CDCl_3$) of **2** after aqueous workup from PBS buffer solution.*

Diphenylsilane **3**

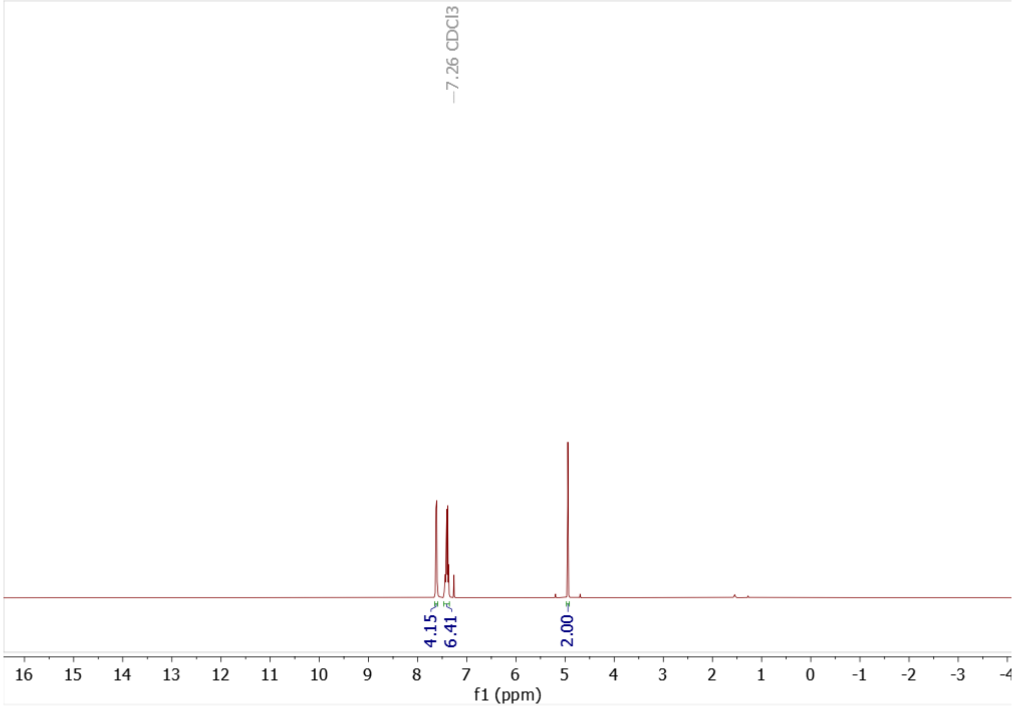

Figure 101. ^{1}*H-NMR (CDCl*$_3$*) of* ***3*** *after aqueous workup from PBS buffer solution.*

n-Hexylphenylsilane **5**

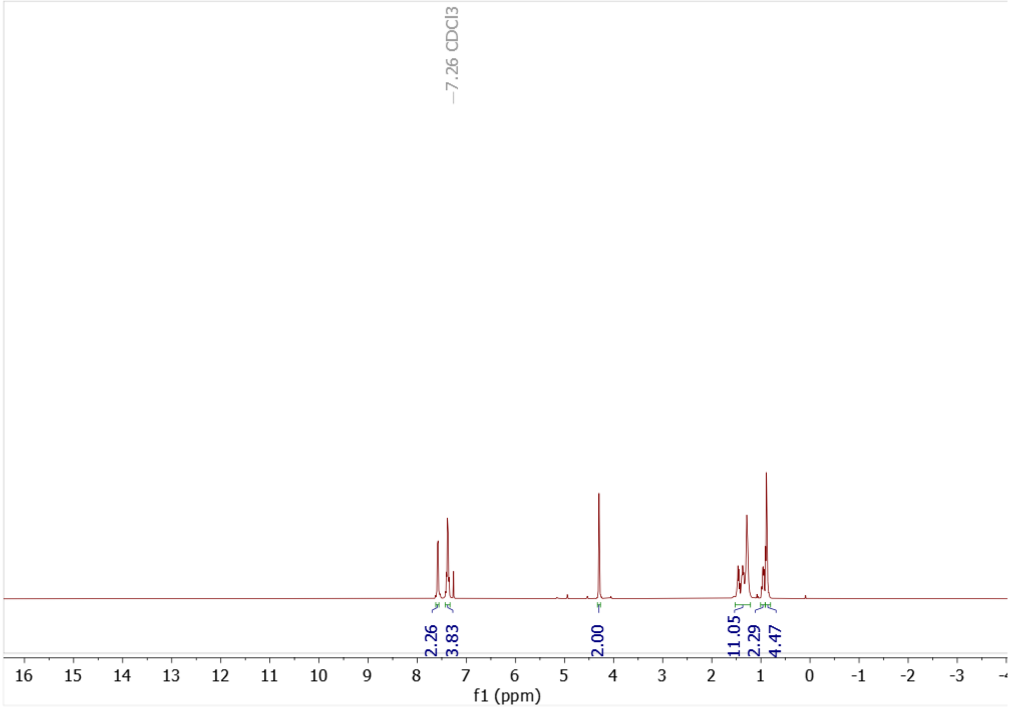

Figure 102. ^{1}*H-NMR (CDCl*$_3$*) of* ***5*** *after aqueous workup from PBS buffer solution.*

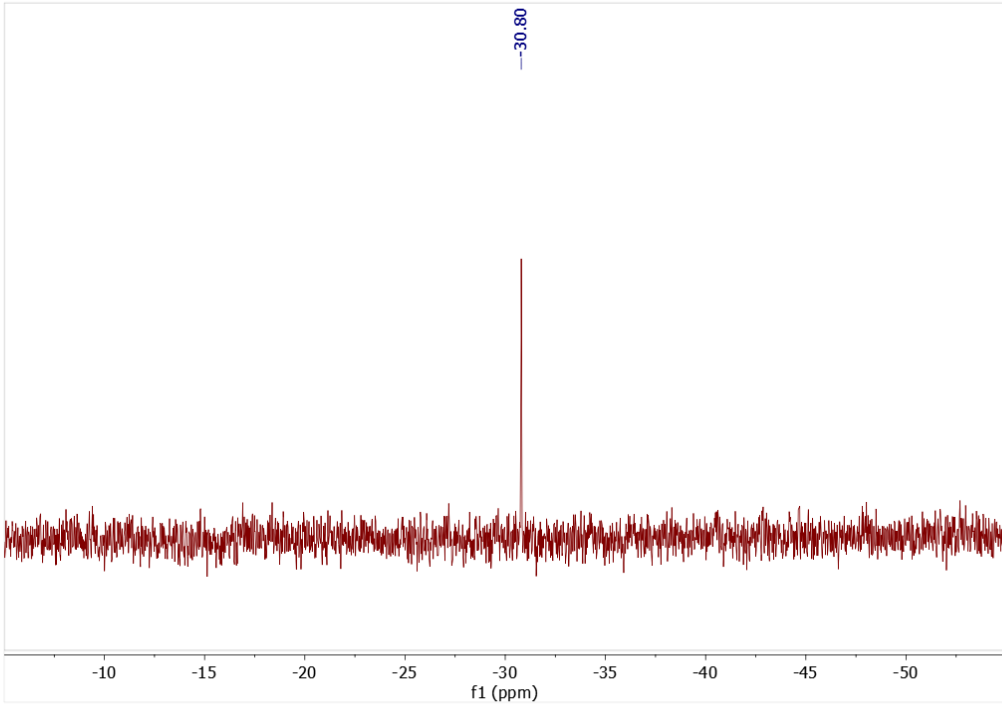

*Figure 103. ^{29}Si-NMR ($CDCl_3$) of **5** after aqueous workup from PBS buffer solution.*

Triphenylsilane **6**

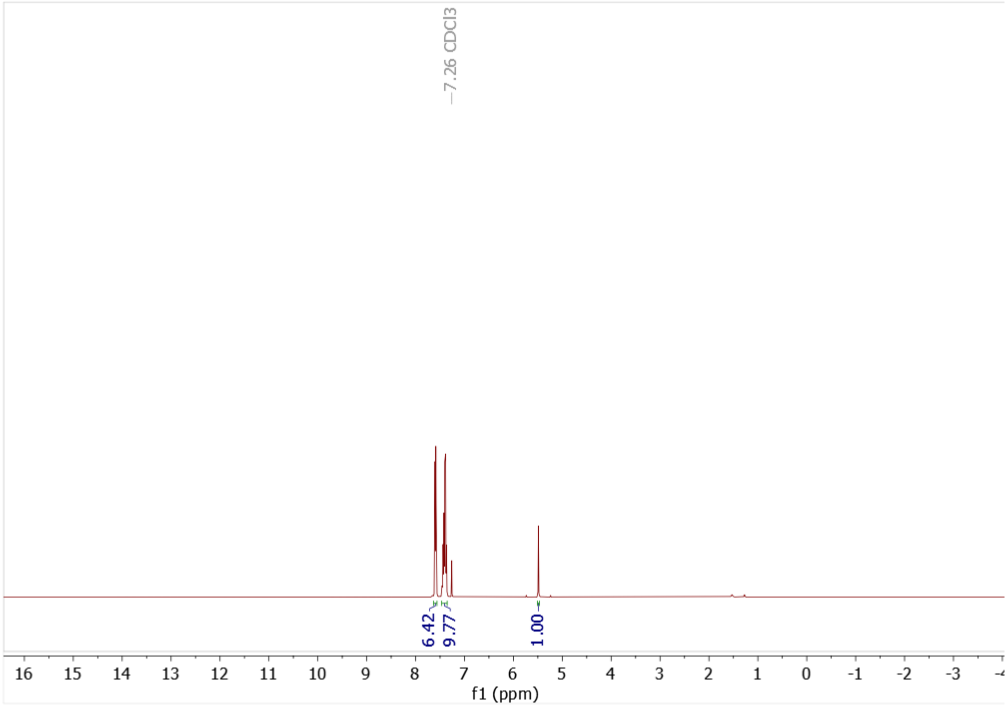

*Figure 104. ^{1}H-NMR ($CDCl_3$) of **6** after aqueous workup from PBS buffer solution.*

Diphenylmethylsilane **10**

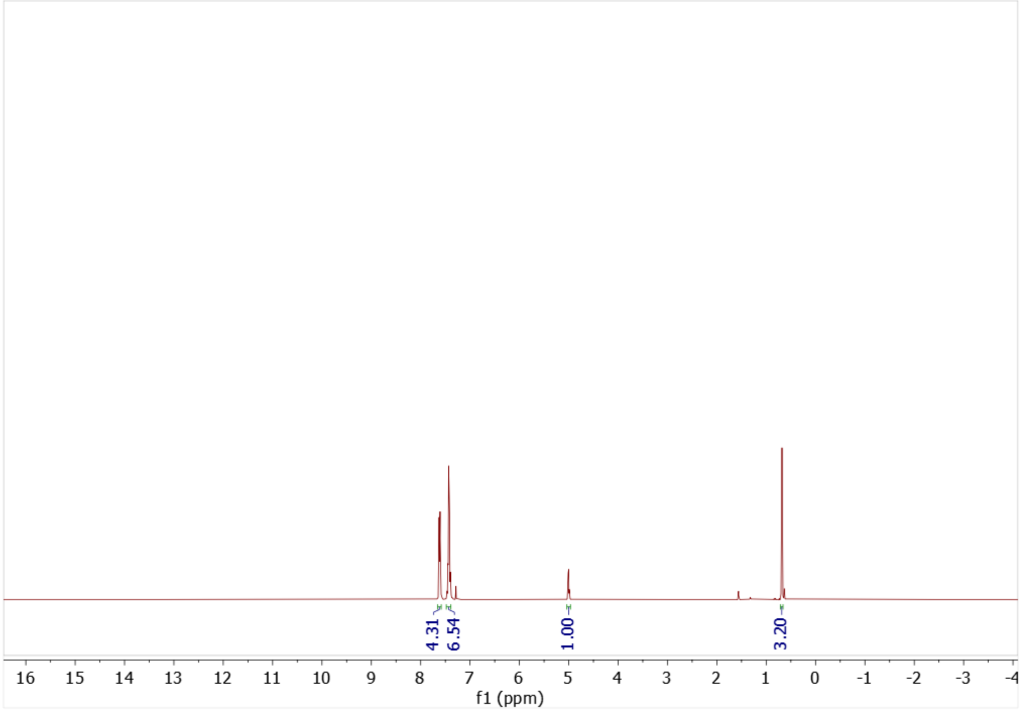

*Figure 105. ^{1}H-NMR ($CDCl_3$) of **10** after aqueous workup from PBS buffer solution.*

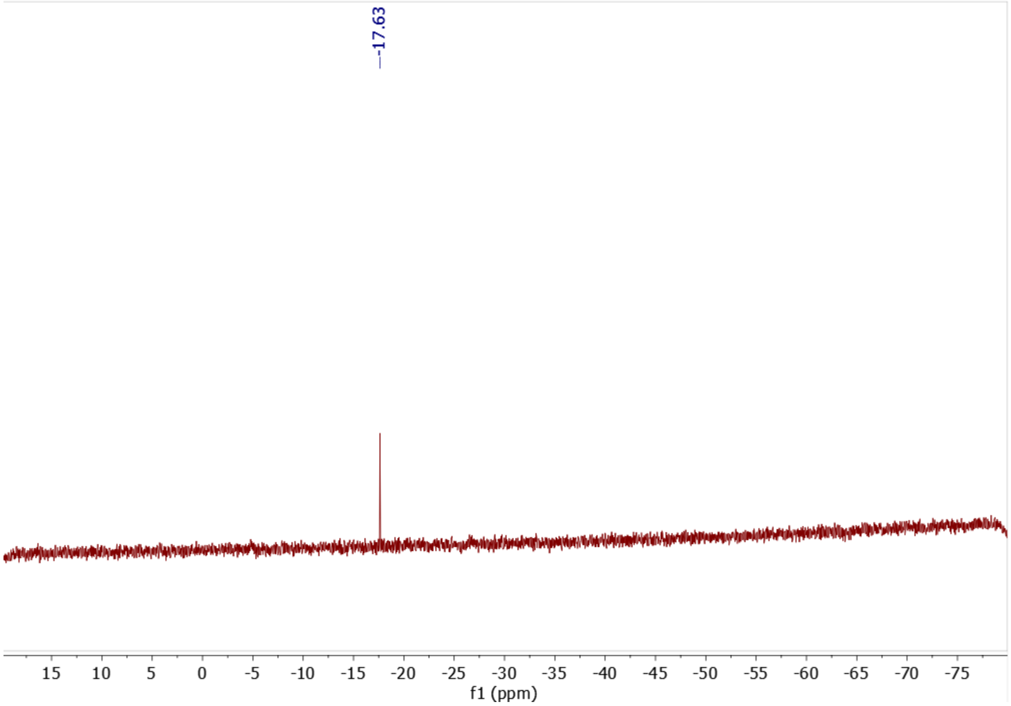

*Figure 106. ^{29}Si-NMR ($CDCl_3$) of **10** after aqueous workup from PBS buffer solution.*

MSDS information on moisture sensitivity of hydrosilanes

The MSDS information was obtained from Sigma-Aldrich, Gelest, Fisher Scientific, and TCI (accessed in March 2025). For each silane, the details about its "Handling and Storage" and "Stability and Reactivity" are quoted in this section.

$PhSiH_3$ **1**

- **Sigma-Aldrich**: "Tightly closed. Keep away from heat and sources of ignition. Storage class (TRGS 510): 4.3: Hazardous materials, which set free flammable gases upon contact with water. The product is chemically stable under standard ambient conditions (room temperature)."
- **Gelest**: "Decomposes slowly at room temperature generating hydrogen. The product can generate small amounts of hydrogen when exposed to alkalis and protic materials such as water and alcohol in combination with metal salts such as aluminum chloride or precious metals such as platinum. Keep container tightly closed."
- **Fisher Scientific**: "Handle under inert gas. Protect from moisture. Moisture sensitive. Keep away from open flames, hot surfaces and sources of ignition. Incompatible products. Exposure to moist air or water. Exposure to moisture."

dodecylSiH_3 **2**

- **Sigma-Aldrich**: Unavailable
- **Gelest**: The product can generate small amounts of hydrogen when exposed to alkalis and protic materials such as water and alcohol in combination with metal salts such as aluminum chloride or precious metals such as platinum. Keep container tightly closed. Stable in sealed containers under dry inert atmosphere."
- **Fisher Scientific**: Unavailable

Ph_2SiH_2 **3**

- **Sigma-Aldrich**: "Storage conditions Tightly closed. The product is chemically stable under standard ambient conditions (room temperature)."
- **Gelest**: "Storage area Store in a well-ventilated place. Store away from heat. Keep container tightly closed. Stable in sealed containers stored under a dry inert atmosphere. The product can generate small amounts of hydrogen when exposed to alkalis and protic materials such as water and alcohol in combination with metal salts such as aluminum chloride or precious metals such as platinum."
- **Fisher Scientific**: "Keep in a dry, cool and well-ventilated place. Keep container tightly closed. Store under an inert atmosphere. Incompatible Materials. Strong oxidizing agents. Conditions to Avoid: Incompatible products. Exposure to moist air or water."

Et_2SiH_2 **4**

- **Sigma-Aldrich**: “Keep container tightly closed in a dry and well-ventilated place. Containers which are opened must be carefully resealed and kept upright to prevent leakage. Incompatible materials: Strong oxidizing agents, Bases, Oxygen, Water.”

- **Gelest**: “Store in a well-ventilated place. Store away from heat. Stable in sealed containers stored under a dry inert atmosphere The product can generate small amounts of hydrogen when exposed to alkalis and protic materials such as water and alcohol in combination with metal salts such as aluminum chloride or precious metals such as platinum.”

- **Fisher Scientific**: “Keep container tightly closed in a dry and well-ventilated place. Keep away from heat, sparks and flame. Incompatible Materials. Oxidizing agent. Stability: Moisture sensitive.”

Hex(Ph)SiH_2 **5** *MSDS unavailable. MSDS for Me(Ph)SiH_2 is reported instead in this section.*

- **Sigma-Aldrich**: “Keep container tightly closed in a dry and well-ventilated place. The product is chemically stable under standard ambient conditions (room temperature). Conditions to avoid: Avoid moisture. Heating.”

Ph_3SiH **6**

- **Sigma-Aldrich**: “Keep container tightly closed in a dry and well-ventilated place.”

- **Gelest**: “Keep container tightly closed. Stable in sealed containers stored under a dry inert atmosphere. The product can generate small amounts of hydrogen when exposed to alkalis and protic materials such as water and alcohol in combination with metal salts such as aluminum chloride or precious metals such as platinum.”

- **Fisher Scientific**: “Keep in a dry, cool and well-ventilated place. Keep container tightly closed. Keep containers tightly closed in a dry, cool and well-ventilated place. Keep away from heat, sparks and flame. Incompatible Materials. Strong oxidizing agents. Oxidizing agent. Conditions to Avoid: Incompatible products. Exposure to moist air or water. Keep away from open flames, hot surfaces and sources of ignition.”

Et_3SiH **7**

- **Sigma-Aldrich**: “Keep container tightly closed in a dry and well-ventilated place. Keep away from heat and sources of ignition. The product is chemically stable under standard ambient conditions (room temperature).”

- **Gelest**: “Keep container tightly closed. Stable in sealed containers stored under a dry inert atmosphere. The product can generate small amounts of hydrogen when exposed to alkalis and protic materials such as water and alcohol in combination with metal salts such as aluminum chloride or precious metals such as platinum.”

- **Fisher Scientific**: “Keep container tightly closed. Keep containers tightly closed in a dry, cool and well-ventilated place. Stable under normal conditions. Decomposes in contact with water.”

i-Pr_3SiH **8**

- **Sigma-Aldrich**: “Keep container tightly closed. Keep container tightly closed in a dry and well-ventilated place. Keep away from heat and sources of ignition. Moisture sensitive. The product is chemically stable under standard ambient conditions (room temperature).”
- **Gelest**: “Store in a well-ventilated place. Store away from heat. Stable in sealed containers stored under a dry inert atmosphere. The product can generate small amounts of hydrogen when exposed to alkalis and protic materials such as water and alcohol in combination with metal salts such as aluminum chloride or precious metals such as platinum.”
- **Fisher Scientific**: “Keep in a dry, cool and well-ventilated place. Keep container tightly closed. Keep away from heat, sparks and flame. Keep away from acids. Flammables area. Keep container tightly closed in a dry and well-ventilated place. Incompatible Materials. Acids. Water. Stable. Moisture sensitive. Keep away from open flames, hot surfaces and sources of ignition. Incompatible products. Exposure to moist air or water.”

Me_2PhSiH **9**

- **Sigma-Aldrich**: “Keep container tightly closed in a dry and well-ventilated place. Keep away from heat and sources of ignition. Moisture sensitive. The product is chemically stable under standard ambient conditions (room temperature).”
- **Gelest**: “Keep container tightly closed. Store in a well-ventilated place. Store away from heat. Stable in sealed containers stored under a dry inert atmosphere. The product can generate small amounts of hydrogen when exposed to alkalis and protic materials such as water and alcohol in combination with metal salts such as aluminum chloride or precious metals such as platinum.”
- **Fisher Scientific**: “Keep containers tightly closed in a dry, cool and well-ventilated place. Moisture sensitive.”

Ph_2MeSiH **10**

- **Sigma-Aldrich**: Unavailable
- **Gelest**: Unavailable
- **TCI**: “Stable under normal conditions. Conditions to avoid: Exposure to air. Exposure to moisture. Incompatible materials: Oxidizing agents, Acids, Bases, water, Metals.”
- **Fisher Scientific**: “Keep in a dry, cool and well-ventilated place. Keep container tightly closed. Stable under normal conditions.”
